# Imidazopyridine-fused [1,3]diazepinones: modulations of positions 2 to 4 and their impacts on the anti-melanoma activity

**DOI:** 10.1080/14756366.2020.1748024

**Published:** 2020-04-05

**Authors:** Paul Le Baccon-Sollier, Yohan Malki, Morgane Maye, Lamiaa M. A. Ali, Laure Lichon, Pierre Cuq, Laure-Anaïs Vincent, Nicolas Masurier

**Affiliations:** a Institut des Biomolécules Max Mousseron, UMR 5247, CNRS, Universités Montpellier, UFR des Sciences Pharmaceutiques et Biologiques, Montpellier, France; b Department of Biochemistry, Medical Research Institute, University of Alexandria, Alexandria, Egypt

**Keywords:** Antitumor activity, diazepinones, imidazo[1,2-a]pyridine, melanoma, polyfused heterocycles

## Abstract

A series of 19 novel pyrido-imidazodiazepinones, with modulations of positions 2, 3 and 4 of the diazepine ring were synthesised and screened for their *in vitro* cytotoxic activities against two melanoma cell lines (A375 and MDA-MB-435) and for their potential toxicity against NIH-3T3 non-cancerous cells. Selected compounds were also evaluated on the NCI-60 cell line panel. The SAR study revealed that the molecular volume and the *c*LogP of compounds modified at position 2 were significantly correlated with the activity of these compounds on melanoma cell lines. Moreover, introduction of a heterocyclic group at position 2 or an azido-alkyl chain at position 4 led to compounds displaying a significantly different activity profile on the NCI-60 cell line panel, compared to phenyl-substituted compounds at position 2 of the diazepinone. This study provides us crucial information for the development of new derivatives active against melanoma.

## Graphical Abstract

**Figure:**
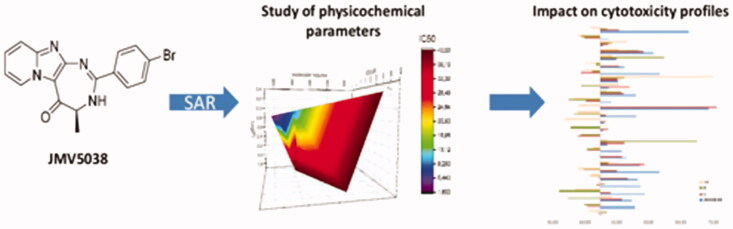

## Introduction

Melanoma is the most aggressive skin cancer and remains a therapeutic challenge. Its global incidence is increasing continuously, with 287,723 new cases in 2018, growing up to 301,694 in 2020 and 466,914 in 2040^1^^,^^2^. The prognosis of melanoma depends on how early the diagnosis is. While surgical resection of local forms is usually curative, metastatic melanoma (MM) requires systemic treatment and is associated with very poor prognosis. Indeed, the extreme chemoresistance of MM makes it less sensitive to conventional chemotherapy. Since 2011, new therapeutic strategies have emerged and offer promising results^3^. Small molecules, so-called “targeted” therapies, have been developed to specifically inhibit the V600E mutated BRAF kinase found in 50% of MM (Vemurafenib, Dabrafenib, Encorafenib)^4^. However, the rapid emergence of drug resistance to initially responsive cancers has led to only marginal patient benefit. To limit these resistances, mainly due to the reactivation of the MAPK pathway, MEK inhibitors (Trametinib, Cobimetinib, Binimetinib) are used in combination^5^. Nevertheless therapeutic escape are inevitably encountered^6^. In parallel, immunotherapies (inhibitors of CTLA4, PD1 and PDL1) have shown very interesting results, since some treated patients have not presented any relapse to date^7^. Unfortunately, no test can identify potentially responders and these therapies are not free of toxicity^8^. Finally, association between targeted therapies and immunotherapies confers a longer progression-free survival and duration of response, but led to a higher rate of serious toxicity^9^. Therefore, the development of new drugs that could be used in combination with mentioned therapies is still in demand to overcome the extreme chemoresistance of metastatic melanoma.

**Figure 1.**
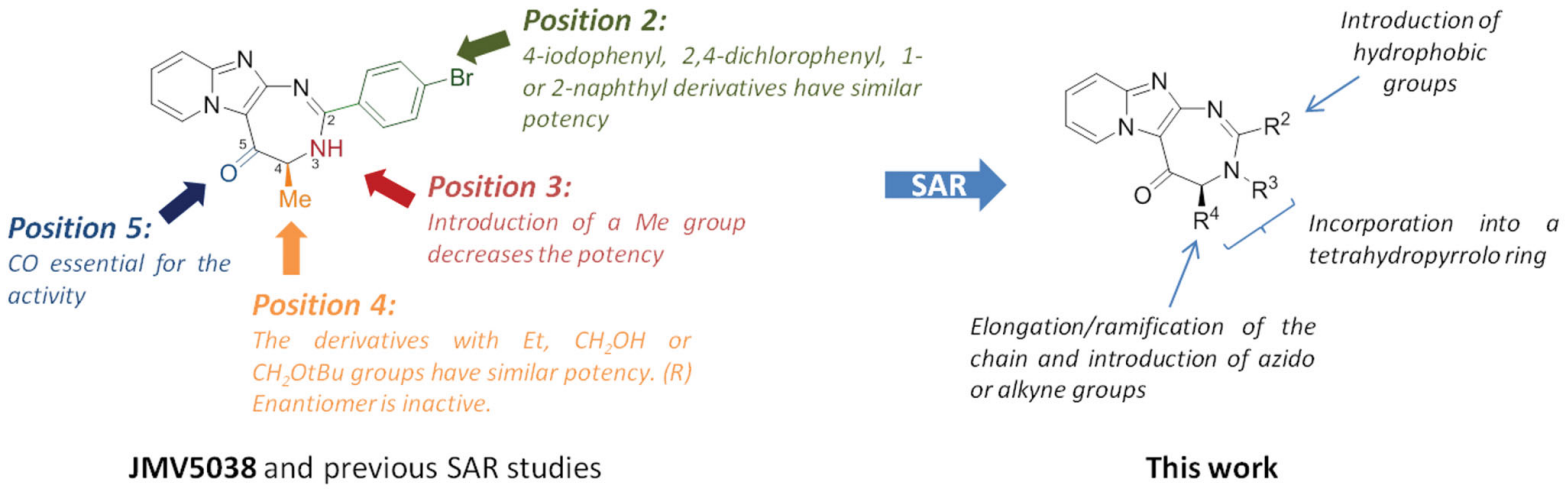
Structure of JMV5038, SAR studies from references^10^ and^11^ and new studied modulations.

Recently, we reported the discovery of JMV5038, an original pyrido-imidazodiazepinone derivative, using a screening on the NCI-60 cancer cell line panel^10^. This compound showed a promising activity on several cancer cell lines, with a growth inhibition of 50% (GI_50_) in the low micromolar range on melanoma cells and with no significant cytotoxic activity on NIH-3T3 fibroblasts, even at 100 µM. Further investigations revealed that JMV5038 increased apoptosis in subG1 phase and microscopic observations of treated cells showed morphological changes, associated with an increase of the membrane permeability and disruption of the actin network. We then started a structure-activity relationship (SAR) study on these series^11^. Whereas positions 3 and 5 of the diazepine ring could not be modified without loss of the cytotoxic activity, positions 2 and 4 of the diazepine cycle tolerated several modifications (Figure 1). Concerning position 2, we showed that large hydrophobic groups, such as a 1- or a 2-naphthyl ring, as well as halogenophenyl groups, did not modify the biological activity of JMV5038. For position 4, several modifications were tolerated with preservation of JMV5038 cytotoxic activity, like short elongation of the alkyl chain or introduction of bulky groups such as a *tert*-butyloxymethyl substituent (Figure 1). To further explore the SAR on these series, we decided to pursue the modulations of position 2 by introducing different hydrophobic groups and, of the position 4, with the introduction of different alkyl chains. Among others, we decided to study the introduction of azido or alkyne groups on these two positions, such groups could be of interest to investigate, in the future, further modulations using click chemistry. For example, such approach could permit to introduce a biotin residue to identify the pharmacological target of our compounds, using “clickable” cell-permeable activity-based probe^12^.

In this paper, we report the synthesis of new analogues of JMV5038. Their cytotoxic activity on melanoma cell lines and their potential toxicity against NIH-3T3 non-cancerous cells were evaluated. Subsequently, a statistical study was carried out to highlight the physicochemical parameters influencing the activity profile of our molecules. Selected compounds were then evaluated by the NCI on the sixty cancer cell lines panel. Further *in vitro* functional evaluations were finally performed on the most relevant compounds.

## Materials and methods

### Instruments and reagents

Commercially available reagents and solvents were used without further purification. Reactions were monitored by an analytical Waters Alliance 2690 HPLC instrument, equipped with a photodiode array and an analytical Chromolith Speed Rod RP-C18 185 Pm column (50 × 4.6 mm, 5 mm), at a flow rate of 3.0 ml/min, and gradients of 100/0–0/100 eluents A/B for 5 min (eluent A = H_2_O/0.1% TFA and B = CH_3_CN/0.1% TFA). Detection was performed at 214 nm. Retention times (Tr) are reported in min. ^1^H and 13 C NMR spectra were recorded on Bruker spectrometers (300 or 400 MHz) at room temperature in deuterated solvents. Chemical shifts (δ) are expressed in parts per million (ppm), relative to the resonance of CDCl_3_ = 7.26 ppm for ^1^H (77.16 ppm for 13 C), CD_3_OD = 3.31 ppm for ^1^H (49.00 ppm for 13 C) or DMSO d_6_ = 2.50 ppm for ^1^H (39.52 ppm for 13 C). The following abbreviations were used: s (singlet), d (doublet), t (triplet), q (quartet), m (multiplet), br (broad). Analytical thin-layer chromatography (TLC) was performed using 60 F254 aluminium-backed silica gel plates coated with a 0.2 mm thickness of neutral aluminium oxide. Column chromatography was performed on silica gel 60 (70–230 mesh) or on alumina gel (activated, neutral, Brockmann I). RP-preparative HPLC purification was performed on a Waters HPLC 4000 instrument, equipped with a UV detector 486 and Waters Delta-Pack 40´100 mm, 15 Å, 100 μm, reversed-phase column. Standard conditions were eluent system A (H_2_O/0.1% TFA), system B (CH_3_CN/0.1% TFA). A flow rate of 50 ml/min and a gradient of (0–40)% B over 30 min were used, detection was performed at 214 nm. The LC/MS system consisted of a Waters Alliance 2695 HPLC, coupled to a Micromass (Manchester, UK) ZQ spectrometer (positive electrospray ionisation mode, ESI+). All the analyses were carried out using a Merck Chromolith Speed rod C18, 25 × 4.6 mm reversed-phase column. A flow rate of 3 ml/min and a gradient of (0–100)% B over 3 min (or over 15 min) were used. Eluent A: water/0.1% HCO_2_H; eluent B: acetonitrile/0.1% HCO_2_H. Retention times (RT) are given in minutes. Nitrogen was used for both the nebulising and drying gas. The data were obtained in a scan mode ranging from 100 to 1000 *m/z* in 0.1 s intervals; 10 scans were summed up to get the final spectrum. High-resolution mass spectrometry analyses were performed with a Waters Synapt G2-S time-of-flight mass spectrometer fitted with an electrospray ionisation source. All measurements were performed in the positive ion mode. Melting points (Mp) are uncorrected and were recorded on a Stuart capillary melting point apparatus SMP3.

Compounds **2a**^13^**, 2c–d**^14^**, 2f–g**^14^, **3i**^14^, JMV5038^13^, Boc-azidonorleucine **29**^15^ and compound **23**^11^ were synthesised according to previous reported procedures and their physical characteristics were in agreement with the published data.

### Chemistry

#### Synthesis of 4-benzoyl-benzaldehyde 25

To a solution of 4-benzoylbenzoic acid (1 g, 4.42 mmol) in 30 ml of ethanol and 30 ml of cyclohexane, 2 ml of sulphuric acid were added. The solution was refluxed for one night. After cooling to room temperature, the solution was washed with a saturated NaHCO_3_ aqueous solution (2 × 30 ml). The organic layer was dried over Na_2_SO_4_, filtered, and the solvent was evaporated *in vacuo* to offer ethyl 4-benzoylbenzoate as a white powder (1.09 g, 97% yield), with spectral data in agreement with the literature^16^.

Ethyl 4-benzoylbenzoate (1 g, 3.94 mmol) was dissolved in 10 ml of dry THF and cooled to 0 °C. LiAlH_4_ (388 mg, 10.2 mmol, 2.6 equiv) was added by fractions and the mixture was stirred at 0 °C for 3 h. 50 ml of water were added and the solution was extracted with ethyl acetate (3 × 50 ml). The organic layer was dried over Na_2_SO_4_, filtered, and the solvent was evaporated *in vacuo*. The residue was then dissolved in 10 ml of chloroform and 4 equiv. of manganese(II) oxide were added. The mixture was stirred at reflux for 4 h. After cooling to room temperature, the mixture was filtered through a pad of celite and washed with chloroform. The filtrate was then evaporated *in vacuo.* The residue was purified by chromatography on silica gel, eluted by DCM/EtOH 99/1 v/v to offer 4-benzoyl-benzaldehyde **25** as a white solid (145 mg, 18% yield), with spectral data in agreement with the literature^17^; HPLC, RT= 1.67 min; MS (ESI^+^) calcd for C_14_H_11_O_2_: 211.1, found *m/z*: 211.1 [M + H]^+^

#### Synthesis of quinoline-6-carbaldehyde 26

To a solution of 6-quinolinecarboxylic acid (1 g, 5.71 mmol) in 40 ml of ethanol, 2 ml of sulphuric acid were added. The solution was refluxed for one night. After cooling to room temperature, 100 ml of water were added and the solution was basified using K_2_CO_3_. The solution was then extracted with dichloromethane (3 × 30 ml). The organic layer was dried over Na_2_SO_4_, filtered, and the solvent was evaporated *in vacuo* to offer ethyl 6-quinolinecarboxylate **25** (1.1 g, 95% yield) as a white powder. Spectral data were in agreement with the literature^18^.

Ester **25** (1.1 g, 5.47 mmol) was then dissolved in 10 ml of dry THF and cooled to 0 °C. LiAlH_4_ (375 mg, 10.9 mmol, 2 equiv) was added by fractions and the mixture was stirred at 0 °C for 2 h. 50 ml of water were added and the solution was extracted with ethyl acetate (2 × 50 ml). The organic layer was dried over Na_2_SO_4_, filtered, and the solvent was evaporated *in vacuo*. The residue was then dissolved in 10 ml of chloroform and 4 equiv. of manganese(II) oxide were added. The mixture was stirred at reflux for 4 h. After cooling to room temperature, the mixture was filtered through a pad of celite and washed with chloroform. The filtrate was then evaporated *in vacuo.* The residue was purified by chromatography on silica gel, eluted by DCM/nHexane 9/1 v/v to offer 6-quinolinecarboxaldehyde **26** as a white solid (343 mg, yield 30%); mp: 73–75 °C (litt.^19^ 75–76 °C); ^1^H NMR (CDCl_3_, 300 MHz): δ ppm 7.50 (dd, 1H, *J* = 8.3 Hz, 8.3 Hz), 8.18 (m, 2H), 8.29–8.33 (m, 2H), 9.03 (dd, 1H, *J* = 4.2 Hz, 1.7 Hz), 10.18 (s, 1H); 13 C NMR (CDCl_3_, 75 MHz): δ ppm 122.4, 127.0, 127.9, 130.8, 131.0, 133.8, 134.5, 137.7, 153.3, 191.7; HPLC, Tr= 0.84 min; MS (ESI^+^): calcd for C_10_H_8_NO 158.1, found *m/z* 157.8 [M + H]^+^.

#### Synthesis of compounds 2

To a suspension of trifluoro-*N*-imidazo[1,2-*a*]pyridin-2-acetamide^13^ (0.5 g, 2.18 mmol) in 5 N aqueous sodium hydroxide (9 ml), was added THF (0.5 ml). The solution was stirred at 40 °C for 2 h. The solution was extracted with dichloromethane (3 × 20 ml). The organic layer was dried over Na_2_SO_4_, filtered, and the solvent was removed *in vacuo*. The residue was dissolved in dichloromethane (20 ml) and either Boc-azidonorleucine **29** or Boc-Pra-OH or Boc-Nle-OH (2.4 mmol, 1.1 equiv), HOBt (325 mg, 2.4 mmol, 1.1 equiv), EDCI (460 mg, 2.4 mmol, 1.1 equiv), or triethylamine (334 ml, 2.4 mmol, 1.1 equiv), were added at 0 °C. The solution was stirred at room temperature for one night. The solution was then washed with a saturated NaHCO_3_ solution (2 × 50 ml). The organic layer was dried over Na_2_SO_4_, filtered, and the solvent was concentrated *in vacuo*. The residue was purified by chromatography (Al_2_O_3_, DCM/EtOH 99/1 v/v for compound **2b** and **2h** or SiO_2_, DCM/AcOEt 9/1 v/v for compound **2e**).

#### (2S)-2-Boc-amino-1-(2-aminoimidazo[1,2-a]pyridin-3-yl)-hexan-1-one (2 b)

Beige solid (536 mg, yield 71%); mp: 174–175 °C; [α]_D_^20^ = −7.9° (*c* 1.1, CHCl_3_); ^1^H NMR (CDCl_3_, 400 MHz): δ ppm 0.83 (t, 3H, *J* = 6.7 Hz), 1.28 (m, 4H), 1.40 (s, 9H), 1.67 (m, 1H), 1.85 (m, 1H), 4.92 (q, 1H, *J* = 7.2 Hz), 5.50 (d, 1H, *J* = 9.0 Hz), 6.01 (br, 2H), 6.85 (td, 1H, *J* = 6.8 Hz, 1.3 Hz), 7.33 (d, 1H, *J* = 8.6 Hz), 7.39 (dd, 1H, *J* = 8.6 Hz, 6.8 Hz), 9.63 (br, 1H); 13 C NMR (CDCl_3_, 75 MHz): δ ppm 14.1, 22.7, 28.0, 28.5, 54.5, 80.5, 108.3, 113.2, 114.4, 129.5, 130.7, 147.9, 156.9, 158.9, 186.1; HPLC, Tr= 1.62 min; MS (ESI^+^) calcd for C_18_H_27_N_4_O_3_ 347.2, found *m/z* 347.2 [M + H]^+^.

#### (2S)-2-Boc-amino-1-(2-aminoimidazo[1,2-a]pyridin-3-yl)pent-4-yn-1-one (2e)

White solid (257 mg, yield 36%); mp: 147–148 °C; [α]_D_^20^ = −59.1° (*c* 0.1, CHCl_3_); ^1^H NMR (CDCl_3_, 300 MHz): δ ppm 1.41 (s, 9H), 1.96 (t, 1H, *J* = 2.6 Hz), 2.70 (ddd, 2H, *J* = 6.8 Hz, 6.8 Hz, 2.6 Hz), 5.14 (m, 1H), 5.60 (d, 1H, *J* = 9.7 Hz), 6.86 (td, 1H, *J* = 6.8 Hz, 1.3 Hz), 7.33 (d, 1H, *J* = 8.6 Hz), 7.41 (dd, 1H, *J* = 8.6 Hz, 6.8 Hz), 9.59 (br, 1H); 13 C NMR (CDCl_3_, 75 MHz): δ ppm 22.6, 28.4, 55.6, 71.0, 79.8, 81.0, 98.5, 113.4, 114.4, 129.7, 131.2, 148.0, 156.6, 158.9, 182.9; HPLC, RT= 1.33 min; MS (ESI^+^) calcd for C_17_H_21_N_4_O_3_ 329.1, found *m/z* 329.0 [M + H]^+^.

#### (2S)-2-Boc-amino-1-(2-aminoimidazo[1,2-a]pyridin-3-yl)-6-azidohexan-1-one (2 h)

White solid (340 mg, yield 70%); mp: 74–76 °C; [α]_D_^20^ = +1.3° (*c* 1.0, CHCl_3_); ^1^H NMR (CDCl_3_, 300 MHz): δ ppm 1.12–1.21 (m, 1H), 1.34 (m, 10H), 1.50 (m, 2H), 1.70 (m, 1H), 1.84 (m, 1H), 3.13 (t, 1H, *J* = 6.7 Hz), 4.92 (q, 1H, *J* = 7.2 Hz), 6.37 (br, 3H), 6.80 (m, 1H), 7.33 (m, 2H), 9.60 (d, 1H, *J* = 7.8 Hz); 13 C NMR (CDCl_3_, 75 MHz): δ ppm 22.9, 28.3, 28.6, 32.0, 51.1, 54.1, 80.3, 108.0, 113.1, 114.1, 129.4, 131.0, 147.8, 157.0, 159.0, 185.4; HPLC, RT= 1.38 min; MS (ESI^+^) calcd for C_18_H_26_N_7_O 388.2, found *m/z* 387.8 [M + H]^+^.

#### General procedure for the synthesis of diazepinones 4–20

##### Method A (for compounds 5–13 and 15–20)

A solution of compound **2a–i** (0.33 mmol) in a 50/50 v/v mixture of trifluoroacetic acid and dichloromethane (5 ml) was stirred at room temperature for 1 h. After completion of the reaction (HPLC analysis), the solution was concentrated to dryness and co-evaporated several times with acetonitrile to completely remove TFA. Then, were added THF (8 ml), K_2_CO_3_ (182 mg, 4 equiv.), iodine (251 mg, 3 equiv.) and the appropriate aldehyde (1 equiv.). The mixture was stirred under heating at 70 °C for one night. THF was then removed under reduce pressure and 30 ml of dichloromethane were added. The organic phase was successively washed with a 10% m/v sodium thiosulphate aqueous solution (2 × 30 ml) and then with a saturated NaHCO_3_ aqueous solution (2 × 30 ml). The organic layer was dried over Na_2_SO_4_, filtered, and the solvent was evaporated *in vacuo*. The residue was purified by chromatography on alumina, eluted by DCM, DCM/EtOH 99/1 v/v and finally DCM/EtOH 98/2 v/v.

##### Method B (for compounds 4 and 14)

A solution of compound **2a** or compound **2e** (0.33 mmol) in a 50/50 v/v mixture of trifluoroacetic acid and dichloromethane (5 ml) was stirred at room temperature. After completion of the reaction (HPLC analysis), the solution was concentrated to dryness and co-evaporated several times with acetonitrile to completely remove TFA. Then, 5 ml of water were added and the solution was basified by K_2_CO_3_ and then extracted by chloroform (3 × 10 ml). The organic layer was dried over Na_2_SO_4_, filtered and the solvent was evaporated *in vacuo*. The crude was then dissolved in 20 ml of chloroform and 1 equiv of the appropriate aldehyde was added. The solution was stirred at 80 °C for 6 h. After cooling, DDQ (0.44 mmol, 2 equiv) was added to the reaction mixture. The solution was stirred at room temperature for 6 h (monitoring by TLC). The solution was washed with aqueous saturated sodium hydrogen carbonate solution (2 × 30 ml). The organic layer was dried over Na_2_SO_4_, filtered, and the solvent was evaporated *in vacuo*. The residue was purified by chromatography on alumina, eluted by DCM and then DCM/EtOH 99/1 v/v for compound **4** or by preparative HPLC for compound **14** (gradient of 100% H_2_O/0.1% TFA to 40% CH_3_CN/0.1% TFA).

##### (4S)-4-methyl-2-(4-trimethylsilylethynylphenyl)-3,4-dihydro-5H-pyrido[1’,2’:1,2]imidazo[4,5-d][1,3]diazepin-5-one (4)

Brown solid (23 mg, yield 18%); mp: 115–116 °C; [α]_D_^20^ = +43.8° (*c* 0.4, CHCl_3_); ^1^H NMR (CDCl_3_, 500 MHz): 2 conformers δ ppm 0.23 (s, 4.5H), 0.24 (s, 4.5H), 1.70 (d, 3H, *J* = 6.6 Hz), 4.10 (m, 1H), 7.0 (dd, 1H, *J* = 6.7 Hz, 6.6 Hz), 7.41 (m, 1H), 7.49 (d, 1H, *J* = 8.2 Hz), 7.53 (d, 1H, *J* = 8.2 Hz), 7.66 (d, 1H, *J* = 8.6 Hz), 7.87 (d, 1H, *J* = 8.2 Hz), 7.99 (d, 1H, *J* = 8.2 Hz), 9.47 (d, 0.5H, *J* = 6.6 Hz), 9.55 (d, 0.5H, *J* = 6.6 Hz), 9.97 (br, 1H); 13 C NMR (CDCl_3_, 125 MHz): 2 conformers δ ppm 15.5, 57.4, 97.4, 97.9, 104.2, 113.8, 115.2, 117.0, 126.5, 126.8, 127.1, 128.6, 128.8, 130.2, 130.5, 132.3, 132.5, 134.6, 135.4, 145.5, 147.3, 152.6, 152.9, 157.6, 158.3, 182.5, 183.2; HPLC, RT= 1.32 min; MS (ESI^+^) calcd for C_22_H_23_N_4_OSi 387.1, found *m/z* 387.0 [M + H]^+^.

##### (4S)-4-methyl-2-(4-azidophenyl)-3,4-dihydro-5H-pyrido[1’,2’:1,2]imidazo[4,5-d][1,3]diazepin-5-one (5)

Brown solid (28 mg, yield 26%); mp: 235–236 °C (dec.); [α]_D_^20^ = +113° (*c* 0.1, CHCl_3_); ^1^H NMR (CDCl_3_, 500 MHz): δ ppm 1.69 (br, 3H), 4.09 (q, 1H, *J* = 6.6 Hz), 6.08 (br, 1H), 7.00 (t, 1H, *J* = 7.0 Hz), 7.05 (d, 1H, *J* = 7.9 Hz), 7.45 (br, 2H), 7.65 (br, 1H), 7.95 (m, 2H), 9.51 (d, 1H, *J* = 7.0 Hz); ^13^C NMR (CDCl_3_, 125 MHz): δ ppm 35.2, 62.7, 113.9, 116.9, 119.4, 123.1, 123.4, 125.5, 126.4, 128.7, 130.3, 130.7, 152.8, 155.5, 181.7; HPLC, RT= 1.05 min; MS (ESI^+^): *m/z* 332.1 [M + H]^+^; HRMS calcd for C_17_H_14_N_7_O 332.1260, found 332.1262.

##### (4S)-4-methyl-2-(4-benzoylphenyl)-3,4-dihydro-5H-pyrido[1’,2’:1,2]imidazo[4,5-d][1,3]diazepin-5-one (6)

Yellow solid (45 mg, yield 35%); mp: 132–133 °C; [α]_D_^20^ = +18.7° (*c* 1.0, MeOH); ^1^H NMR (DMSO d_6_, 500 MHz): δ ppm 1.35 (br, 1.8H), 1.54 (br, 1.2H), 4.05 (q, 1H, *J* = 6.7 Hz), 7.19 (dd, 1H, *J* = 6.6 Hz, 7.9 Hz), 7.60 (t, 2H, *J* = 7.6 Hz), 7.63–7.73 (m, 3H), 7.78 (d, 2H, *J* = 7.0 Hz), 7.87 (m, 2H), 8.20 (d, 1H, *J* = 6.4 Hz), 8.77 (s, 1H), 9.39 (br, 0.4H), 9.45 (br, 0.6H); 13 C NMR (DMSO d_6_, 125 MHz): δ ppm 15.5, 56.6, 113.8, 116.0, 127.8, 128.6, 128.7, 129.3, 129.5, 129.7, 130.4, 133.0, 136.7, 139.3, 146.3, 149.1, 156.9, 157.6, 183.2, 195.4; HPLC, RT= 1.25 min; MS (ESI^+^): *m/z* 395.1 [M + H]^+^; HRMS calcd for C_24_H_19_N_4_O_2_ 395.1508, found 395.1507.

##### (4S)-4-methyl-2-benzofuran-3-yl-3,4-dihydro-5H-pyrido[1’,2’:1,2]imidazo[4,5-d][1,3]diazepin-5-one (7)

Yellow solid (21 mg, yield 19%); mp: 164–165 °C; [α]_D_^20^ = +23.2° (*c* 1.0, CHCl_3_); ^1^H NMR (CDCl_3_, 400 MHz): δ ppm 1.58 (d, 3H, *J* = 6.8 Hz), 3.46 (s, 1H), 4.17 (d, 1H, *J* = 6.8 Hz), 7.00 (dd, 1H, *J* = 6.8 Hz, 6.0 Hz), 7.26 (dd, 1H, *J* = 6.6 Hz, 7.7 Hz), 7.36 (t, 1H, J = 7.2 Hz), 7.49 (m, 2H), 7.63 (d, 2H, *J* = 7.6 Hz), 7.73 (s, 1H), 9.52 (d, 1H, *J* = 6.8 Hz); 13 C NMR (CDCl_3_, 100 MHz): δ ppm 16.6, 58.7, 111.1, 111.8, 114.1, 116.5, 121.2, 122.9, 124.1, 124.4, 127.0, 128.4, 128.7, 130.6, 146.7, 150.0, 155.4, 182.7; HPLC, RT= 1.17 min; MS (ESI^+^): *m/z* 331.0 [M + H]^+^; HRMS calcd for C_19_H_15_N_4_O_2_ 331.1195, found 331.1199.

##### (4S)-4-methyl-2-benzothiophen-3-yl-3,4-dihydro-5H-pyrido[1’,2’:1,2]imidazo[4,5-d][1,3]diazepin-5-one (8)

Brown solid (175 mg, yield 31%); mp: 135–137 °C; [α]_D_^20^ = +6.5° (*c* 1.0, CHCl_3_); ^1^H NMR (DMSO-d_6_, 500 MHz): δ ppm 1.39 (br, 3H), 4.07 (q, 1H, *J* = 6.1 Hz), 7.19 (td, 1H, *J* = 1.4 Hz, *J* = 6.8 Hz), 7.47 (t, 1H, *J* = 7.7 Hz), 7.53 (t, 1H, *J* = 7.3 Hz), 7.67 (td, 1H, *J* = 1.3 Hz, *J* = 6.8 Hz), 7.71 (d, 1H, *J* = 8.5 Hz), 8.07 (d, 1H, *J* = 7.9 Hz), 8.52 (br, 1H), 8.81 (br, 1H), 9.50 (br, 1H), 11.21 (br, 1H); 13 C NMR (DMSO-d_6_, 500 MHz): δ ppm 15.0, 56.6, 113.3, 115.6, 122.5, 124.6, 124.7, 125.0, 127.5, 130.0, 133.4, 137.0, 139.7, 146.1, 146.5, 148.0, 153.6, 157.6, 183.3; HPLC, RT= 1.98 min; MS (ESI^+^): *m/z* 347 [M + H]^+^; HRMS calcd for C_19_H_15_N_4_O_2_ 347.0975, found 347.0961.

##### (4S)-4-methyl-2-(1-boc-indol-3-yl)-3,4-dihydro-5H-pyrido[1’,2’:1,2]imidazo[4,5-d][1,3]diazepin-5-one (9)

*Yellow solid (*70 mg, yield 16%); mp: 155–157 °C; [α]_D_^20^ = −16.4° (*c* 0.8, CHCl_3_); ^1^H NMR (DMSO-d_6_, 400 MHz): δ ppm 1.38 (d, 3H, *J* = 6.5 Hz), 1.68 (s, 9H), 4.00 (q, 1H, *J* = 6.5 Hz), 7.18 (td, 1H, *J* = 1.4 Hz, *J* = 6.8 Hz), 7.36–7.44 (m, 2H), 7.66 (td, 1H, *J* = 1.2 Hz, *J* = 6.7 Hz), 7.71 (d, 1H, *J* = 8.7 Hz), 8.13 (d, 1H, *J* = 7.5 Hz), 8.49–8.69 (m, 3H), 9,47 (br, 1H); 13 C NMR (DMSO-d_6_, 400 MHz): δ ppm 15.3, 27.6, 56.8, 84.9, 113.6, 114.6, 115.7, 122.9, 123.4, 125.1, 127.7, 128.1, 129.5, 130.4, 133.6, 134.1, 135.2, 136.9, 146.2, 148.7, 157.8, 195.5; HPLC, RT= 2.60 min; MS (ESI^+^): *m/z* 430.1 [M + H]^+^; HRMS calcd for C_24_H_24_N_5_O_3_ 430.1879, found 430.1886.

##### (4S)-4-methyl-2-quinolin-6-yl-3,4-dihydro-5H-pyrido[1’,2’:1,2]imidazo[4,5-d][1,3]diazepin-5-one (10)

Brown solid (9 mg, yield 8%); mp: 165–166 °C; [α]_D_^20^ = +52.4° (*c* 0.25, CHCl_3_); ^1^H NMR (CDCl_3_, 500 MHz): 2 conformers δ ppm 1.54 (d, 1.5H, *J* = 6.1 Hz), 1.74 (d, 1.5H, *J* = 6.1 Hz), 4.19 (q, 1H, *J* = 6.1 Hz), 6.95 (m, 2H), 7.37 (m, 1H), 7.49 (dd, 0.5H, *J* = 6.7 Hz, 7.9 Hz), 7.65 (d, 0.5H, *J* = 7.7 Hz), 8.06–8.16 (m, 2H), 8.27 (d, 1H, *J* = 8.6 Hz), 8.35 (br, 0.5 H), 8.57 (br, 0.5H), 8.89 (d, 1H, *J* = 2.9 Hz), 9.43 (d, 0.5H, *J* = 5.4 Hz), 9.52 (d, 0.5H, *J* = 5.4 Hz), 10.52 (br, 1H); 13 C NMR (CDCl_3_, 100 MHz): 2 conformers δ ppm 16.3, 17.0, 64.0, 113.9, 115.0, 116.8, 122.1, 127.8, 128.6, 129.0, 129.7, 129.9, 130.0, 130.2, 130.3, 130.5, 133.0, 133.7, 136.9, 137.3, 145.4, 147.2, 149.4, 149.6, 152.1, 152.2, 152.7, 153.0, 157.8, 158.2, 182.4, 183.3; HPLC, RT= 0.92 min; MS (ESI^+^): *m/z* 342.2 [M + H]^+^; HRMS calcd for C_20_H_16_N_5_O 342.1355, found 342.1356.

##### (4S)-4-methyl-2-quinolin-2-yl-3,4-dihydro-5H-pyrido[1’,2’:1,2]imidazo[4,5-d][1,3]diazepin-5-one (11)

Yellow solid (92 mg, yield 82%); mp: 170–171 °C; [α]_D_^20^ = +22.5° (*c* 0.6, CHCl_3_); ^1^H NMR (CDCl_3_, 400 MHz): (2 conformers) δ ppm 1.56 (d, 2H, *J* = 6.6 Hz), 1.68 (d, 1H, *J* = 4.9 Hz), 4.26 (m, 0.7H), 4.36 (m, 0.3H), 7.00 (dd, 1H, *J* = 7.0 Hz, 6.7 Hz),7.49 (ddd, 1H, *J* = 8.8 Hz, 8.3 Hz, 1.2 Hz), 7.58 (dd, 1H, *J* = 7.2 Hz, 7.6 Hz), 7.67 (d, 1H, *J* = 8.3 Hz), 7.74 (ddd, 1H, *J* = 7.0 Hz, 7.0 Hz, 1.2 Hz), 7.86 (d, 1H, *J* = 7.3 Hz), 8.21–8.30 (m, 2H), 8.66 (br, 1H), 8.88 (s, 1H, *J* = 8.3 Hz), 9.52 (d, 0.3H, *J* = 6.2 Hz), 9.60 (d, 0.7H, *J* = 6.2 Hz); 13 C NMR (CDCl_3_, 100 MHz): δ ppm 17.1, 57.2, 113.8, 116.6, 119.7, 127.9, 128.0, 128.8, 129.4, 129.7, 129.9, 130.2, 130.3, 132.4, 137.4, 146.5, 150.9, 152.2, 163.0, 183.0; HPLC, RT= 1.36 min; MS (ESI^+^): *m/z* 342.2 [M + H]^+^; HRMS calcd for C_20_H_16_N_5_O 342.1355, found 342.1356.

##### (4S)-4-methyl-2-coumarin-6-yl-3,4-dihydro-5H-pyrido[1’,2’:1,2]imidazo[4,5-d][1,3]diazepin-5-one (12)

Yellow solid (46 mg, yield 39%); mp: 132.5–133.5 °C; [α]_D_^20^ = −8.4° (*c* 0.6, DMSO); ^1^H NMR (DMSO d_6_, 500 MHz): δ ppm 1.42 (d, 3H, *J* = 6.7 Hz), 4.08 (q, 1H, *J* = 6.7 Hz), 6.60 (d, 1H, *J* = 9.7 Hz), 7.23 (td, 1H, *J* = 6.5 Hz, 2.0 Hz), 7.56 (d, 1H, *J* = 8.7 Hz), 7.71 (m, 2H), 8.20 (m, 2H), 8.36 (s, 1H), 9.47 (d, 1H, *J* = 6.5 Hz); 13 C NMR (DMSO d_6_, 125 MHz): δ ppm 15.4, 58.0, 112.3, 113.0, 114.3, 115.7, 116.5, 117.1, 118.5, 127.9, 129.6, 130.1, 130.6, 131.0, 132.9, 144.1, 145.5, 155.8, 159.6, 182.3; HPLC, RT= 0.99 min; MS (ESI^+^): *m/z* 359.1 [M + H]^+^; HRMS calcd for C_20_H_15_N_4_O_3_ 359.1144, found 359.1147.

##### (4S)-4-butyl-(4-bromophenyl)-3,4-dihydro-5H-pyrido[1’,2’:1,2]imidazo[4,5-d][1,3]diazepin-5-one (13)

Brown gum (72 mg, yield 53%); [α]_D_^20^ = +89.1° (*c* 0.6, MeOH); ^1^H NMR (CDCl_3_, 400 MHz): δ ppm 0.80 (t, 3H, *J* = 7.2 Hz), 1.21–1.37 (m, 4H), 1.65 (m, 2H), 4.08 (t, 1H, *J* = 7.2 Hz), 7.34 (t, 1H, *J* = 6.8 Hz), 7.37 (d, 2H, *J* = 8.6 Hz), 7.82 (m, 3H), 7.91 (m, 2H), 9.57 (d, 1H, *J* = 6.8 Hz); 13 C NMR (CDCl_3_, 100 MHz): δ ppm 13.9, 22.2, 27.9, 30.0, 63.4, 111.4, 113.7, 117.3, 129.2, 129.3, 130.8, 131.4, 132.2, 134.6, 141.2, 149.0, 160.6, 181.8; HPLC, RT= 1.35 min; MS (ESI^+^): *m/z* 411.1 [M + H]^+^, 413.1 [M + 2 + H]^+^; HRMS calcd for C_20_H_20_N_4_O^79^Br 411.0820, found 411.0821.

##### (4S)-4-propargyl-2-(4-bromophenyl)-3,4-dihydro-5H-pyrido[1’,2’:1,2]imidazo[4,5-d][1,3]diazepin-5-one (as trifluoroacetate salt) (14)

Pale yellow solid (8 mg, yield 5%); [α]_D_^20^ = +17.0° (*c* 0.1, CHCl_3_); ^1^H NMR (CD_3_OD, 500 MHz): δ ppm 2.60 (t, 1H, *J* = 2.3 Hz), 2.69 (m, 1H), 2.80 (m, 1H), 4.16 (dd, 1H, *J* = 4.5 Hz, 4.4 Hz), 7.24 (td, 1H, *J* = 6.8 Hz, 1.0 Hz), 7.70–7.76 (m, 4H), 8.04 (d, 2H, *J* = 8.3 Hz), 9.57 (d, 1H, *J* = 6.8 Hz); 13 C NMR (CD_3_OD, 125 MHz): δ ppm 24.9, 67.6, 72.4, 83.3, 121.8, 122.5, 124.8 (d, *J* = 284 Hz, TFA), 125.2, 126.6, 128.0, 137.4, 139.6, 140.1, 140.5, 142.4, 153.6, 155.0, 165.0 (TFA), 191.9; HPLC, RT= 1.23 min; MS (ESI^+^): *m/z* 393.0 [M + H]^+^, 395.0 [M + 2 + H]^+^; HRMS calcd for C_19_H_14_N_4_O^79^Br 393.0351, found 393.0352.

##### (4S)-4-isobutyl-2-(4-bromophenyl)-3,4-dihydro-5H-pyrido[1’,2’:1,2]imidazo[4,5-d][1,3]diazepin-5-one (15)

Yellow solid (133 mg, yield 98%); mp: 72–73 °C; [α]_D_^20^ = +33.0° (*c* 0.4, CHCl_3_); ^1^H NMR (CDCl_3_, 400 MHz): δ ppm 0.94 (d, 6H, *J* = 6.4 Hz), 1.65–2.07 (m, 3H), 4.07 (m, 1H), 6.98 (td, 1H, *J* = 6.7 Hz, 1.0 Hz), 7.42 (m, 1H), 7.52 (d, 2H, *J* = 8.4 Hz), 7.63 (m, 1H), 7.86 (br, 2H), 9.50 (d, 1H, *J* = 6.7 Hz), 10.63 (br, 1H); 13 C NMR (CDCl_3_, 100 MHz): δ ppm 21.9, 25.0, 39.3, 60.8, 113.9, 115.0, 116.9, 127.0, 128.6, 130.3, 130.6, 132.1, 132.4, 134.9 (4 C), 156.8, 158.0, 186.1; HPLC, RT= 1.34 min; MS (ESI^+^): *m/z* 411.1 [M + H]^+^, 413.1 [M + 2 + H]^+^; HRMS calcd for C_20_H_20_N_4_O^79^Br 411.0820, found 411.0821.

##### (4S)-4-(4-azidobutyl)-2-(4-bromophenyl)-3,4-dihydro-5H-pyrido[1’,2’:1,2]imidazo[4,5-d][1,3]diazepin-5-one (16)

Yellow solid (45 mg, yield 30%); mp: 127–128 °C; [α]_D_^20^ = +39.7° (*c* 0.4, CHCl_3_); ^1^H NMR (CDCl_3_, 400 MHz): δ ppm 1.57–1.70 (m, 6H), 3.31 (m, 2H), 3.93 (m, 1H), 7.00 (dd, 1H, *J* = 6.2 Hz, 6.8 Hz), 7.45 (m, 2H), 7.54 (d, 2H, *J* = 8.4 Hz), 7.85 (br d, 2H), 9.48 (d, 1H, *J* = 6.2 Hz), 10.41 (br, 1H); 13 C NMR (CDCl_3_, 100 MHz): δ ppm 23.7, 29.0, 29.9, 30.3, 51.5, 114.0, 115.4, 116.2, 122.0, 128.7, 130.0, 130.6, 132.2, 134.1, 139.8, 147.2, 153.7, 182.9; HPLC, RT= 1.37 min; MS (ESI^+^): *m/z* 452.0 [M + H]^+^, 454.0 [M + 2 + H]^+^; HRMS calcd for C_20_H_19_N_7_O^79^Br 452.0834, found 452.0835.

##### (4R)-4-(4-azidobutyl)-2-(4-bromophenyl)-3,4-dihydro-5H-pyrido[1’,2’:1,2]imidazo[4,5-d][1,3]diazepin-5-one (17)

Yellow solid (45 mg, yield 30%); [α]_D_^20^ = −39.1° (*c* 0.3, CHCl_3_); ^1^H NMR (CDCl_3_, 400 MHz): δ ppm 1.57–1.70 (m, 6H), 3.31 (m, 2H), 3.93 (m, 1H), 7.00 (dd, 1H, *J* = 6.2 Hz, 6.8 Hz), 7.45 (m, 2H), 7.54 (d, 2H, *J* = 8.4 Hz), 7.85 (br d, 2H), 9.48 (d, 1H, *J* = 6.2 Hz), 10.41 (br, 1H); 13 C NMR (CDCl_3_, 100 MHz): δ ppm 23.7, 29.0, 29.9, 30.3, 51.5, 114.0, 115.4, 116.2, 122.0, 128.7, 130.0, 130.6, 132.2, 134.1, 139.8, 147.2, 153.7, 182.9; HPLC, RT= 1.37 min; MS (ESI^+^): *m/z* 452.0 [M + H]^+^, 454.0 [M + 2 + H]^+^; HRMS calcd for C_20_H_19_N_7_O^79^Br 452.0834, found 452.0835.

##### (4S)-4-(4-azidobutyl)-2-phenyl-3,4-dihydro-5H-pyrido[1’,2’:1,2]imidazo[4,5-d][1,3]diazepin-5-one (18)

Yellow solid (45 mg, yield 30%); mp: 116–117 °C; [α]_D_^20^ = +20.0° (*c* 0.5, CHCl_3_); ^1^H NMR (CDCl_3_, 400 MHz): δ ppm 1.57–1.71 (m, 4H), 2.05 (m, 2H), 3.32 (t, 2H, *J* = 6.4 Hz), 3.93 (m, 1H), 6.97 (dd, 1H, *J* = 7.5 Hz, 6.6 Hz), 7.38 (m, 1H), 7.43 (dd, 1H, *J* = 7.8 Hz, 6.6 Hz), 7.49 (d, 2H, *J* = 7.2 Hz), 7.98 (d, 1H, J = 6.7 Hz), 9.48 (d, 1H, *J* = 6.6 Hz), 10.60 (br, 1H); 13 C NMR (CDCl_3_, 100 MHz): δ ppm 23.6, 28.9, 29.7, 30.1, 51.4, 113.6, 115.5, 128.4, 128.8, 129.0, 129.7, 130.2, 131.7, 135.1, 139.7, 145.7, 147.0, 182.0; HPLC, RT= 0.97 min; MS (ESI^+^): *m/z* 373.9 [M + H]^+^; HRMS calcd for C_20_H_20_N_7_O 374.1729, found 374.1731.

##### (4S)- benzyl [2-(4-bromophenyl)-5-oxo-4,5-dihydro-3H-pyrido[1’,2’:1,2]imidazo[4,5-d][1,3]diazepin-4-yl]acetate (19)

Orange solid (22 mg, yield 13%); mp: 72.5–73.5 °C; [α]_D_^20^ = +10° (*c* 0.1, CHCl_3_); ^1^H NMR (CDCl_3_, 400 MHz): δ ppm 3.17 (dd, 1H, *J* = 16.3 Hz, 7.6 Hz), 3.44 (dd, 1H, *J* = 16.3 Hz, 6.2 Hz), 4.50 (dd, 1H, *J* = 7.6 Hz, 6.2 Hz), 5.24(m, 2H), 6.49 (d, 1H, *J* = 8.9 Hz), 6.98 (t, 1H, *J* = 6.6 Hz), 7.26–7.34 (m, 6H), 7.39 (d, 2H, *J* = 6.6 Hz), 7.47 (d, 2H, *J* = 8.4 Hz), 7.70 (d, 2H, *J* = 8.4 Hz), 9.42 (d, 1H, *J* = 6.7 Hz); 13 C NMR (CDCl_3_, 100 MHz): δ ppm 36.7, 64.9, 66.5, 111.7, 114.0, 114.9, 126.4, 127.1, 128.3, 128.4, 128.7, 130.7, 131.1, 132.1, 133.7, 136.4, 145.4, 152.9, 153.7, 172.4, 180.0; HPLC, RT= 1.60 min; MS (ESI^+^): *m/z* 503.1 [M + H]^+^, 505.1 [M + 2 + H]^+^; HRMS calcd for C_25_H_20_N_4_O_3_^79^Br 503.0719, found 503.0720.

##### 7–(4-bromophenyl)-9,10,11,11a-tetrahydro-12H-pyrido[2’,1’:2,3]imidazo[4,5-f]pyrrolo[1,2-c][1,3]diazepin-12-one (20)

Brown gum (128 mg, yield 98%); [α]_D_^20^ = −75.5° (*c* 1.0, MeOH); ^1^H NMR (CDCl_3_, 400 MHz): δ ppm 1.77–1.87 (m, 1H), 1.98–2.14 (m, 2H), 3.15 (m, 1H), 3.30 (td, 1H, *J* = 7.3 Hz, 1.2 Hz), 3.85 (ddd, 1H, *J* = 6.4 Hz, 6.4 Hz, 6.4 Hz), 4.08 (dd, 1H, *J* = 6.4 Hz, 1.5 Hz), 6.96 (td, 1H, *J* = 6.8 Hz, 1.3 Hz), 7.46 (ddd, 1H, *J* = 8.9 Hz, 6.8 Hz, 1.3 Hz), 7.55 (s, 4H), 7.63 (d, 1H, *J* = 8.9 Hz), 9.50 (d, 1H, *J* = 6.8 Hz); 13 C NMR (CDCl_3_, 100 MHz): δ ppm 24.7, 25.6, 53.4, 64.9, 113.6, 114.7, 116.9, 125.4, 128.4, 130.1, 131.6 (2 C), 131.8 (2 C), 135.3, 146.9, 158.8, 159.9, 179.8; HPLC, RT= 1.19 min; MS (ESI^+^): *m/z* 395.0 [M + H]^+^, 397.0 [M + 2 + H]^+^; HRMS calcd for C_19_H_16_N_4_O^79^Br 395.0507, found 395.0510.

##### Synthesis of (4S)-4-methyl-2–(4-ethynylphenyl)-3,4-dihydro-5H-pyrido[1’,2’:1,2]imidazo[4,5-d][1,3]diazepin-5-one (4’)

To a solution of 70 mg of compound **4** (0.18 mmol) in 2 ml of THF, 181 µL of a solution of 2 M tetrabutylammonium fluoride in THF were added. The solution was stirred at room temperature for 2 h until completion of the deprotection (HPLC analysis). The solution was then evaporated to dryness *in vacuo*. The residue was dissolved in ethyl acetate and washed with an aqueous saturated solution of ammonium chloride. The organic layer was dried over Na_2_SO_4_, filtered and the solvent was evaporated *in vacuo* to offer compound **4′** as a yellow solid (56 mg, yield qt). Mp: 128–129 °C; [α]_D_^20^ = +20.4° (*c* 0.5, CHCl_3_); ^1^H NMR (CDCl_3_, 300 MHz): δ ppm 1.63 (br, 3H), 3.17 (s, 1H), 4.10 (q, 1H, *J* = 6.7 Hz), 6.57 (br, 1H), 6.97 (dd, 1H, *J* = 7.6 Hz, 6.6 Hz), 7.37 (m, 1H), 7.50 (d, 2H, *J* = 8.3 Hz), 7.94 (d, 2H, *J* = 8.3 Hz), 9.49 (d, 1H, *J* = 6.6 Hz); 13 C NMR (CDCl_3_, 75 MHz): δ ppm 16.7, 66.0, 79.9, 82.9, 113.7, 113.9, 125.6, 128.5 (2 C), 128.6, 129.2, 130.4, 132.4, 132.5 (2 C), 135.4, 153.3, 152.3, 180.5; HPLC, RT= 1.07 min; MS (ESI^+^): *m/z* 315.1 [M + H]^+^; HRMS calcd for C_19_H_15_N_4_O 315.1246, found 315.1248.

##### Synthesis of compound (4S)-4-methyl-2-indol-3-yl-3,4-dihydro-5H-pyrido[1’,2’:1,2]imidazo[4,5-d][1,3]diazepin-5-one (9’)

A solution of 50 mg of compound **9** (0.12 mmol) in a 50/50 v/v mixture of trifluoroacetic acid and dichloromethane (5 ml) was stirred at room temperature. After completion of the reaction (HPLC analysis), the solution was concentrated to dryness and co-evaporated several times with acetonitrile to completely remove TFA. Then, 5 ml of water were added and the solution was basified by K_2_CO_3_ and then extracted by DCM (3 × 10 ml). The organic layer was dried over Na_2_SO_4_, filtered, and the solvent was evaporated *in vacuo* to offer compound **9′** as a white solid *(*33 mg, yield 83%); mp: 164.5–165.5 °C; [α]_D_^20^ = +12.1° (*c* 1.0, MeOH); ^1^H NMR (DMSO-d_6_, 500 MHz): δ ppm 1.31 (d, 3H, *J* = 7.0 Hz), 3.98 (q, 1H, *J* = 5.6 Hz), 7.11 (t, 1H, *J* = 6.5 Hz), 7.16–7.20 (m, 2H), 7.47 (d, 1H, *J* = 6.8 Hz), 7.58–7.65 (m, 2H), 8.25 (d, 1H, *J* = 4.6 Hz), 8.30 (s, 1H), 8.62 (d, 1H, *J* = 6.5 Hz), 9.51 (d, 1H, *J* = 6.7 Hz), 11.96 (s, 1H); 13 C NMR (DMSO-d_6_, 500 MHz): δ ppm 15.0, 56.4, 111.7, 112.2, 112.7, 115.4, 120.2, 121.9, 122.1, 126.1, 127.5, 128.6, 129.7, 130.2, 136.7, 146.4, 155.9, 158.7, 184.3; HPLC, RT= 1.81 min; MS (ESI^+^): *m/z* 330 [M + H]^+^; HRMS calcd for C_19_H_16_N_5_O 330.1363, found 330.1349.

##### Synthesis of compound (4S)-[2-(4-bromophenyl)-5-oxo-4,5-dihydro-3H-pyrido[1’,2’:1,2]imidazo[4,5-d][1,3]diazepin-4-yl]acetic acid (21)

To a solution of compound **19** (20 mg, 0.04 mmol) in 5 ml of DCM was added at 0 °C a mixture of HBr 33% in acetic acid (5 ml). The solution was then stirred at room temperature. After completion of the reaction (HPLC analysis), the solution was concentrated to dryness and the crude product was dissolved in 2 ml of DMSO and purified by preparative HPLC (gradient of 100% H_2_O/0.1% TFA to 40% CH_3_CN/0.1% TFA). The fractions containing the product were collected. The ACN was evaporated *in vacuo*. The aqueous residue was basified by NaHCO_3_ and extracted with DCM (3 × 25 ml). The organic layer was dried over Na_2_SO_4_, filtered, and the solvent was evaporated *in vacuo* to offer compound **21** as a white solid (5 mg, yield 12%); [α]_D_^20^ = −57,2° (*c* 0.25, DMSO); ^1^H NMR (DMSO-d_6_, 500 MHz): δ ppm 2.90 (dd, 1H, *J* = 8.2 Hz, *J* = 16.9 Hz), 3.05 (dd, 1H, *J* = 5.6 Hz, *J* = 16.7 Hz), 4.38 (t, 1H, *J* = 5.5 Hz), 7.37 (t, 1H, *J* = 6.0 Hz), 7.83–7.89 (m, 6H), 9.45 (d, 1H, *J* = 6.7 Hz), 12.71 (s, 1H); 13 C NMR (DMSO-d_6_, 500 MHz): δ ppm 33.5, 59.8, 112.0, 115.1, 115.5, 117.9, 127.2, 128.2, 132.3, 132.5, 144.0, 152.2, 157.8, 158.1, 171.9, 179.3; HPLC, RT= 1.85 min; MS (ESI^+^): *m/z* 413 [M + H]^+^; HRMS calcd for C_18_H_14_N_4_O_3_^79^Br 413.0244, found 413.0257.

##### Synthesis of (4S)-N-{4-[2-(4-bromophenyl)-5-oxo-4,5-dihydro-3H-pyrido[1’,2’:1,2]imidazo[4,5-d][1,3]diazepin-4-yl]butyl}pent-4-ynamide (22)

To a solution of compound **23** as a bromhydrate salt^11^ (300 mg, 0.6 mmol) in 15 ml of DCM, hydroxybenzotriazole monohydrate (101 mg, 0.66 mmol, 1.1 equiv.), EDCI (126 mg, 0.66 mmol, 1.1 equiv.), 4-pentynoic acid (59 mg, 0.6 mmol, 1 equiv.) and triethylamine (184 µL, 1.3 mmol, 2.2 equiv.) were added at 0 °C. The solution was stirred at 0 °C for 30 min and then at room temperature for one night. The solution was washed with an aqueous saturated NaHCO_3_ solution and then with brine. The organic layer was dried over Na_2_SO_4_, filtered and the solvent was evaporated *in vacuo*. The residue was purified by chromatography on alumina, eluted by DCM/EtOH 99/1 v/v to offer compound **22** as a brown gum (280 mg, yield 94%); two conformers detected by NMR in CDCl_3_; ^1^H NMR (CDCl_3_, 500 MHz): δ ppm 1.44–1.77 (m, 6H), 2.30–2.51 (m, 4H), 3.21 (m, 1H), 3.35 (m, 1H), 3.85–4.06 (m, 1H), 6.05 (t, 1H, *J* = 5.1 Hz), 6.60 (d, 1H, *J* = 8.7 Hz), 6.98 (t, 1H, *J* = 6.9 Hz), 7.29–7.37 (m, 1H), 7.52 (d, 2H, *J* = 8.4 Hz), 7.78 (d, 1H, *J* = 7.8 Hz), 7.96 (d, 1H, *J* = 7.5 Hz), 9.45 (m, 1H), 10.91 (br, 1H); 13 C NMR (CDCl_3_, 125 MHz): δ ppm 15.1, 23.1, 29.6, 30.3, 35.6, 39.5, 67.9, 69.6, 83.2, 114.0, 115.0, 116.7, 126.9, 128.7, 130.5, 130.8, 132.1, 134.7, 147.3, 153.3, 158.2, 171.6, 183.1; HPLC, RT= 1.21 min; MS (ESI^+^): *m/z* 506.1 [M + H]^+^, 508.2 [M + 2 + H]^+^; HRMS calcd for C_25_H_25_N_5_O_2_^79^Br 506.1192, found 506.1193.

##### General procedure for diazo transfer

To a solution of compound **23** as a bromhydrate salt (0.24 mmol) in 6 ml of methanol, were added K_2_CO_3_ (110 mg, 0.8 mmol, 3.3 equiv.), copper II sulfate pentahydrate (0.5 mg, 0.002 mmol, 0.01 equiv) and imidazole-1-sulfonyl azide hydrochloride (0.26 mmol, 1.1 equiv.). The solution was stirred at room temperature for 24 h. The solution was then evaporated *in vacuo*. 5 ml of water were added and the solution was extracted with ethyl acetate (3 × 15 ml). The organic layer was dried over Na_2_SO_4_, filtered, and the solvent was evaporated *in vacuo*. The residue was purified by chromatography on alumina, eluted by nHex/AcOEt 60/40 v/v and then 50/50 v/v to offer azido compound **16** as a pale yellow powder (35 mg, yield 32%). Spectral characteristics are in agreement with those described below.

### Biological evaluation

#### Cell lines and culture

Melanoma human cancer cell line (A375 and MDA-MB-435) were obtained from ATCC. The NIH-3T3 cell line was generously gifted by the ANSM (French National Agency for Medicines and Health Products Safety, Vendargues, France). Cells were cultured in Dulbecco’s Modified Eagle Medium (DMEM, Sigma Aldrich) supplemented with 10% heat-inactivated foetal calf serum (FCS, Gibco), 2 mM glutamax (Gibco), 100 IU/mL penicillin G sodium (Sigma Aldrich), 100 lU/mL streptomycin sulphate (Sigma Aldrich). The cells were allowed to grow at 37 °C in a fully humidified atmosphere containing 5% CO_2_.

#### Neutral red uptake (NRU) viability assay

A viability assay was used to evaluate the cytotoxic activity on A375 and MDA-MB-435 melanoma cells as previously described^20^. According to OCDE/ICCVAM guidance n°129, NRU assay was also used to evaluate the toxicity on the non-cancerous NIH-3T3 cells^21^. Cells were seeded at a final concentration of 2000 cells/well (A375), 3000 cells/well (NIH-3T3) or 7500 cells/well (MDA-MB-435) in 96-well microtiter plates for 24 h. Then, cells were exposed to two negative controls (1% DMSO in culture medium (vehicle) and culture medium alone), or to different compounds freshly dissolved in DMSO, in a range of concentration from 0.01 to 40 µM (six wells per concentration). After 72 h of incubation, cells were stained with new medium containing 50 µg/mL of neutral red (3-amino-7-dimethylamino-2-methylphenazine hydrochloride, Sigma Aldrich) at 37 °C for 2 h. The neutral red medium solution was then removed and the wells were washed with PBS. Finally, 100 µL of solution containing ethanol, acetic acid and water (50/1/49 v/v/v respectively) were added into the wells and the absorbance was measured at 540 nm using the microplate reader 352 Multiscan MS (Labsystems). Cell survival rates were normalised to the absorbance values of untreated control cells. IC_50_ was obtained using a logistic regression model on Graphpad Prism 5.0 software. Results are given as the mean ± standard deviation (SD) of three independent experiments. Statistical analysis was performed using the Student’s t-test.

#### Sulforhodamine B (SRB) assay

Primary anticancer assay was performed on a panel of 60 human cancer cell lines derived from nine neoplastic diseases, in accordance with the protocol of the Drug Evaluation Branch, National Cancer Institute, Bethesda, Maryland, USA^22^. Cells were incubated with tested compounds at 10 µM for 48 h. The growth percentage was evaluated spectrophotometrically using a protein binding dye, SRB. Results for each tested compound were reported as the percent of growth of treated cells when compared to untreated control cells.

#### Cell cycle analysis

The cell cycle analysis was performed as previously described to compare data with our previous results^10^. Flow cytometric analysis was performed on 350,000 MDA-MB-435 cells seeded in culture dishes (60 mm diameter) and allowed to grow for 48 h. Cells were then treated with compound **5** or **8** at 5 µM concentration for 16 h and 48 h. After treatment cells were harvested and fixed with 70% ethanol. The fixed cells were then incubated with 10 mg/mL RNase A (Sigma Aldrich) and 1 mg/mL propidium iodide (Sigma Aldrich), in the dark, for 24 h at 4 °C. Finally, DNA content of the cells was analysed using Gallios Beckman Coulter flow cytometer with FlowJo software v1.0.

#### Immunofluorescent staining for actin localisation

To evaluate the effect of compound treatment on cell morphology and stress actin fibres, an immunofluorescence imaging of cells was performed as previously described^10^. MDA-MB-435 cells were seeded on cover slips in petri dishes and allowed to grow for 24 h. After cell growth, cells were treated with the vehicule (control cells) or with JMV5038, compound **5** or **8** at 5 µM for 20 h. Then cells were fixed using Antigenfix for 20 min and permeabilized using 0.2% Triton X-100 for 4 min at room temperature. Actin was stained using a primary human anti-actin antibody (made from mouse) and a secondary Alexa Fluor 568-conjugated goat anti-mouse antibody. Both of them were incubated 1 h at room temperature. Nuclei were counter-stained using Hoechst 33342. Representative images were obtained using a Zeiss LSM 780 inverted confocal microscopy. Confocal fluorescence microscopy was performed on fixed cells under a 543 nm wavelength excitation for AlexaFluor568, and 405 nm for nuclei, and visualised at 620 nm (red) and 450 nm (blue) respectively.

#### Statistical analysis

**Figure 2.**
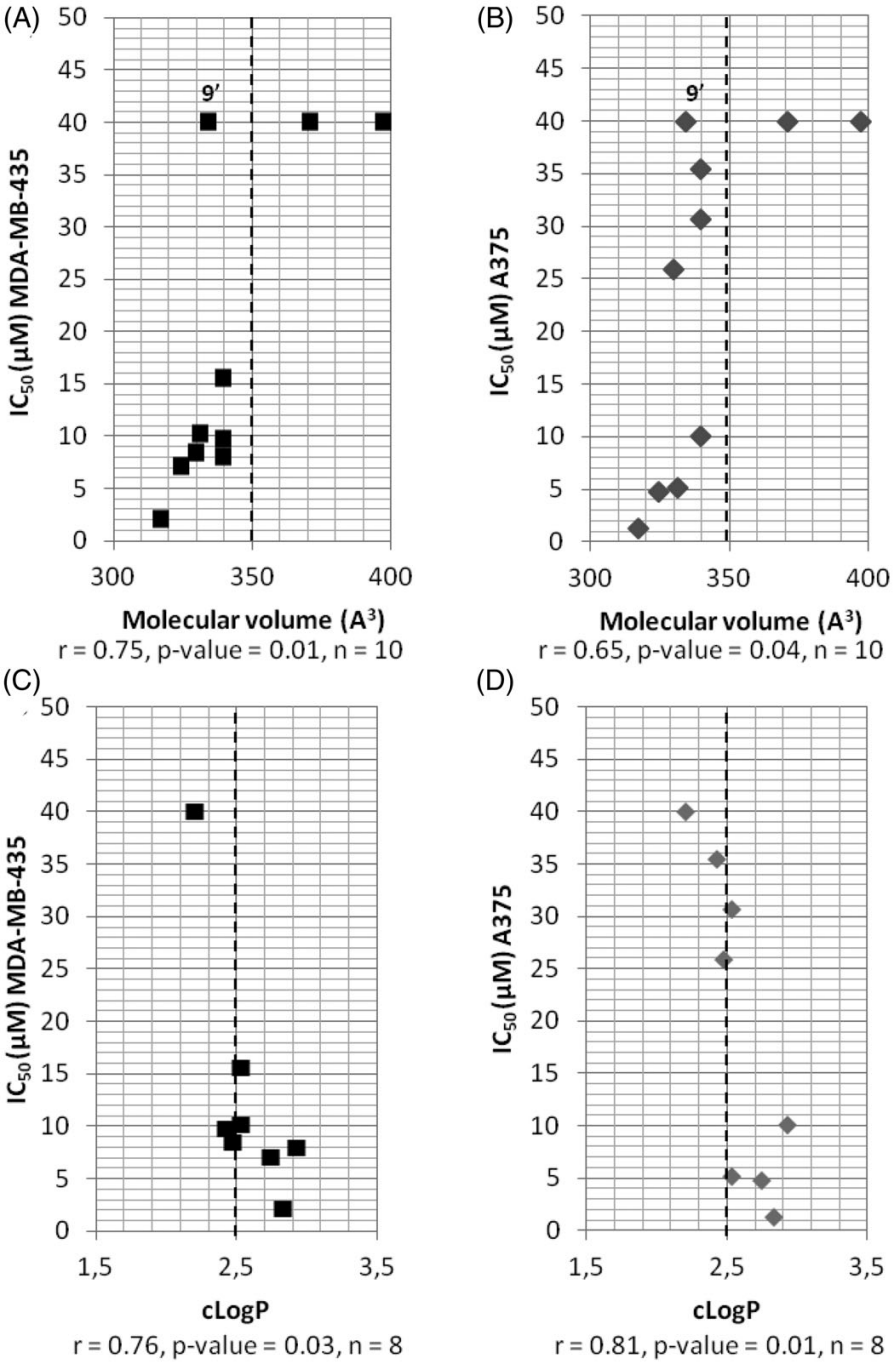
Correlations between IC_50_, determined in two melanoma cell lines, and molecular volume of compounds modified in position *R*^2^ (A and B) or between IC_50_ and *c*LogP (C and D). ▪: A375 cell line; ◆: MDA-MB-435 cell line; r: Pearson’s correlation coefficient, *p* values: level of significance, *n*: sample size; the dotted line separates the most active compounds (IC_50_ < 15 µM) from the less active ones (IC_50_ > 15 µM), except for compound **9’** (see the text).

Data set of physicochemical properties (*c*LogP, TPSA and molecular volume, Table 2) were calculated using the online available molsoft cheminformatic tool^23^. IC_50_ values of the compounds included in the test are presented in Table 1 for the correlations between IC_50_ and physicochemical properties of compounds modified in position 2 (Figure 2), and in Table S1 (SI) for the correlations between *in vitro* anti-cancer activity of JMV5038, compound **5** and compound **7** on the NCI cell line panel (Figure 4). Pearson’s correlation coefficient r and level of significant *p* values were calculated using Origin Pro 2019.

**Table 1.** Structure and IC_50_ values of compounds on the A375, MDA-MB-435 and NIH-3T3 cell lines.
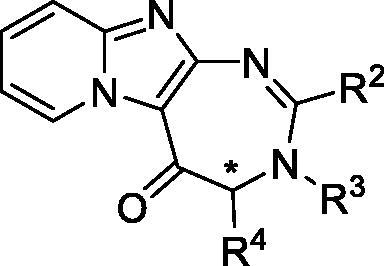

Entry	Compound	*R*^2^	*R*^3^	*R*^4^	IC_50_ A375 (µM)	IC_50_ MDA-MB-435 (µM)	IC_50_ NIH-3T3 (µM)
1	**JMV5038**	4-Br-C_6_H_4_	H	(*S*)-Me	2.10 ± 0.42	1.30 ± 0.53	>100
2	**4′**	4-HCC-C_6_H_4_	H	(*S*)-Me	10.23 ± 0.55	5.14 ± 0.08	>40
3	**5**	4-N_3_-C_6_H_4_	H	(*S*)-Me	7.13 ± 0.51	4.75 ± 0.21	>40
4	**6**	4-Bz-C_6_H_4_	H	(*S*)-Me	>40	>40	NT^a^
5	**7**	3-benzofuranyl	H	(*S*)-Me	8.47 ± 0.21	25.92 ± 0.17	>40
6	**8**	3-benzothiophenyl	H	(*S*)-Me	7.97 ± 0.65	10.12 ± 0.08	>40
7	**9′**	3-indolyl	H	(*S*)-Me	>40	>40	NT
8	**10**	6-quinolinyl	H	(*S*)-Me	9.76 ± 0.95	35.49 ± 0.21	>40
9	**11**	2-quinolinyl	H	(*S*)-Me	15.57 ± 0.7	30.71 ± 0.19	>40
10	**12**	6-coumarinyl	H	(*S*)-Me	>40	>40	NT
11	**13**	4-Br-C_6_H_4_	H	(*S*)-*n-*Bu	15.29 ± 1.29	30.83 ± 0.09	>40
12	**14**	4-Br-C_6_H_4_	H	(*S*)-CH_2_CCH	>40	>30	NT
13	**15**	4-Br-C_6_H_4_	H	(*S*)-CH_2_CH(CH_3_)_2_	>40	>30	NT
14	**16**	4-Br-C_6_H_4_	H	(*S*)-(CH_2_)_4_N_3_	3.68 ± 0.63	4.12 ± 0.22	9.90 ± 0.51
15	**17**	4-Br-C_6_H_4_	H	(*R*)-(CH_2_)_4_N_3_	8.54 ± 0.22	30.90 ± 0.11	13.71 ± 0.34
16	**18**	Ph	H	(*S*)-(CH_2_)_4_N_3_	>40	>40	NT
17	**19**	4-Br-C_6_H_4_	H	(*S*)-CH_2_COOBn	9.78 ± 0.83	19.44 ± 0.64	>40
18	**20**	4-Br-C_6_H_4_	(*S*)-[(CH_2_)_3_]_c_		>40	>40	NT
19	**21**	4-Br-C_6_H_4_	H	(*S*)-CH_2_COOH	>40	>40	NT
20	**22**	4-Br-C_6_H_4_	H	(*S*)-(CH_2_)_4_NHCO(CH_2_)_3_CCH	>40	>40	NT

^a^NT: not tested.

Bold values correspond to compounds number.

## Results and discussion

### Chemistry

Pyrido-imidazodiazepinones **4–20** were synthesised starting from 2-amino-imidazo[1,2-*a*]pyridine **1**, by selective *C*-3 acylation of the imidazo[1,2-*a*]pyridine nucleus (Scheme 1), using 9 different *N*-Boc amino-acids (Boc-Ala-OH, Boc-Nle-OH, Boc-Leu-OH, Boc-Asp(Bn)-OH, Boc-Pra-OH, *S* or *R* Boc-Lys(Cbz)-OH, Boc-6-azido-Nle-OH or Boc-Pro-OH), according to our previously reported methodology^10^^,^^11^^,^^13^^,^^14^. *C*-3 acylated compounds **2a–i** were isolated in 36–97% yield after chromatography on alumina gel. Only traces of the corresponding *N*-acylated derivatives were detected in the crude mixture by LC-MS analysis. After Boc removal using a mixture of trifluoroacetic acid/dichloromethane (50/50 v/v), the resulting 2-amino-3-acyl-imidazo[1,2-*a*] pyridines **3a-i** were isolated as trifluoroacetate salts, and used without further purification. Diamines **3a-i** were then successively reacted with a set of aldehydes, using two strategies. For compounds bearing an alkyne group (compounds **4** and **14**), a 3-step procedure was used. Firstly, the trifloroacetate salts of the diamines were neutralised and converted into their corresponding free bases. Secondly, these latter were then refluxed successively in chloroform with the adequate aldehyde to lead to the corresponding *gem*-diamine intermediate, which was then oxidised using dichloro-dicyano-1,4-benzoquinone (DDQ). Finally, pyrido-imidazodiazepinones **4** (Scheme 3) and **14** (Table 1) were isolated in low yields (5–18%), after purification by chromatography on alumina gel or by preparative chromatography. Indeed, the presence of the alkyne group led to several unidentified by products, which complicated the purification step.

**Figure:**
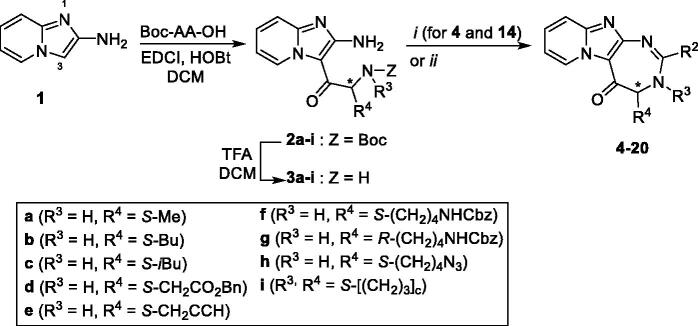
**Scheme 1**. Synthesis of pyrido-imidazodiazepinones **4**–**20**. Conditions and reagents: Boc-AA-OH: Boc-Ala-OH, Boc-Nle-OH, Boc-Leu-OH, Boc-Asp(Bn)-OH, Boc-Pra-OH, Boc-Lys(Cbz)-OH, *R*-Boc-Lys(Cbz)-OH, Boc-azidonorleucine **29** or Boc-Pro-OH; *i*: 1. NH_4_OH, H_2_O; 2. R2CHO, CHCl_3_, 60 °C; 3. DDQ, rt; *ii*: R_2_CHO, K_2_CO_3_, I_2_, THF, 65 °C. For compounds **4**–**20**, R^2^, R^3^ and R^4^ substituents are referenced in Table 1.

For other derivatives, diamines **3a-i**, as trifluoroacetate salts, were directly reacted successively with a set of aldehydes with potassium carbonate and iodine at 70 °C overnight. Initially, *tert*-butanol was used as the solvent. However, we found that tetrahydrofurane (THF) gave the same results and as it is easier to remove, this solvent was preferred for the cyclisation step. Pyrido-imidazodiazepinones **5**–**13** and **15**–**20** were finally isolated in 8–98% yield, after purification by chromatography on alumina gel. The introduction of a chiral center alpha to the carbonyl group might lead to partial racemization. However, using chiral HPLC analysis, we previously demonstrated that no epimerization occured during the synthesis of diazepine derivatives^13^.

For several compounds, the necessary aldehydes used in the cyclisation step were synthesised (Scheme 2). For compounds **6** and **10**, where R^2^ is a 4-benzophenyl or a 6-quinolinyl substituent respectively, the corresponding aldehydes **24** and **26** were prepared, using a 3-step procedure, starting from their respective carboxylic acids. Briefly, 4-benzoyl-benzoic acid or 6-quinolinecarboxylic acid were esterified using ethanol in acidic medium. The ethyl esters **23** or **25** were then reduced into their corresponding alcohols, using lithium aluminium hydride. These latter were then oxidised using manganese (II) oxide to generate 4-benzoyl-benzaldehyde **25** and quinoline-6-carbaldehyde **26**. For compound **4′** bearing an alkyne group, the TMS protected 4-formylphenylacetylene **27** was prepared from 4-bromobenzaldehyde, using a Sonogahira reaction, according to the procedure described by Cummings et al. (Scheme 3)^24^. Boc protecting-3-indolecarboxaldehyde **28** was prepared from 3-indolecarboxaldehyde and di-*tert*-butyl dicarbonate, using the procedure described by Pelcman et al. (Scheme 3)^25^. After cyclisation and oxidation, compounds **4′** and **9′** were then unprotected, using respectively a solution of tetrabutylammonium fluoride in THF or a mixture of TFA/DCM, according to Scheme 3.

**Figure:**
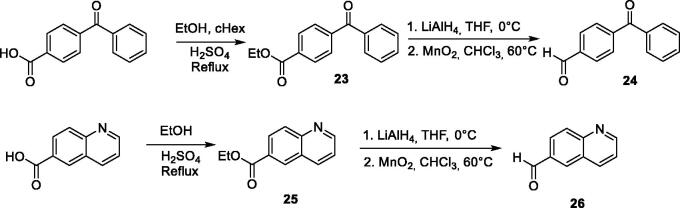
**Scheme 2**. Synthesis of aldehydes **24** and **26.**

**Figure:**
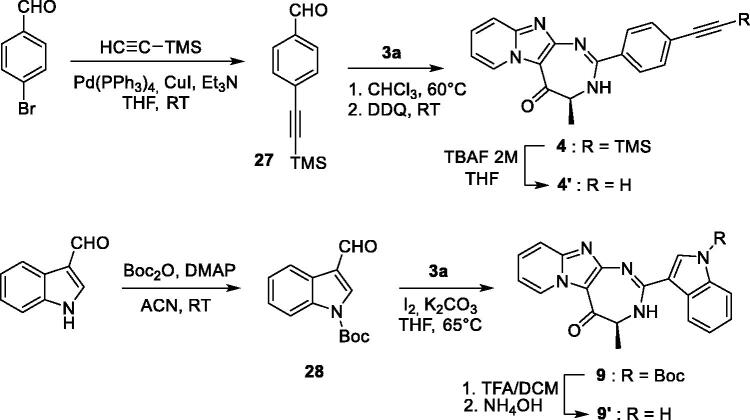
**Scheme 3**. Synthesis of compounds **4’** and **9’**.

**Scheme 4.**
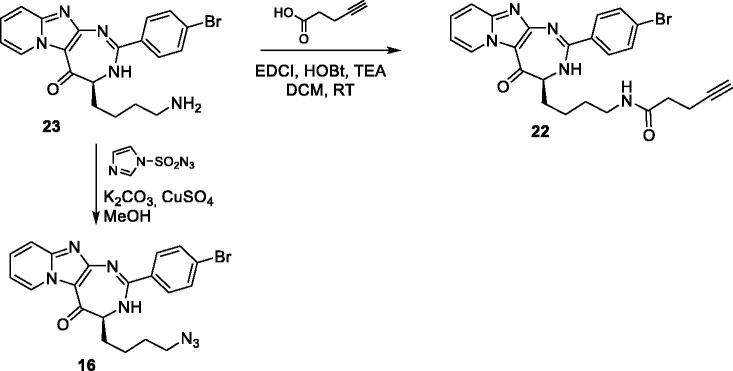
Synthesis of compounds **16** and **22**.

For 4-azidobutyl derivatives **16**–**18**, introduction of the azido group was studied using two strategies. Initially, the azido group was introduced in the final step of the sequence by diazo transfer on the free amino derivative **23**, using imidazo-1-sulfonyl azide in presence of potassium carbonate and copper sulfate (Scheme 4). The imidazolyl reactant was preferred to the classical sodium azide, because this reagent is more stable and easier to handle^26^. The diazo transfer reaction proceeded smoothly and led to several by-products, which complicated the purification step. Compounds **16**–**18** were finally isolated in 10% yields from compound **1** (5-step procedure). In a second approach, Boc-6-azido-Nle-OH **29** was prepared from Boc-Lys-OH, according to the procedure described by Shoonen et al.^15^ Acylation of **1** with compound **29** led to compound **3 h** in 71% yield. The Boc group was then deprotected, using a mixture of trifluoroacetic acid/dichloromethane (50/50 v/v) and the resulting diamine was cyclized with 4-bromobenzaldehyde to offer compound **16** in 30% yield. Compared with the initial 5-step sequence, the 4-step procedure led to a better global yield from compound **1** (21% *vs* 10%) and compounds were easier to purify. Finally, alkyne derivative **22** was prepared in 94% yields by acylation of the amine **23** with pentynoic acid (Scheme 4).

### Structure-activity relationships

The *in vitro* cytotoxic activity of diazepine derivatives was measured towards A375 and MDA-MB-435 melanoma cell lines, using neutral red uptake viability assay. The half maximal inhibitory concentrations (IC_50_) values are reported in Table 1.

We started our investigation by studying the position 2 of the diazepine ring. From our previous studies, we observed that 4-bromophenyl, 4-iodophenyl, 2,4-dichlorophenyl and 1- or 2-naphthyl substituents were the most preferred groups in this position^11^, which could suggest that such substituents occupy a large hydrophobic pocket in the target. To further study this position, we investigated the substitution of this position by several other hydrophobic groups.

Firstly, an alkyne, an azido group or a phenylketone were introduced in *para* position of the phenyl substituent, because we previously demonstrated that the *para* position was the most favourable position for the phenyl substitution^11^. The results are reported in Table 1 (entries 2–4). The activities were comparable for the two tested melanoma cell lines, even if the MDA-MB-435 cell line was more sensitive to compounds **4′** and **5**, as also observed with JMV5038 (Table 1, entry 1). Whereas the substitution of the bromine atom of JMV5038 by an azido group (compound **5**) or, in a lesser extent, by an alkyne group (compound **4′**) maintained the cytotoxic activity in the two melanoma cell lines tested, the benzophenone derivative **6** was totally inactive, probably due to the steric hindrance brought by the supplementary phenyl ring. Indeed, the molecular volume of compound **6**, predicted using the molsoft cheminformatic tool, is about 20% greater than that of JMV5038 (397 *vs* 317 Å^3^, Table 2), whereas for compounds **4′** and **5**, the molecular volume increase is only 2% and 4% respectively (324 and 330 Å^3^), suggesting that the molecular volume of the substituent occupying the position 2 of the diazepine ring is a crucial parameter for the biological activity.

**Table 2.** Some calculated physicochemical parameters of compounds modified on position 2.

Entry	Compound	*c*LogP^a^	TPSA (Å^2^)^a^	Molecular volume (Å^3^)^a^
1	**JMV5038**	2.83	43.63	316.84
2	**1-naphthyl derivative^b^**	3.31	43.36	344.46
3	**2-naphthyl derivative^b^**	3.43	43.36	344.32
4	**4′**	2.53	43.63	330.95
5	**5**	2.74	80.94	324.19
6	**6**	3.24	57.10	397.26
7	**7**	2.47	53.19	329.84
8	**8**	2.93	44.38	339.6
9	**9′**	2.20	53.50	333.93
10	**10**	2.43	52.39	339.34
11	**11**	2.53	51.96	339.34
12	**12**	1.84	64.04	370.59

^a^Calculated using the molsoft chemoinformatic tool; ^b^Naphthyl derivatives were described in ref. ^11^.

Bold values correspond to compounds number.

Introduction of [6 + 6] or [6 + 5]-heterocycles was then investigated at position 2. Surprisingly, whereas the MDA-MB-435 cell line was more sensitive to JMV5038 and to compounds **4′** and **5** than the A375 cell line (see entries 1–3, Table 1), these compounds were around 3 times less active on MDA-MB-435 than on A375 cells (see for example, compounds **7**, **10** or **11**, entries 5, 8 and 9, Table 1). This result could suggest that the molecular mechanism involved in the cytotoxic activity of compounds **7**, **10** and **11** is not totally similar to the one involved in the activity of JMV5038. For [6 + 6] derivatives, the coumarinyl group totally abolished the activity (compound **12**), whereas the 2- or 6-quinolinyl groups were tolerated, even if the biological activities were weaker than those obtained with their previously described naphthyl analogues (IC_50_ = 35.49 and 30.71 µM for compounds **10** and **11** respectively, compared to IC_50_ of 1.4 µM for 1- or 2-naphthyl derivatives on MDA-MB-435 cell line^11^). Indeed, compound **12** possesses a large molecular volume (370.59 Å^3^), which could be detrimental for the activity, as observed with compound **6**. Moreover, in comparison with their naphthyl analogues, compounds **10**, **11** and **12** possess a greater polar surface (Topological Polar Surface Area, TPSA) and a weaker *c*LogP (Table 2). Whereas an increase of the polar surface was well tolerated in the case of the azido compound **5** (TPSA increase of 185% for compound **5** compared to JMV5038) suggesting that this parameter is not crucial for the activity of these series of compounds, the lipophilicity seems to be more important. Indeed, the *c*LogP of compounds **10** to **12** (2.43, 2.53 and 1.84 respectively) were weaker or slightly weaker than those calculated for JMV5038 and its 1- or 2-naphthyl analogues (2.83 and 3.31/3.43, Table 2). Concerning the [6 + 5] derivatives, best activities on A375 cell line were obtained with the benzothiophene derivative **8** (IC_50_ = 7.97 µM), followed by the benzofuran derivative **7** (IC_50_ = 8.47 µM). Indolyl derivative **9′** was totally inactive on the two melanoma cell lines. Among the three [6 + 5] analogues tested, which are known to be bioisosters of the naphthyl group^27^, indole derivative **9′** is slightly more hydrophilic (*c*LogP = 2.20) than the benzofuran and the benzothiophene derivatives **7** and **8** (*c*LogP = 2.47 and 2.93 respectively, Table 2), confirming that the lipophilicity is a crucial parameter for the choice of substituents to introduce in position 2.

To further evaluate the influence of some physicochemical parameters on the cytotoxic activity of compounds modified at position 2 (i.e. compounds **4′−12**), their IC_50_ values were graphed as a function of three commonly used molecular descriptors (Figure 2), namely *c*LogP, molecular volume and TPSA. Whereas no correlation were observed between TPSA and IC_50_ (correlation coefficient of 0.22 and 0.13 for A375 and MDA-MB-435 cell lines respectively, *p* values > 0.1, data not shown), a statistically significant correlation was obtained when the IC_50_ values were compared to the molecular volume of compounds **4′−12** and JMV5038, with a Pearson correlation coefficient of 0.75 (*p* values = 0.01) for A375 cell line and 0.65 (*p* values = 0.04) for MDA-MB-435 cell line. Thus, to obtain some activity on the two melanoma cell lines, the molecular volume of the diazepinone derivative need to be less than 350 Å^3^ (except for compound **9′**, see below) and with a cut off of around 330 Å^3^ to obtain an IC_50_ less than 10 µM (Figure 2(A,B)). Thus, a large substituent like a benzophenone (compound **6**) or a coumarinyl group (compound **12**) led probably to a steric clash with the target and to the loss of the activity. Then, the influence of the *c*LogP was investigated. After removing compounds **6** and **12** for a too high molecular volume, a statistically significant correlation was obtained when the IC_50_ were compared to the *c*LogP, with a correlation coefficient of − 0.76 (p values = 0.03) for A375 and − 0.81 (*p* values = 0.01) for MDA-MB-435 (Figure 2(C,D)). Thus, a *c*LogP greater than 2.4 was required to obtain a cytotoxic activity (IC_50_ ≤ 10 µM) within these series of compounds. This result could explain that the indole derivative **9′**, even if it possesses a molecular volume less than 350 Å^3^, presents a cLogP slightly too low to be active (namely 2.20, Table 2).

**Figure 3.**
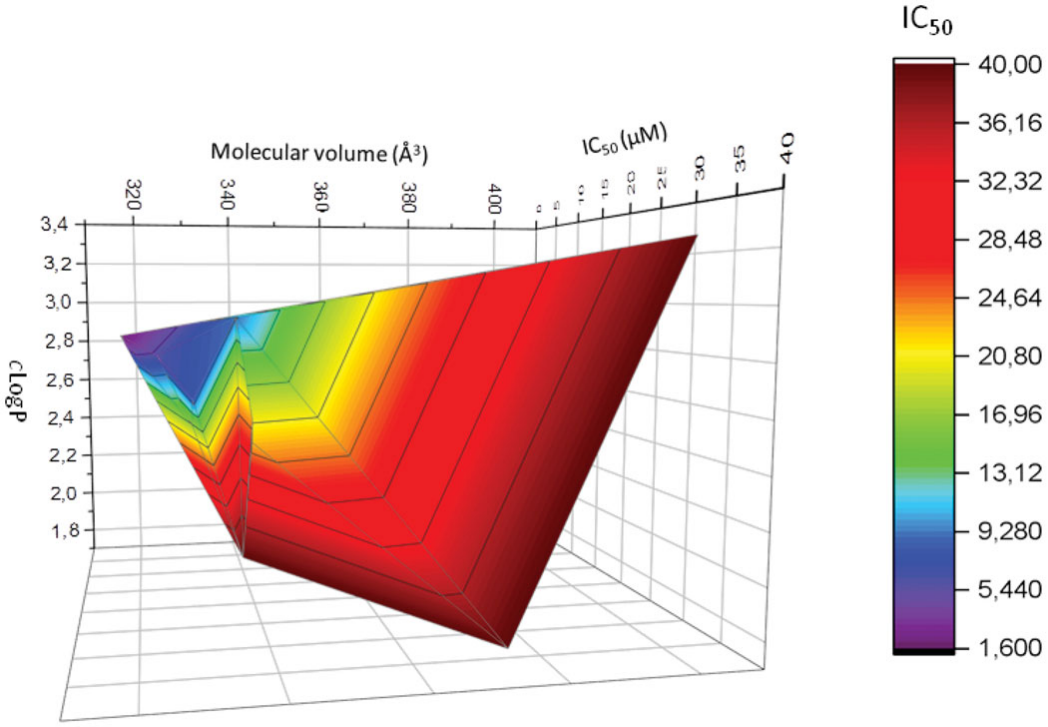
Correlation 3 D-plot between calculated molecular volume, cLogP and cytotoxic activity of compounds modified in position *R*^2^ (mean of IC_50_ determined on MDA-MB-435 and A375 melanoma cell lines).

Finally, a 3D diagram was built to visualise the influence of these two physicochemical parameters on the cytotoxic activity of compounds modified at position 2 (mean of the IC_50_ obtained on the two melanoma cell lines). Figure 3 shows clearly that a *c*LogP between ∼2.5 and 2.8, associated with a molecular volume between 310 and 350 Å^3^ are necessary to obtained active compounds in these series of compounds.

To resume the RSA study of the position 2, compound **5** showed the best activity of all derivatives modified in this position, with cytotoxic activities against the two tested melanoma cell lines closed to those obtained with JMV5038. Azido group is known to exhibit some properties in common with halogens and in particular this group resembles a bromo substituent in terms of approximate size, polarity and electronic properties^28^, which could explain that compound **5** presented a similar profile of activity to JMV5038.

Then, we turned our attention to the SAR study of the position 4 of the diazepine ring, keeping constant the 4-bromophenyl group at R^2^ (i.e. compounds **13**–**17** and **19**–**22**). We previously demonstrated that position 4 could tolerate short linear alkyl chains (methyl, ethyl or hydroxymethyl), but also bulky groups like tert-butyl, but not too close to the diazepine ring^11^. To further study this position, several other chains were introduced into position 4. n-Butyl and n-azidobutyl chains are tolerated (compounds **13** and **16** respectively), with a good activity obtained for compound **16**, close to the activity obtained with JMV5038 (mean of IC_50_ values in MDA-MB-435 cells are 4.12 µM for compound **16** compared to 1.30 µM for JMV5038, entries 1 and 14, Table 1). Surprisingly, we observed that the stereochemistry is not so crucial for the activity when an *n*-azidobutyl chain was introduced at position 4 of the diazepine ring. Whereas a *R*-methyl group at position 4 led to a totally inactive compound^10^, a *R*-azidobutyl chain led only to a slight decrease of the activity on A375 cell line, and to a more significant decrease for the MDA-MB-435 cell line (see entries 14 and 15, Table 1). This result could suggest that the *n*-azidobutyl derivatives were not positioned exactly in the same way as the methyl analogues in the target or that the mechanisms of action involved in the cytotoxicity are not totally similar. However, as also observed with the 4-methyl derivatives, the presence of a bromophenyl substituent at *R*^2^ is still necessary, because compound **18** is totally inactive (entry 16, Table 1). Introduction of a ramified isobutyl chain (compound **15**) led also to a loss of the activity (entry 13, Table 1). This result is not surprising, because we already reported that an isopropyl chain in this position is unfavourable for the activity^11^, suggesting that substituents borne by position 4 of the diazepine ring occupied a relatively narrow pocket in the target. In the same way, a less flexible chain bearing an alkyne group (compound **14**) was not tolerated, as well as a long chain (compound **22**) (Table 1, entries 12 and 20 respectively). Surprisingly, some activity was recovered with the bulky benzyl ester derivative (compound **19**, entry 17, Table 1), whereas compounds with chains bearing a phenyl ring (namely a benzyl or a -(CH_2_)_4_NHCbz substituent) were previously determined to be inactive^10,11^. This result could suggest that compound **19** could be a pro-drug. Indeed, compound **19** was partially converted into its corresponding acid **21**, with a hydrolysis rate of 65.5%, determined by HPLC-MS, after 72 h in the presence of melanoma cells and culture medium. The acid derivative **21** was prepared and tested, but this compound revealed to be inactive (Table 1, entry 19), which seems to invalidate the pro-drug hypothesis. At this time we could not exclude that the main problem with compound **21** is not its activity towards the target, but its increase of polarity (*c*LogP of 2.1 for compound **21**, compared to 3.97 for the ester **19**); this parameter revealing to be important for the biological activity. However, contrary to what was observed with modulations of the position 2 of the diazepine ring, no significant correlation could be highlighted between the activity of the 4-modified compounds and their *c*LogP or their molecular volume (data not shown).

Finally, the introduction of a fused tetrahydropyrrolo cycle between positions 3 and 4 (compound **20**) led to the loss of the activity. This result confirms that the position 3 of the diazepine ring needs to be unsubstituted, as already observed with the N3-methyl derivative of JMV5038^11^.

Then, all active compounds on the two melanoma cell lines (IC_50_ < 40 µM) were assessed on the NIH-3T3 mouse fibroblast cell line, in order to check the toxicity of our compounds on non-cancerous cells. Globally, the diazepine compounds were not toxic on this cell line (IC_50_ > 40 µM), except for compounds **16** and **17** bearing an n-azidobutyl chain, where a not negligible toxicity was noted (IC_50_ around 10 µM, Table 1, entries 14 and 15). As a possible modification of the mechanism of action was hypothesised for these two derivatives (see above), this phenomenon could probably be also correlated with the cytotoxicity observed on the NIH-3T3 cell line.

### *In vitro* antiproliferative activities on the NCI-60 cancer cell line panel

From the SAR study of the position 2 of the diazepine ring conducted on A375 and MDA-MB-435 cell lines, it appeared that the sensitivity of the two melanoma cell lines tested towards several compounds was quite different.

**Figure 4.**
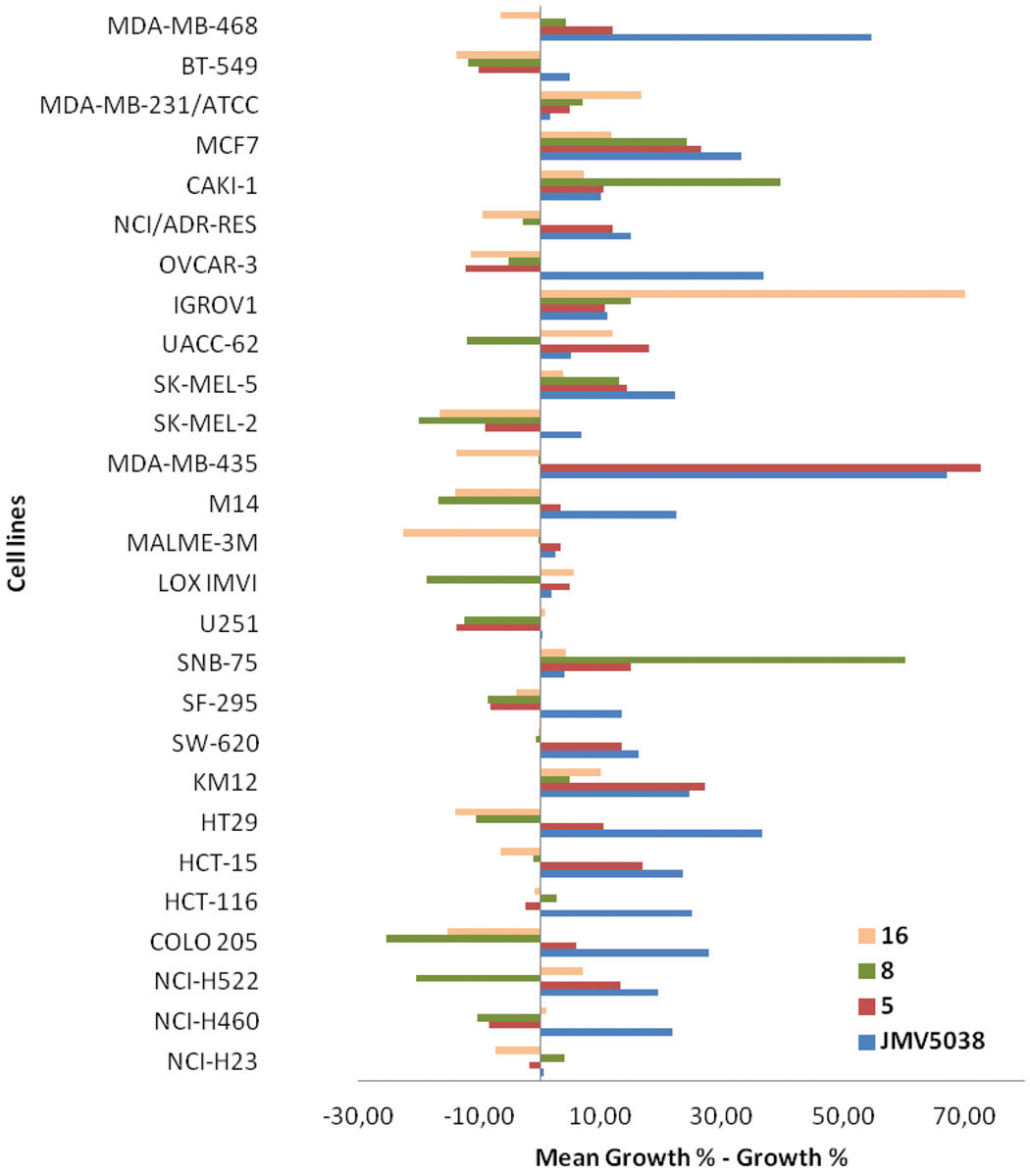
Evaluation of *in vitro* anti-cancer activity of JMV5038, compounds **5, 8** and **16** on the NCI-60 cell line panel, after 48 h of exposure at a single dose of 10 µM of each tested compound. Presented results are cell growth level determined for the 4 compounds on the 27 most JMV5038 sensitive cell lines (i.e. mean growth % – growth % > 0). For all data (60-cell lines), see Table S1, SI. The difference between the growth level of a specific cell line and the mean growth is represented by a bar. Cell lines that were more sensitive are displayed as bars that project to the right of the mean. Cell lines that were less sensitive are displayed with bars projected to the left.

Whereas a substituted phenyl group in position 2 of the diazepine (compounds **4′** and **5**) led to a similar response than JMV5038 (i.e. more active on MDA-MB-435 than on A375 cell lines, Table 1, entries 2–3), introduction of [6 + 6] or [6 + 5]-heterocycles in this position led to compounds more active on A375 than on MDA-MB-435 cell lines (compounds **7**–**8**, **10**–**11**, Table 1, entries 5–6 and 8–9). Even if the mechanism of action and the target of JMV5038 are not elucidated at this moment, the specificity of the activity profiles on different cell lines could suggest that these new derivatives did not share exactly the same mechanism involved in the cytotoxic activity. To better compare the activity profile of these two series of compounds, compounds **5** and **8** were selected and then evaluated towards a panel of sixty cancer cell lines corresponding to nine different cancer types, i.e. leukaemia, melanoma, as well as lung, colon, central nervous system, ovarian, renal, prostate and breast cancers (NCI evaluation). Indeed, it is now well established that the growth inhibitory patterns of anticancer drugs against the NCI-60 cell line panel correlated well with their modes of action^29^. For example, compounds that bind to tubulin have similar growth inhibition patterns regardless of which site on tubulin they bind^30^. Compounds **5** and **8** were chosen because they are the most active compounds in the two series of 2-modified derivatives (Table 1, entries 3 and 6). Compounds were tested using the sulforhodamine B (SRB) assay that provides a direct cell density index. The amount of dye incorporated by the cells increases with the increase of the total protein biomass. Cells were incubated for 48 h with compounds at 10 µM concentration, then cell growth was evaluated spectrophotometrically and presented as the percent of untreated control cells. Values of the most sensitive cell lines were compared to those obtained with JMV5038 (Figure 4, for all data see SI).

As suggested by our results obtained on A375 and MDA-MB-435 cell lines, the activity profile on the NCI-60 cell line panel revealed a great difference of sensitivity between the two tested compounds. Compound **5** presented a profile similar to that obtained with JMV5038 (statistically significant correlation, *r* = 0.72, *p* values < 0.01), with the best activity obtained against MDA-MB-435 melanoma cell line (growth inhibition percentage of 87%), as already observed with JMV5038 and several other analogues^11^. On the other hand, the activity profile obtained with compound **8** was very different from that of JMV5038 and no correlation was obtained (*r* = 0.07, *p* values = 0.63), confirming that the mechanism of action involved in the cytotoxic activity is probably not totally comparable to that of JMV5038.

Concerning the compounds modified in position 4 (compounds **13**–**22**), the response towards the two tested melanoma cell lines appeared also different (Table 1, entries 11–20). Compounds **13**, **16**, **17** and **19** were more active on A375 cell line than on MDA-MB-435 (inverse of the JMV5038 profile). Moreover, compounds **16** and **17** led also to some toxicity on NIH-3T3 cell line, indicating a probably modified mechanism of action, compared to JMV5038. This result was confirmed by analysing the activity profile of compound **16** on the NCI-60 cancer cell line panel (Figure 4), where no correlation was obtained with JMV5038 (*r* = 0.06, *p* values = 0.66).

### DNA cell cycle analysis

**Figure 5.**
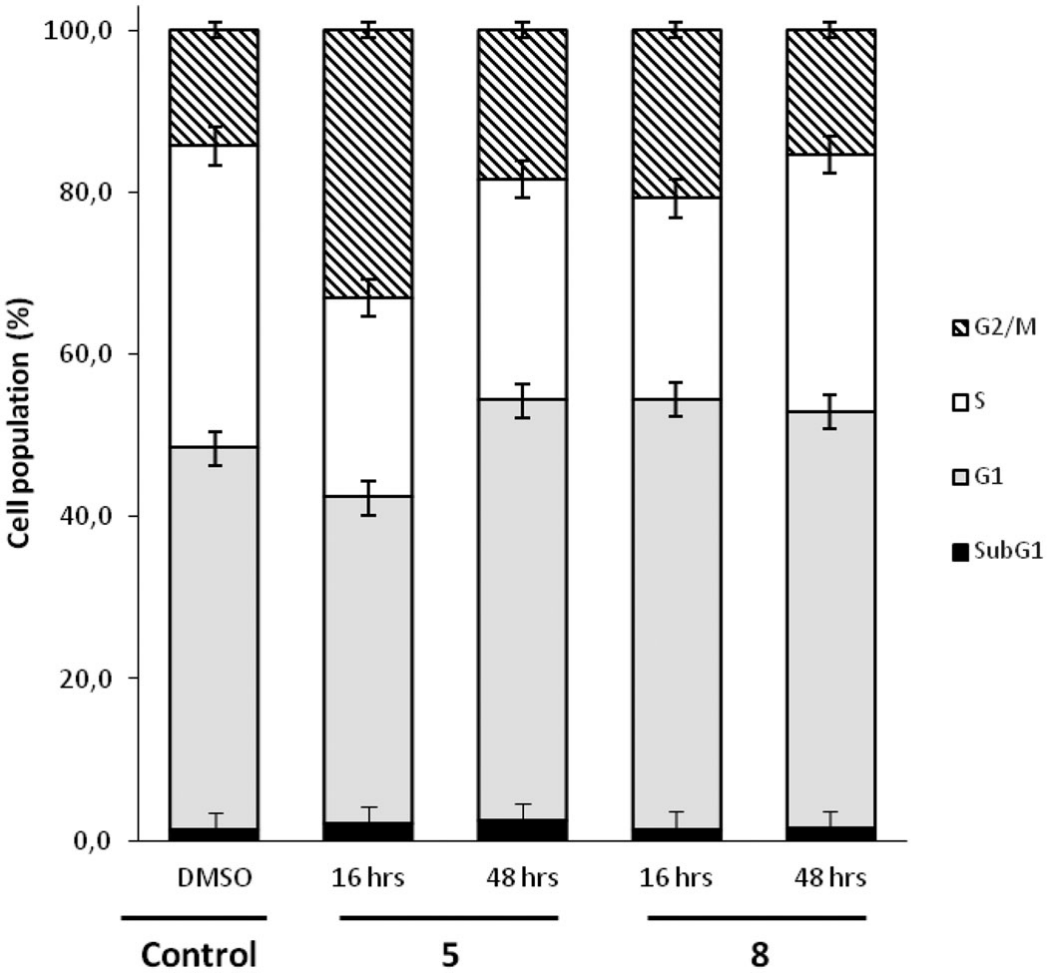
Flow cytometric analyses showing the cell cycle distribution of MDA-MB-435 cells treated with DMSO or 5 µM concentration of compound **5** or **8** for 16 h or 48 h. The panels represent distribution of cells (%) in Sub G1-, G1-, S-, G2/M- phase of the cell cycle.

Compounds **5** and **8** were then selected for further characterisation, because these two compounds showed different activity profiles (similar to that of JMV5038 for compound **5** and different for compound **8**). Thus, compound **5** could probably shared a common mechanism of action with JMV5038, contrary to compound **8**. DNA cell cycle analysis was performed using MDA-MB-435 cells treated with either compound **5** or compound **8**, and compared to that obtained with cells treated with vehicle (DMSO) as a negative control^10^. Cell cycle pattern changes of MDA-MB-435 cells were investigated using fluorescence-activated cell sorting (FACS) (Figure 5 and Figure S1, SI). In this experiment MDA-MB-435 cells were treated with or without 5 µM concentration of compound **5** or **8** for 16 h and 48 h.

After 16 h, compound **5** increased the proportion of cells in G2/M phase (33.1% compared to 14.3% in control). At 48 h, this increase was still present, but less marked (18.4% *vs* 14.3%). A slightly increase of apoptosis in subG1 phase was also observed (2.4% compared to 1.2% in control). These induced changes in the cell cycle were quite comparable with those already observed in cells treated with JMV5038^10^. For compound **8**, a slightly increase of the proportion of cells in G2/M phase was also observed (20.8% at 16 h and 15.4% at 48 h *vs* 14.3% in control). However, compound **8** increased the proportion of cells in phase G1 at 16 h (53.0% compared to 47.1% in control). This profile was opposite from that observed with compound **5**, which led to a decrease of cells in G1 phase at 16 h. The activity profile of compounds **5** and **8** on the NCI-60 cancer cell lines was quite different and this dissimilarity was also observable through the analysis of the cell cycle, with a slightly shifted distribution of DNA, particularly in the G1 phase.

### Morphological changes of cells

**Figure 6.**
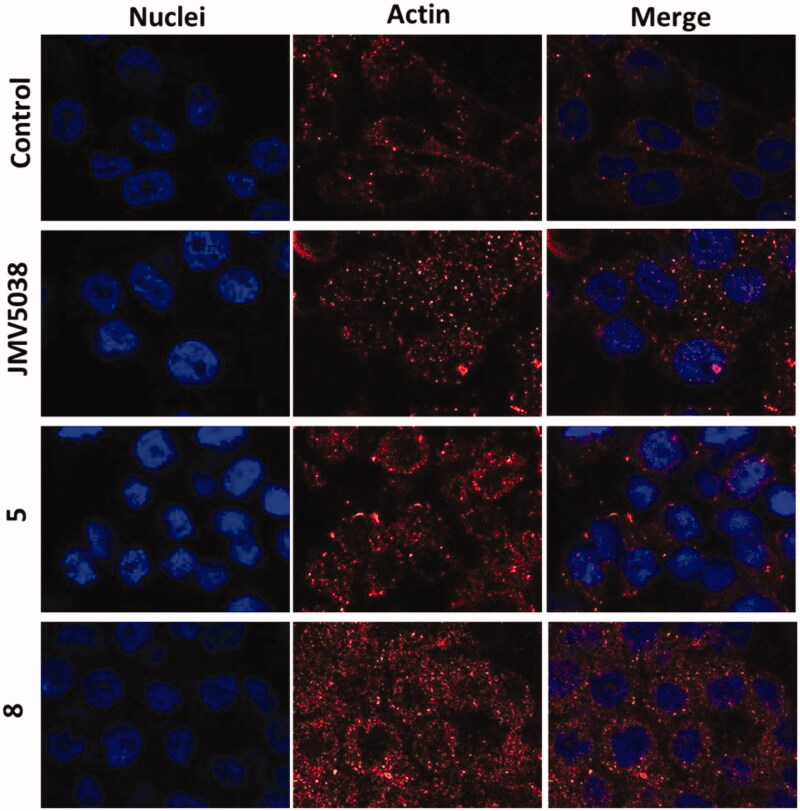
Confocal microscopy of actin localised in fixed MDA-MB-435 cells. Cells were incubated with 5 µM concentration of JMV5038, compound **5**, compound **8** or DMSO (control) for 20 h. Nuclei were stained with Hoechst 33342. AlexaFluor568 and nuclei were excited at 543 and 405 nm, and visualised at 620 (red) and 450 nm (blue) respectively.

In our previous study, we reported that JMV5038 led to changes in the cell morphology, concomitant with a disruption of the actin network^10^. Morphological changes in MDA-MB-435 cells induced by compounds **5** or **8** were investigated and compared to those induced by JMV5038. For this purpose, fixed and actin immunostained MDA-MB-435 cells were imaged by confocal microscopy (Figure 6). Control cells treated with DMSO (20 h, 5 µM concentration) were spread and the tensile forces of actin stress fibres stretched the cell-adhesive phenotype. In contrast, cells treated with compounds **5** or **8** (20 h, 5 µM concentration) showed the most recognisable actin-morphological features, comparable with those obtained with cells treated with JMV5038. Even if compounds **5** and **8** showed a different profile of activity on the two tested melanoma cell lines and on the NCI-60 cell line panel, the morphological changes induced by the three tested compounds were very similar. A diffuse distribution of non-polymerized actin, associated with a disorganisation of the actin cytoskeleton and altered malignant melanoma cell morphology were observed.

## Conclusion

Nineteen new pyrido-imidazodiazepinone derivatives were synthesised, bearing various substituents at positions 2, 3 and 4 of the diazepine ring. Their cytotoxic activities against A375 and MDA-MB-435 melanoma cell lines were evaluated, as well as their potential toxicity against NIH-3T3 fibroblast cells. Introduction of a 4-ethynyl-, a 4-azidophenyl substituent was tolerated onto the position 2 of the diazepine ring and led to compounds with a similar *in vitro* activity on melanoma cells and with no apparent toxicity on NIH-3T3 cells. On the other hand, introduction of a bicyclic and heteroaromatic group on this position led to compounds possessing a significant difference of activity towards the two tested melanoma cell lines. These results were confirmed by comparing the activity profile of compounds **5** and **8** on the NCI-60 cell line panel, indicating that the mechanisms of action of these two compounds are not exactly similar. This dissimilarity was also observed through a slightly shifted distribution of the cell cycle phases. However, both compounds led to comparable modifications on cells morphology. SAR studies revealed also that the molecular volume of the substituent introduced onto the position 2 of the diazepine ring, as well as the *c*LogP of the molecule, were two crucial parameters to maintain the activity. Thus, a molecular volume less than 350 Å3 and a *c*LogP greater than ∼2.5 were necessary to maintain the activity. These results will guide the choice of the substituents to be introduced at this position in next studies. Then, modulations of the position 4 of the diazepinone revealed that a butyl chain, an azidobutyl chain or an ester group were tolerated, even if the azido-alkyl group led to some toxicity on the NIH-3T3 cells. However, such azido compounds could be particularly interesting to introduce further modulations using click-chemistry. In particular, introduction of a biotin or a fluorescent moiety on these compounds could be helpful to determine their cellular localisation as well as their pharmacological target using a proteomic approach.

## Supplementary Material

Supplemental Material
