# Supplementary material for: Imidazopyridine-fused [1,3]diazepinones: modulations of positions 2 to 4 and their impacts on the anti-melanoma activity
Source: J Enzyme Inhib Med Chem. 2020 Apr 5;35(1):935–49. doi: 10.1080/14756366.2020.1748024 (PMC7170309; doi:10.1080/14756366.2020.1748024)
Supplement: Supplemental Material [file IENZ_A_1748024_SM0086.pdf]

Imidazopyridine-fused [1,3]-diazepinones part 3: Modulations of positions 2 and 4 and their impacts on the anti-melanoma activity.

Paul Le Baccon-Sollier,[a†] Yohan Malki,[a†] Morgane Maye,[a] Lamiaa M.A. Ali,[a,b] Laure Lichon,[a] Pierre Cuq,[a] Laure-Anaïs Vincent,[a] Nicolas Masurier[a*]

[a] Institut des Biomolécules Max Mousseron, UMR 5247, CNRS, Universités Montpellier, UFR des Sciences Pharmaceutiques et Biologiques, 15 Avenue Charles Flahault, 34093 Montpellier Cedex 5, France.
[b] Department of Biochemistry, Medical Research Institute, University of Alexandria, 21311 Alexandria, Egypt.

***Corresponding author:** Nicolas Masurier. Tel: + (33) 4 11 75 96 42, Fax: + (33) 4 11 75 96 41, E-mail: nicolas.masurier@umontpellier.fr
† These authors contributed equally to this work.

Supporting Information

Table of Contents

I. Copies of NMR spectra 2
Compound **26** ($CDCl_3$) 2
Compound **2b** ($CDCl_3$) 3
Compound **2e** ($CDCl_3$) 4
Compound **2h** ($CDCl_3$) 5
Compound **4** ($CDCl_3$) 6
Compound **4'** ($CDCl_3$) 7
Compound **5** ($CDCl_3$) 8
Compound **6** (DMSO d_6) 9
Compound **7** ($CDCl_3$) 10
Compound **8** ($CDCl_3$) 11
Compound **9** ($CDCl_3$) 12
Compound **9'** ($CDCl_3$) 13
Compound **10** ($CDCl_3$) 14
Compound **11** ($CDCl_3$) 15
Compound **12** (DMSO d_6) 16
Compound **13** ($CDCl_3$) 17
Compound **15** ($CDCl_3$) 18
Compound **16** ($CDCl_3$) 19
Compound **18** ($CDCl_3$) 20
Compound **19** ($CDCl_3$) 21
Compound **20** ($CDCl_3$) 22
Compound **21** (DMSO d_6) 23
Compound **22** ($CDCl_3$) 24

II. NCI screening data at 10 µM (SRB assay) - Table S1 25

III. DNA cell cycle analysis - Figure S1 28

I. Copies of NMR spectra

Compound **26** ($CDCl_3$)

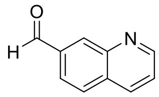

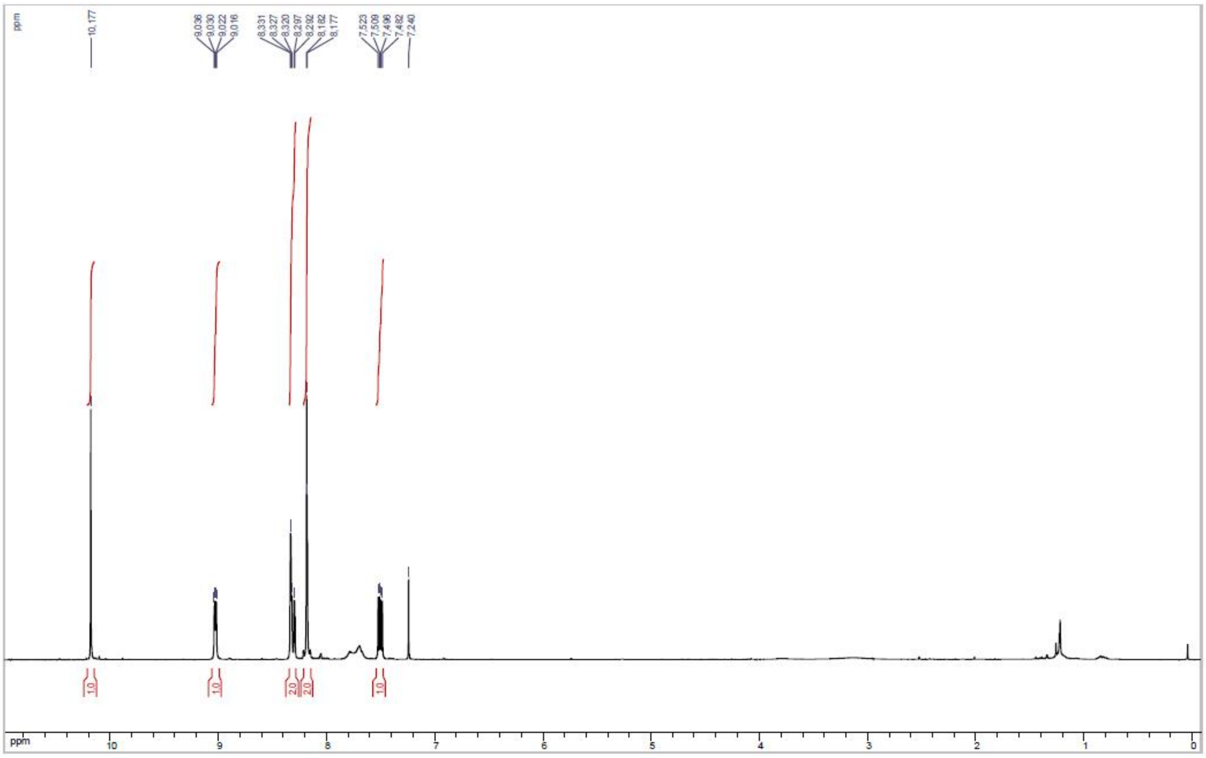

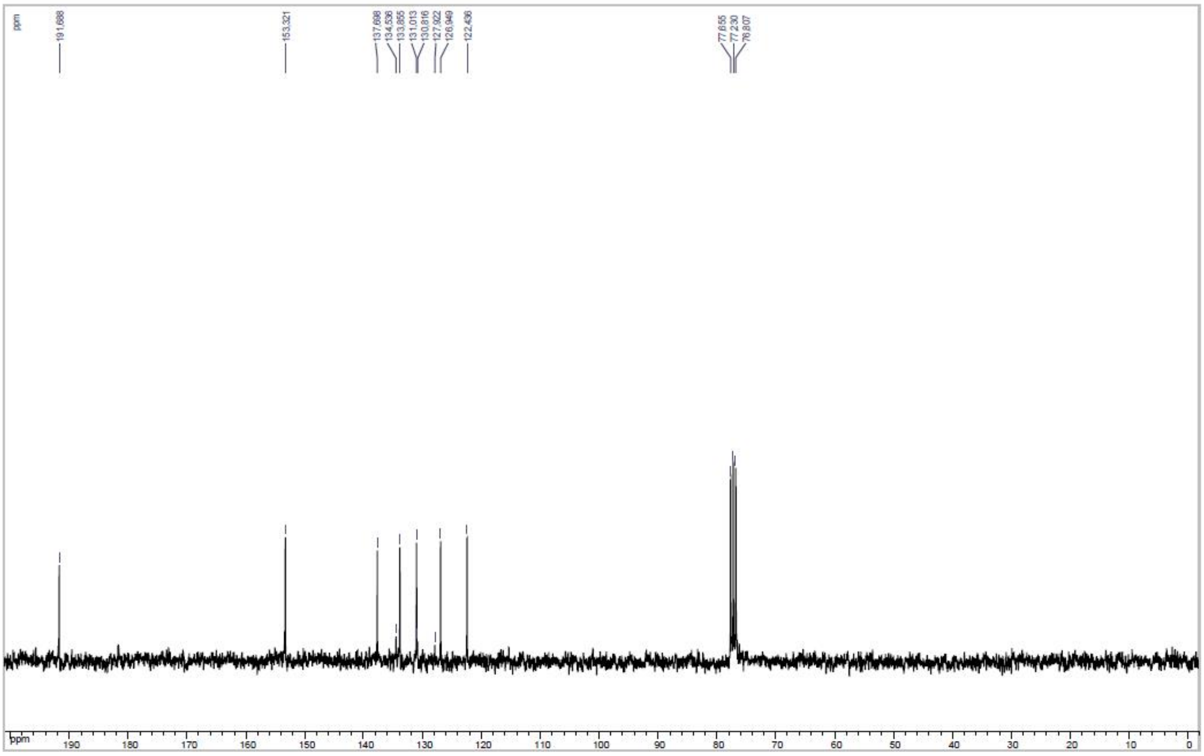

Compound **2b** ($CDCl_3$)

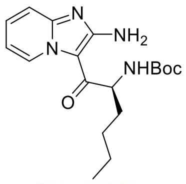

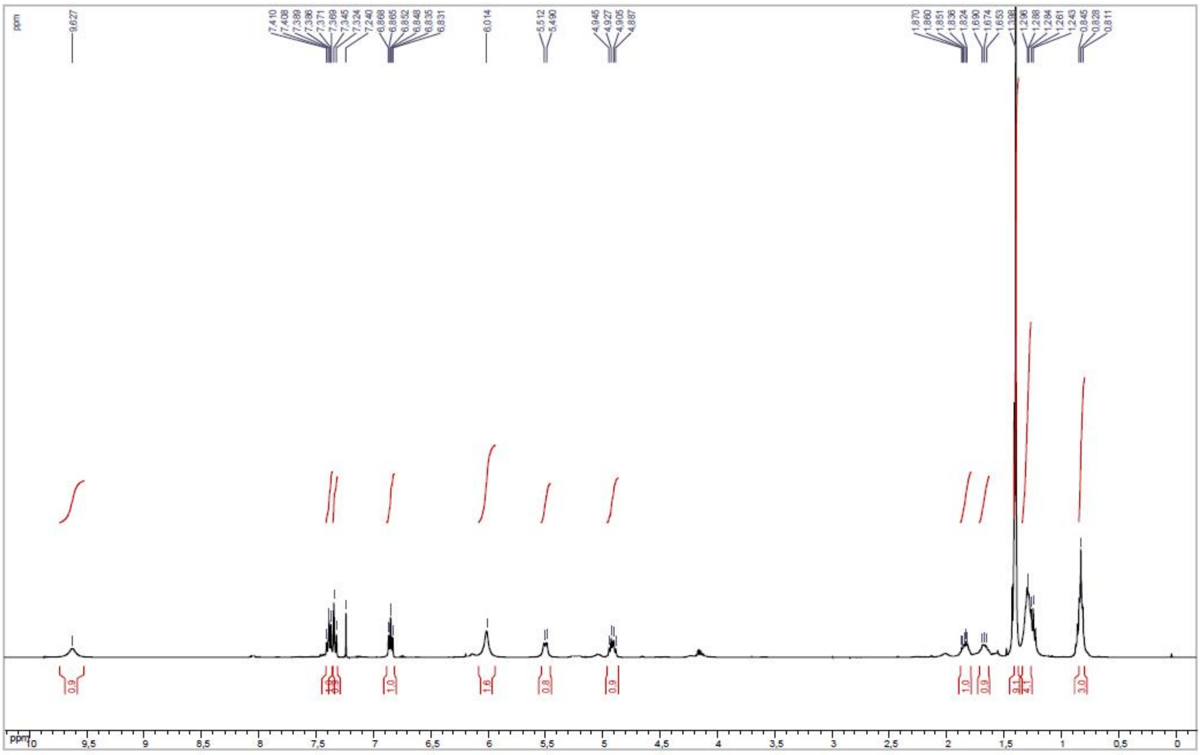

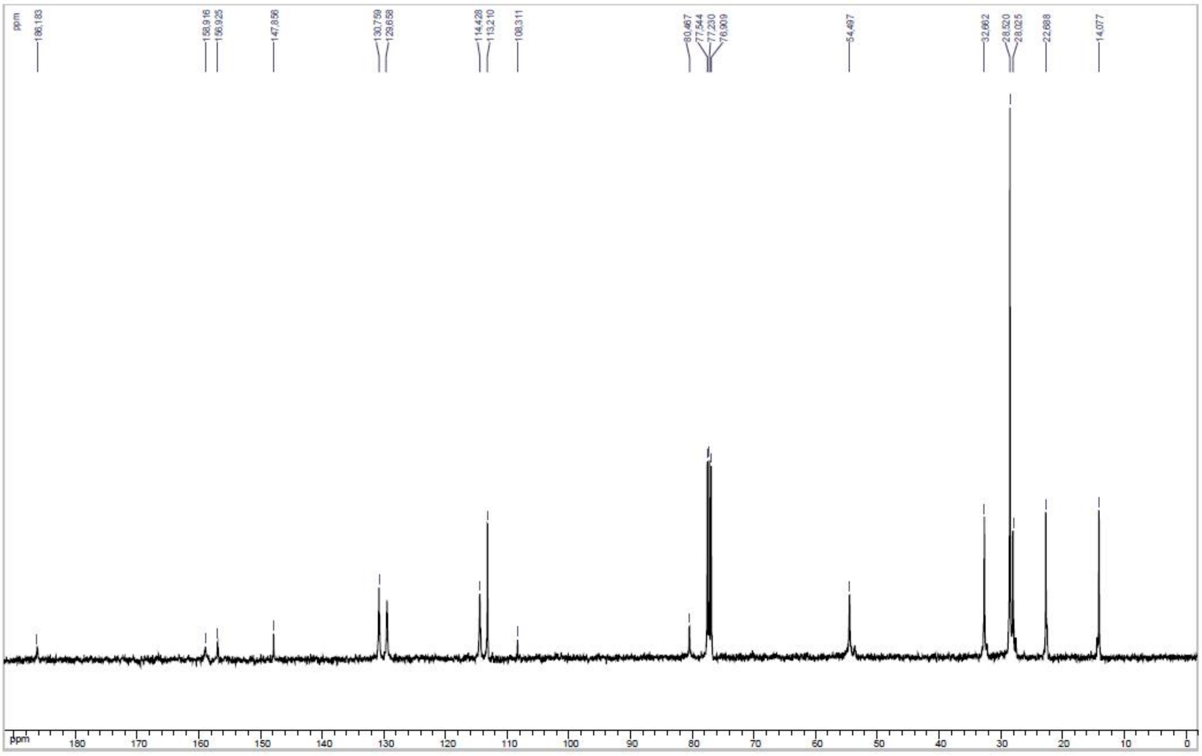

Compound **2e** ($CDCl_3$)

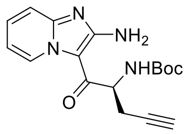

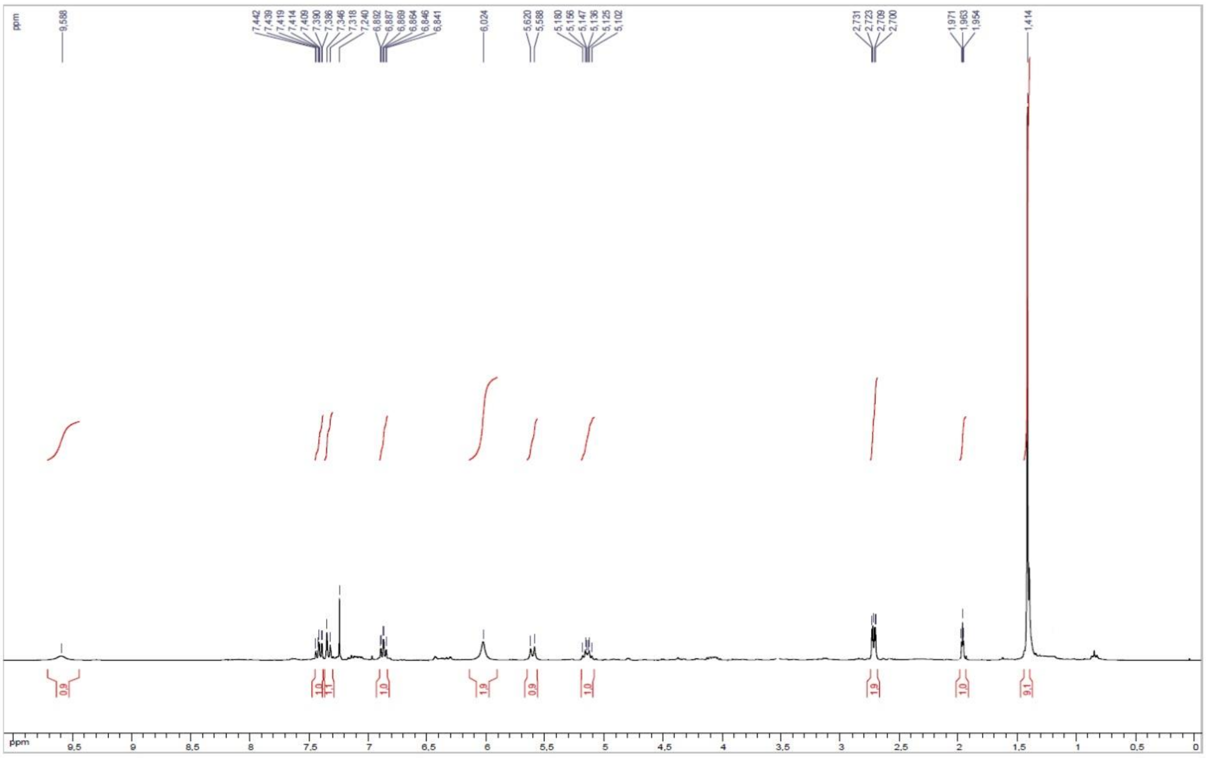

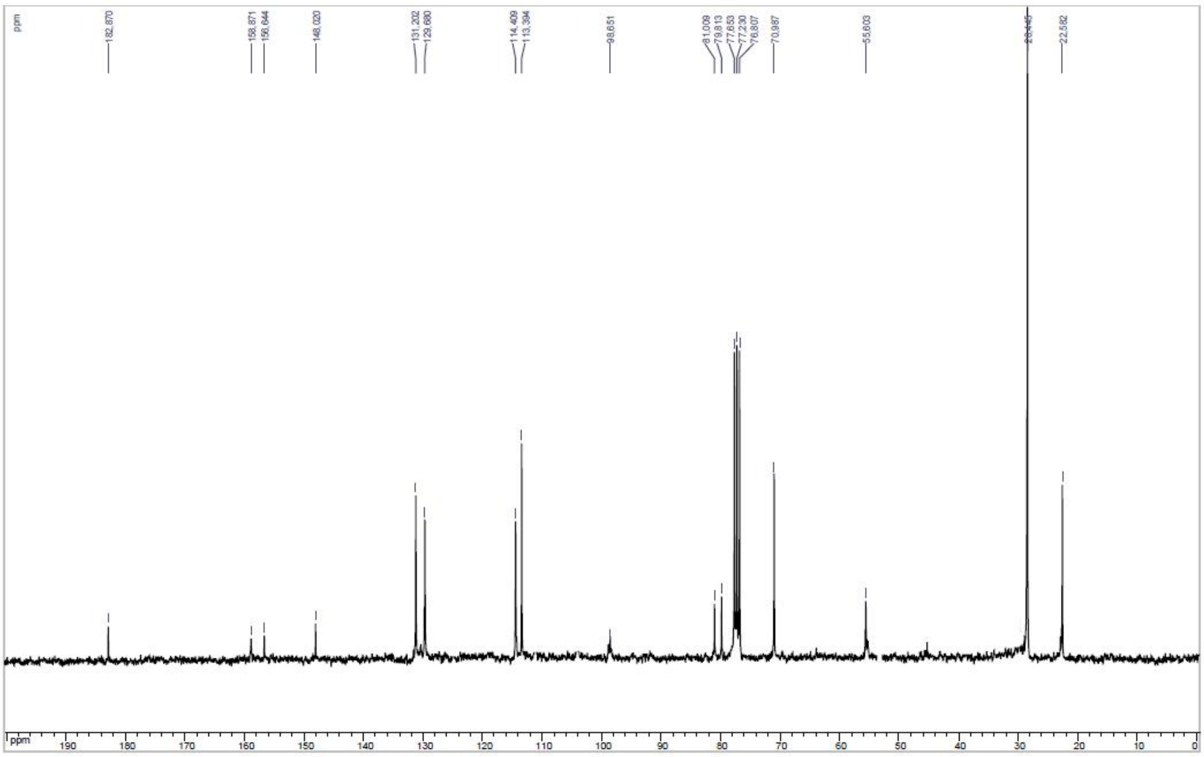

Compound **2h** ($CDCl_3$)

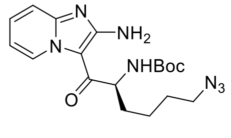

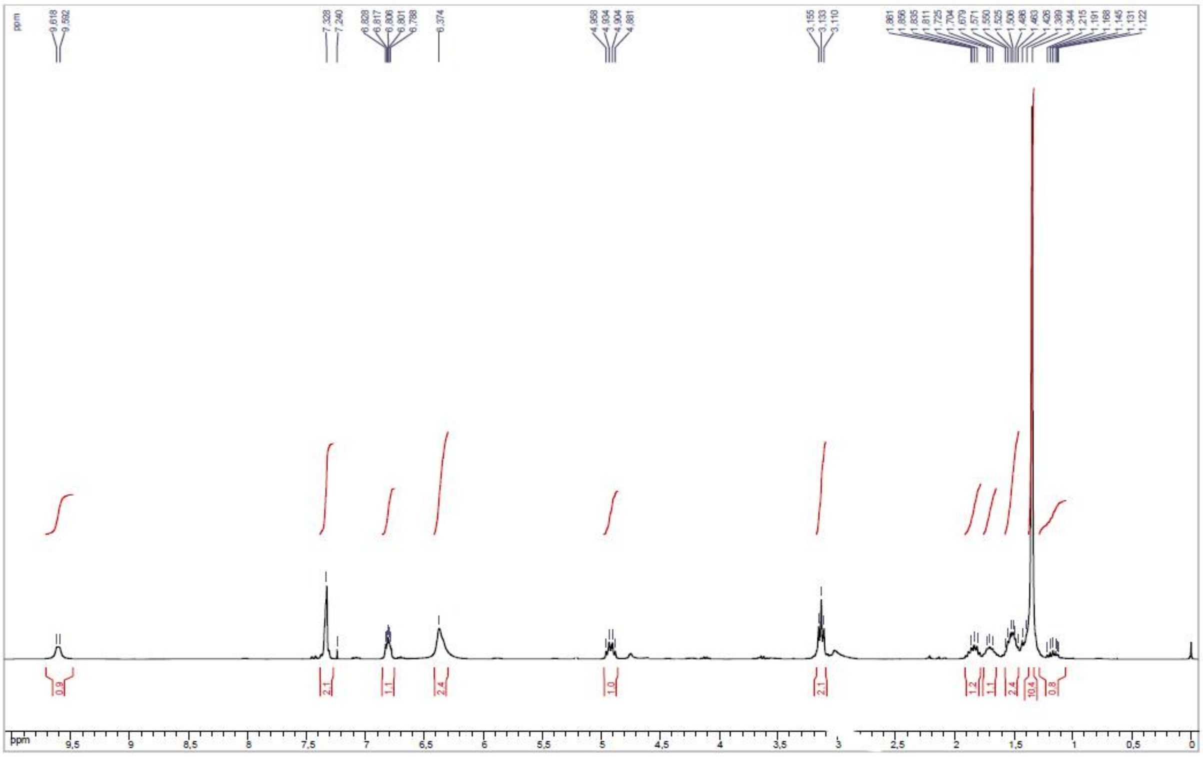

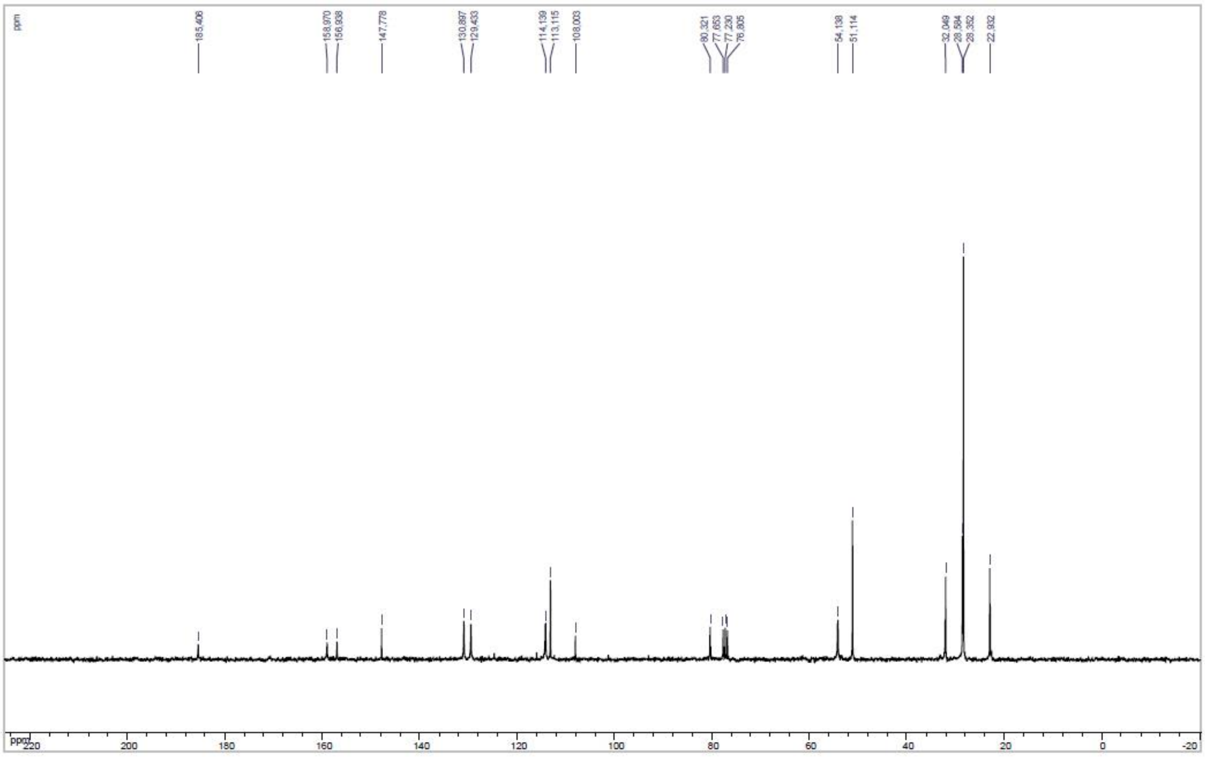

Compound **4** ($CDCl_3$)

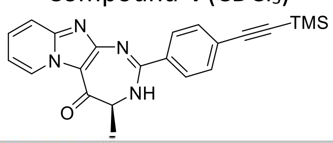

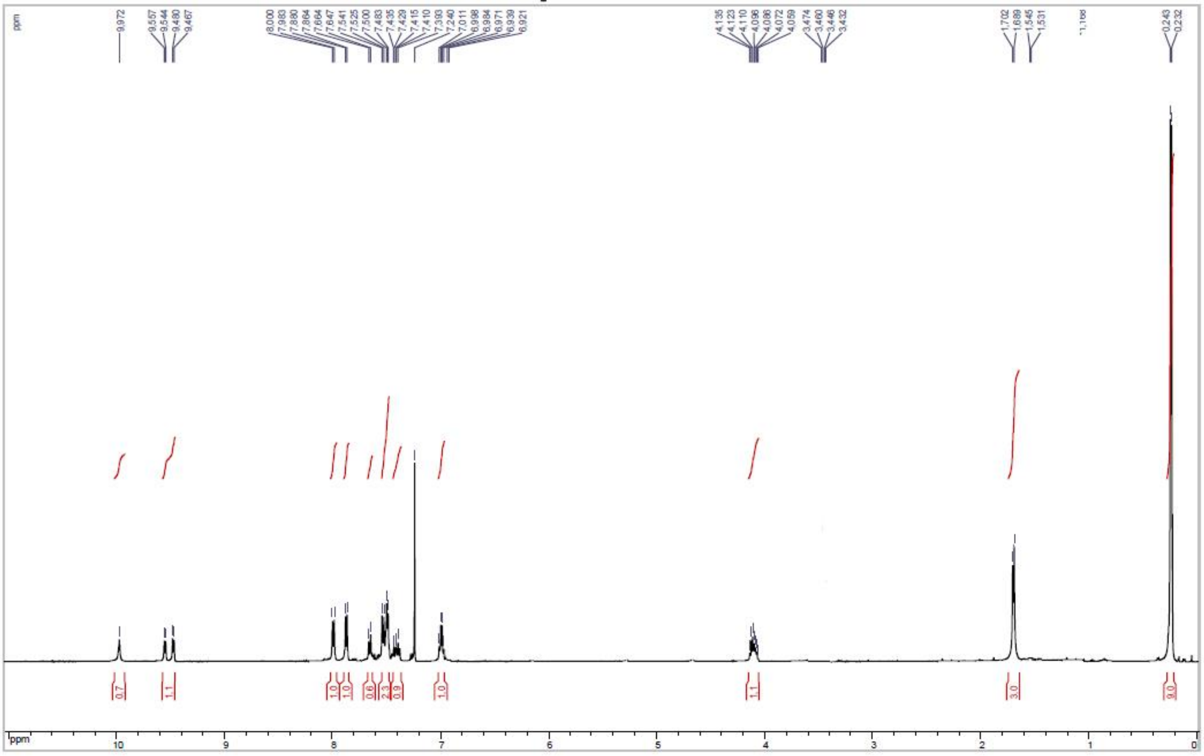

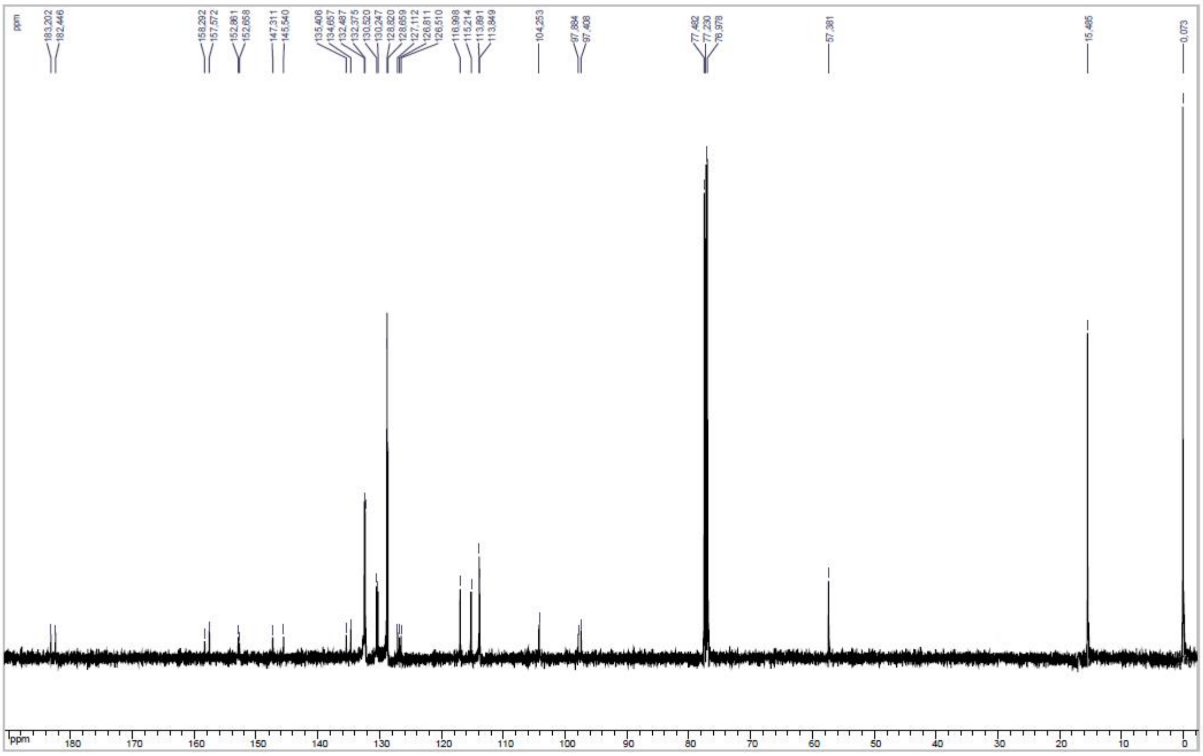

Compound **4’** ($CDCl_3$)

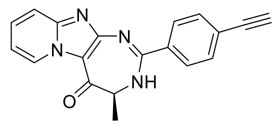

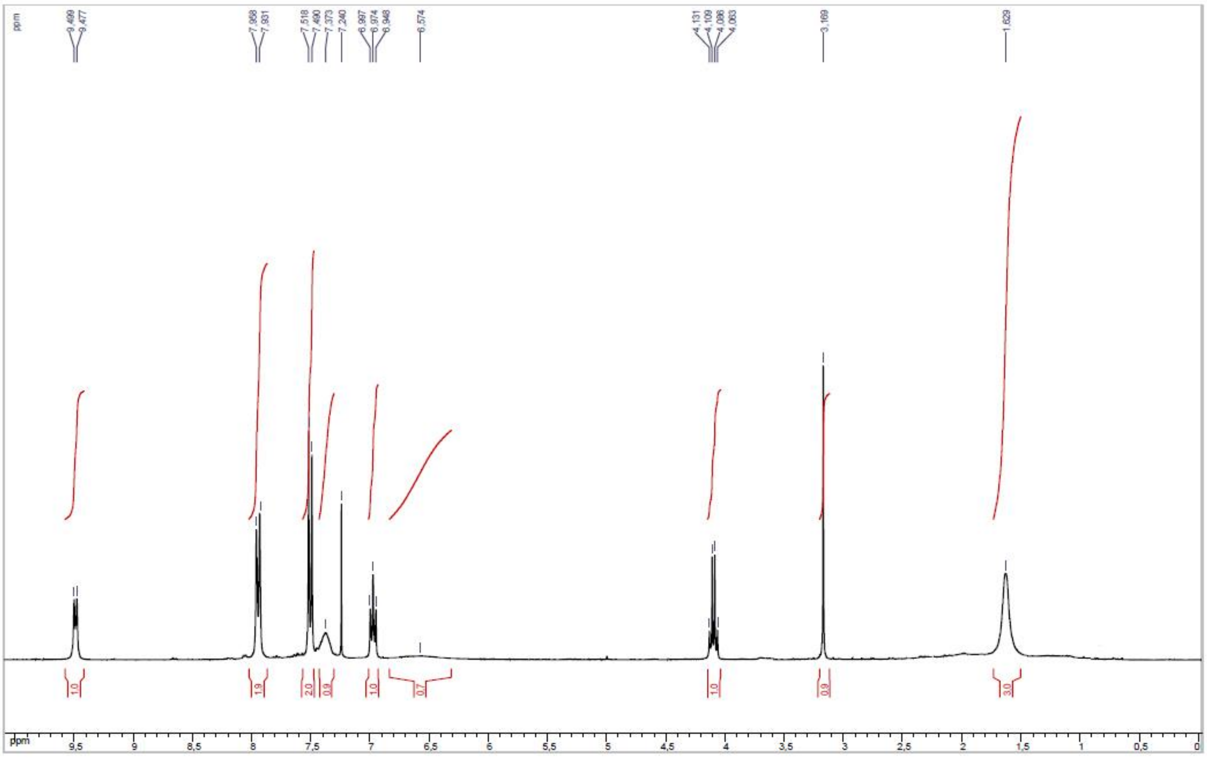

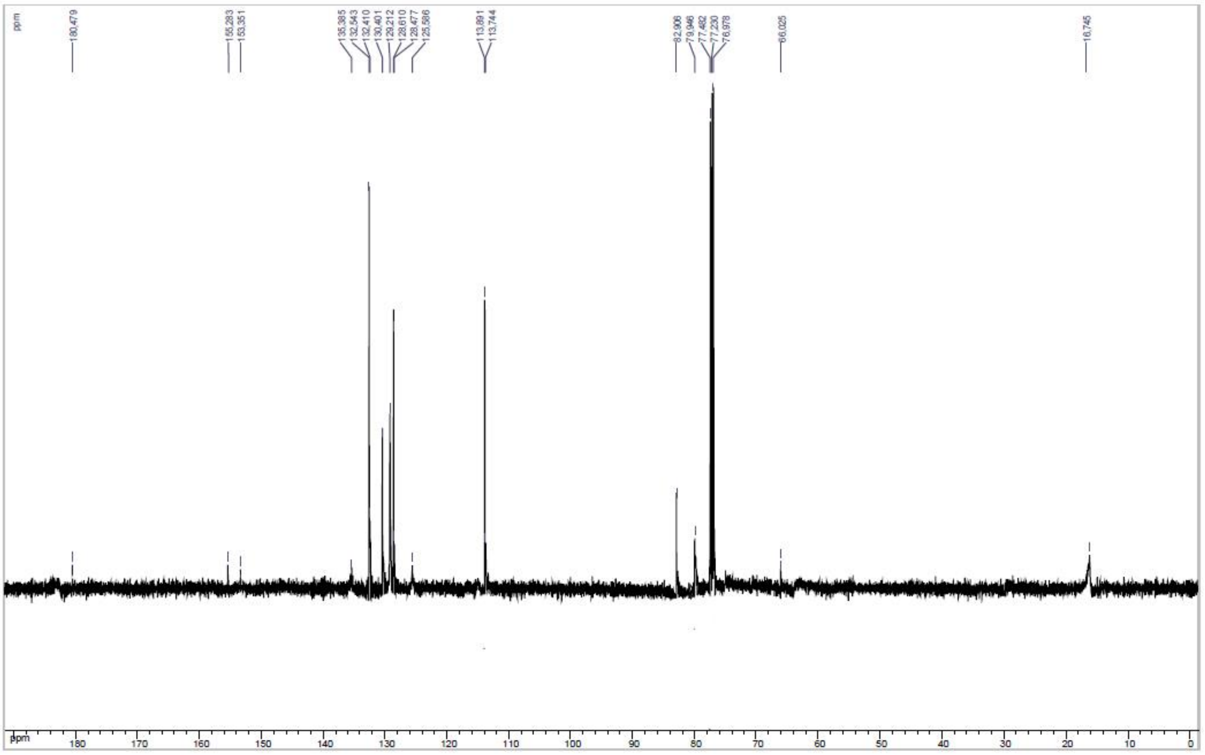

Compound **5** ($CDCl_3$)

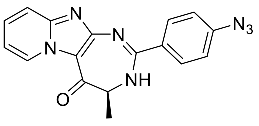

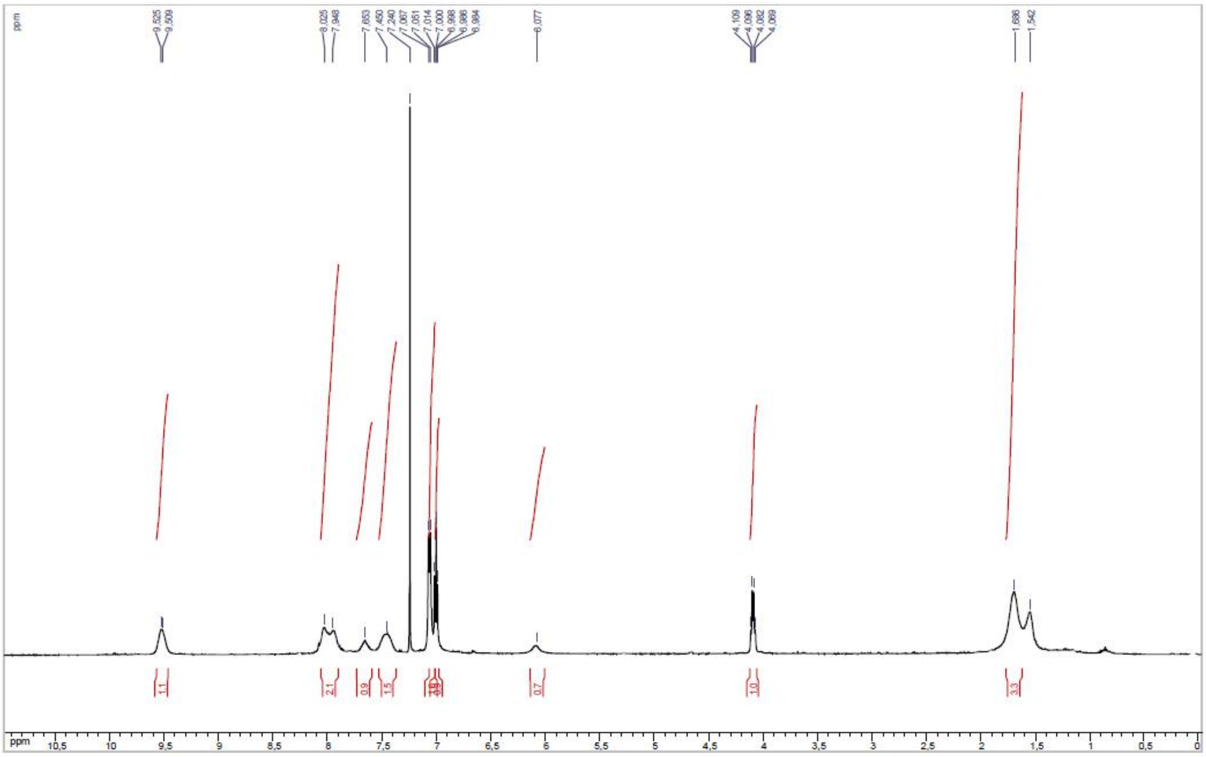

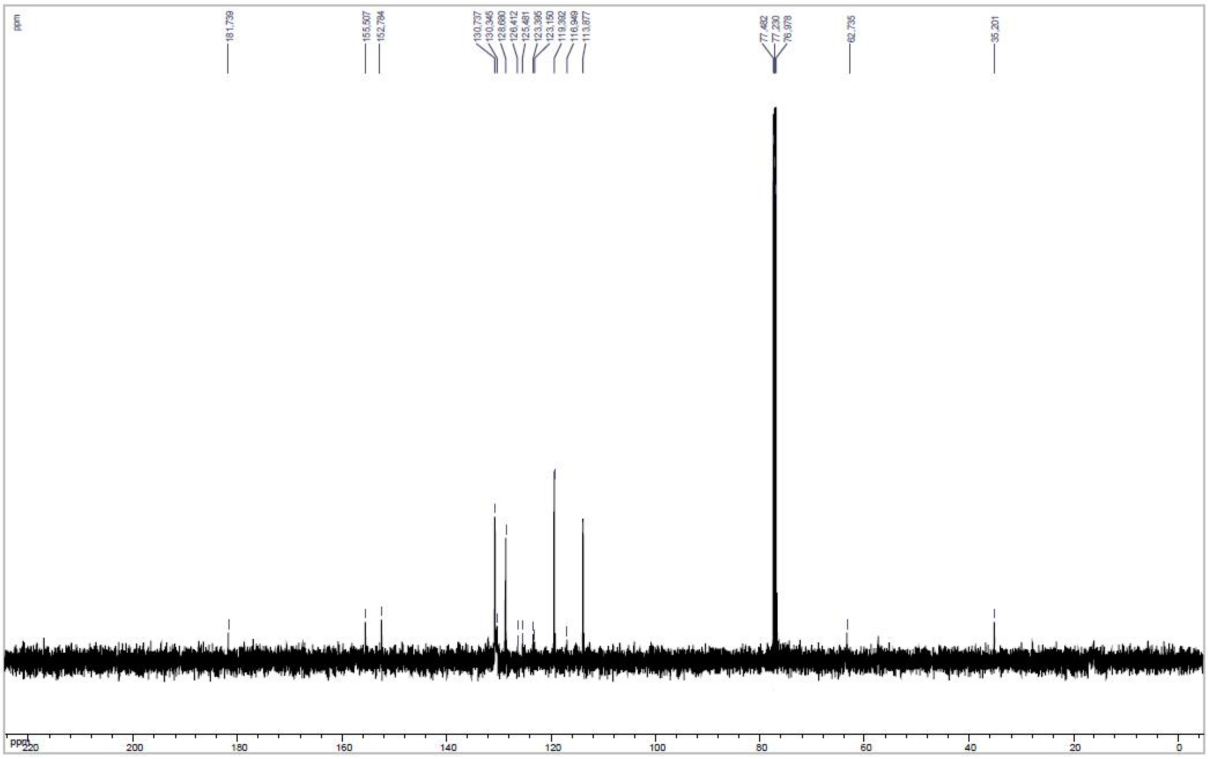

Compound **6** (DMSO d_6)

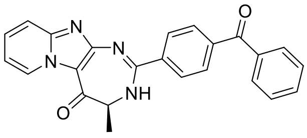

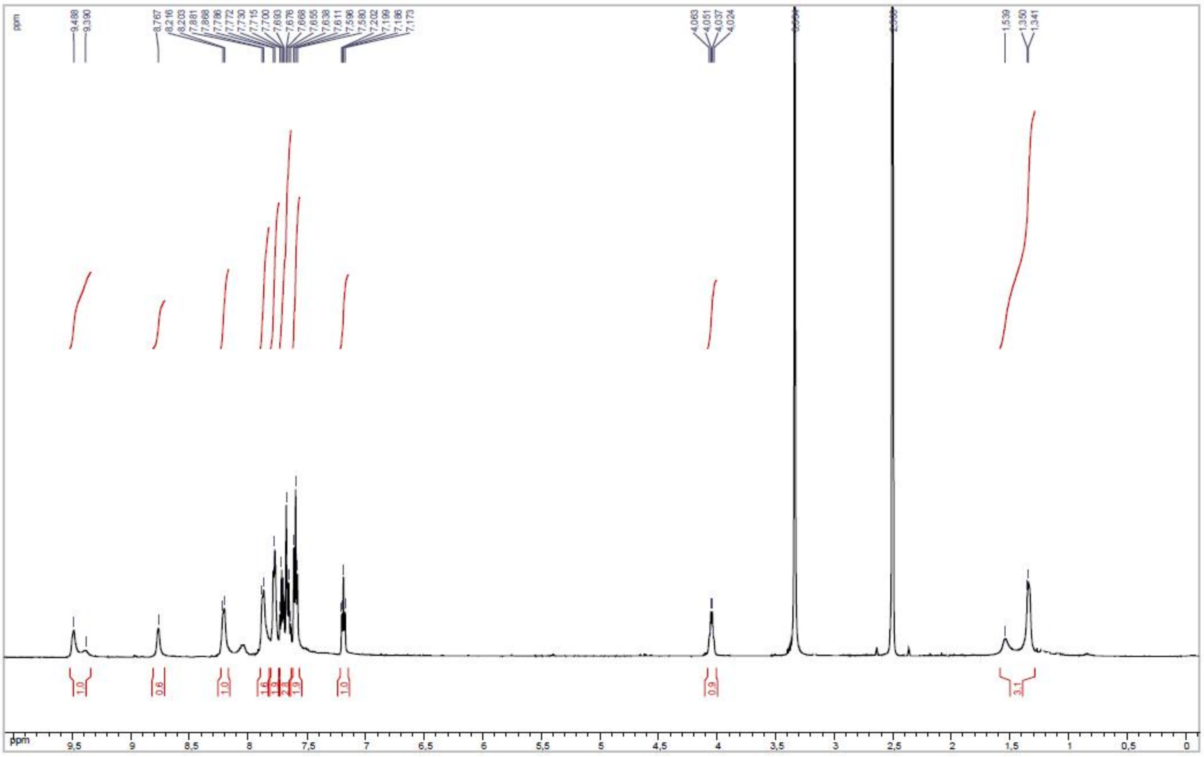

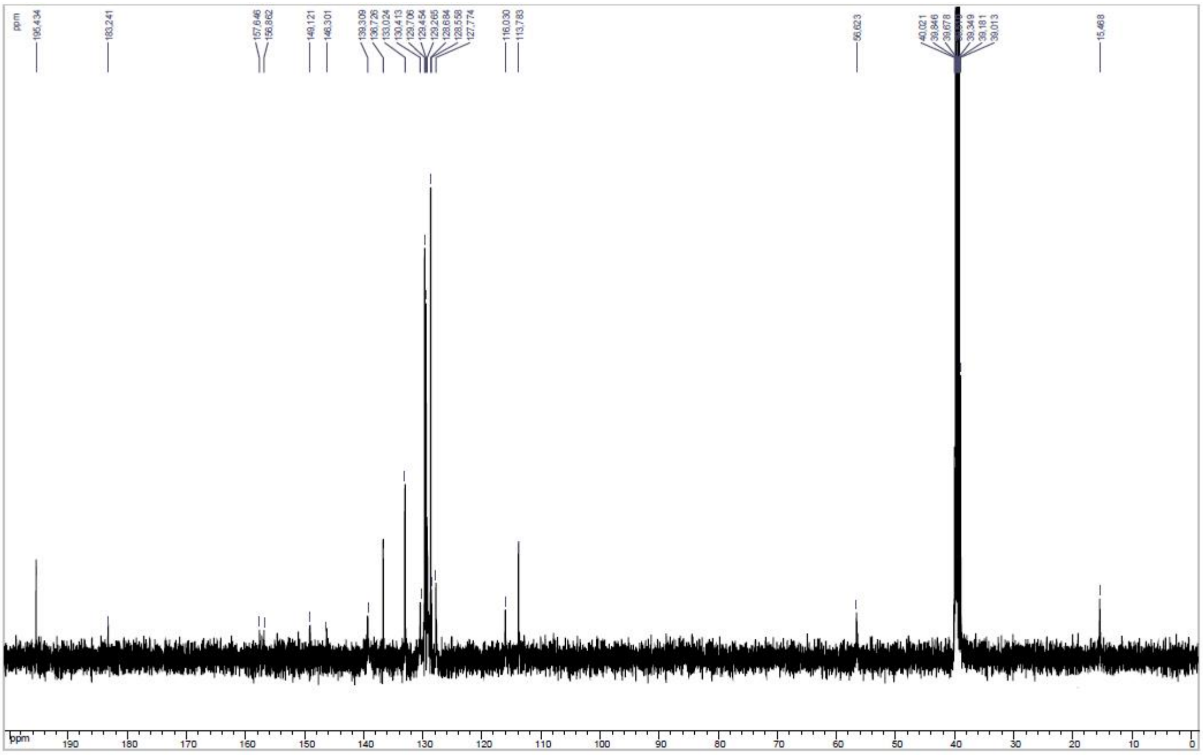

Compound **7** ($CDCl_3$)

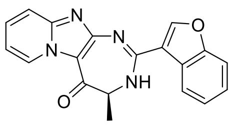

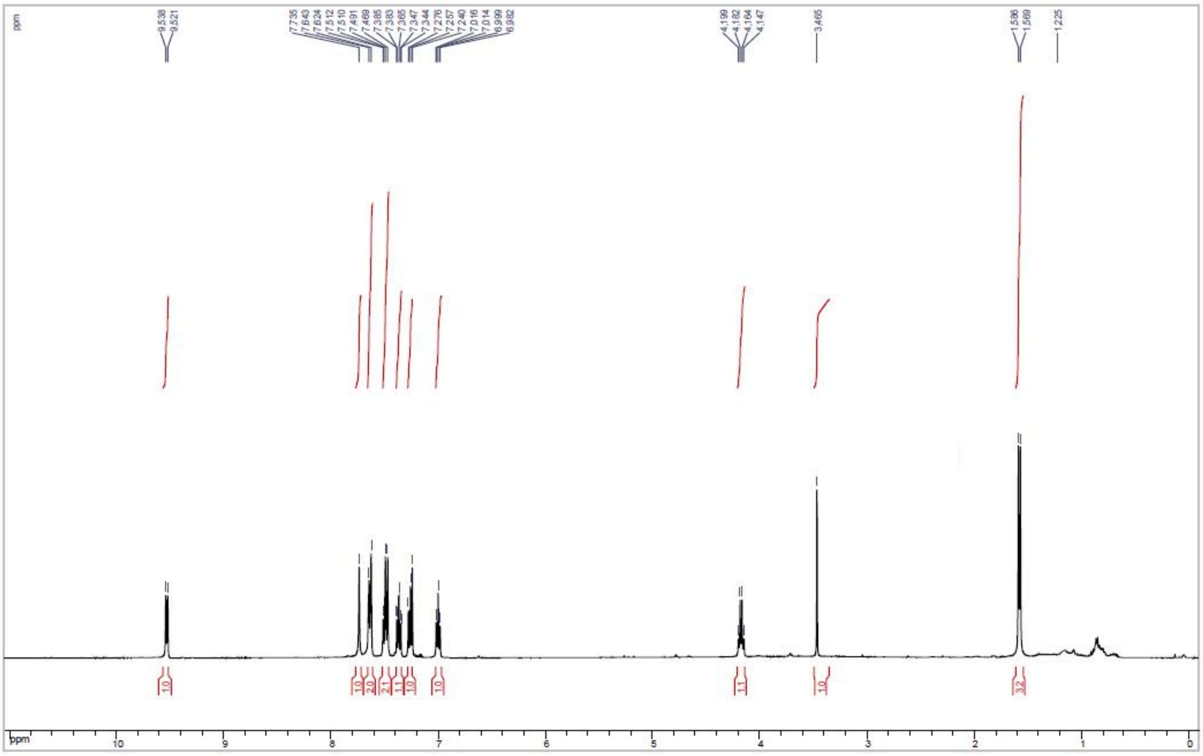

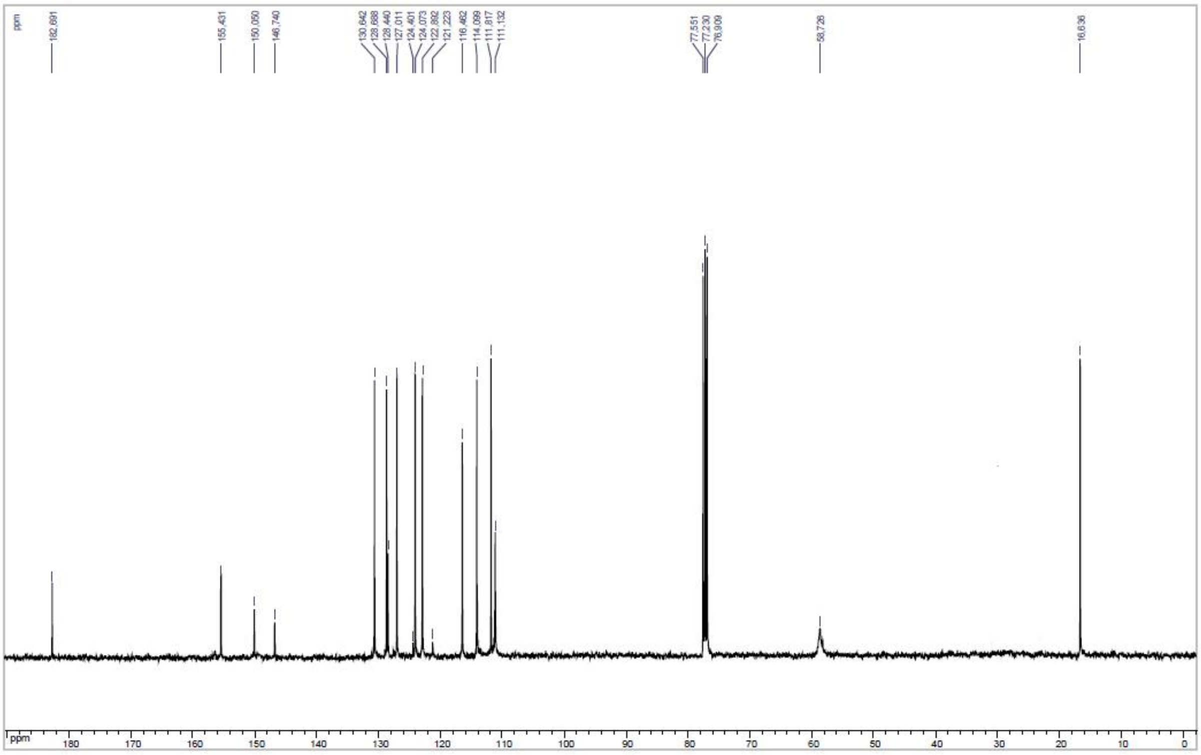

Compound **8** ($CDCl_3$)

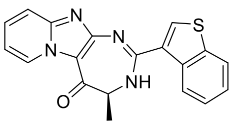

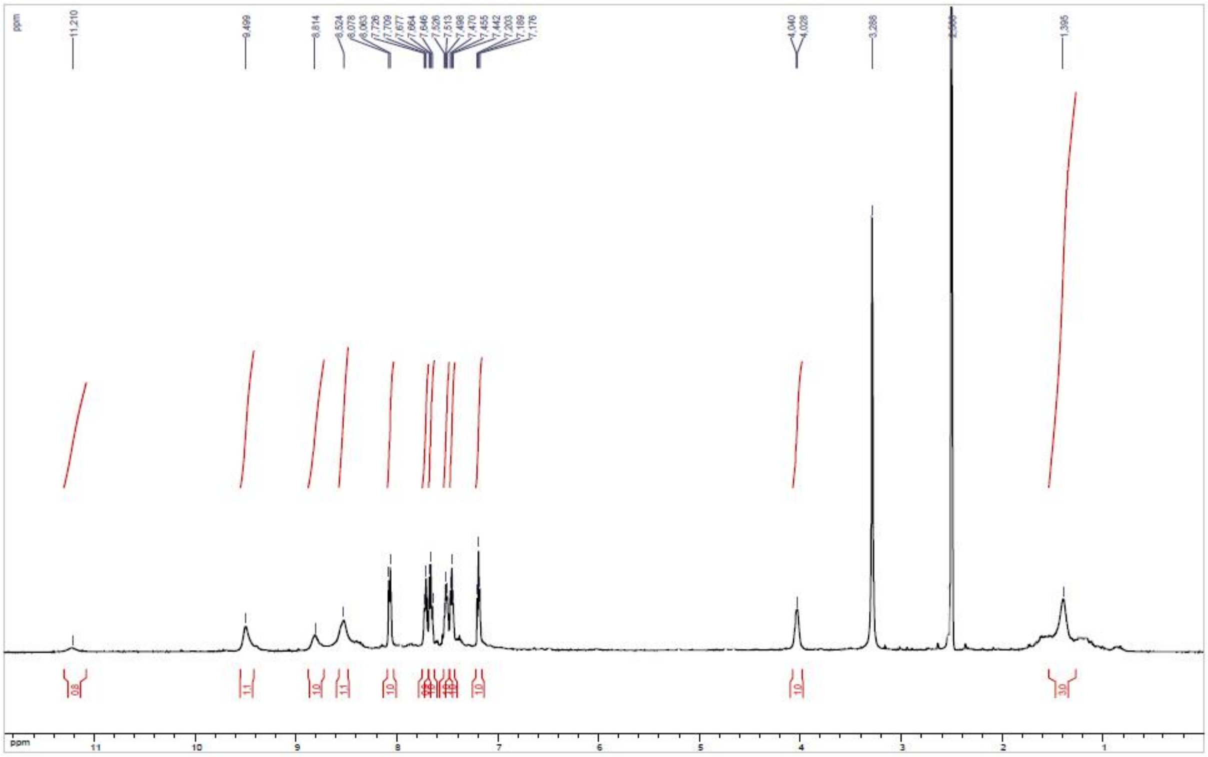

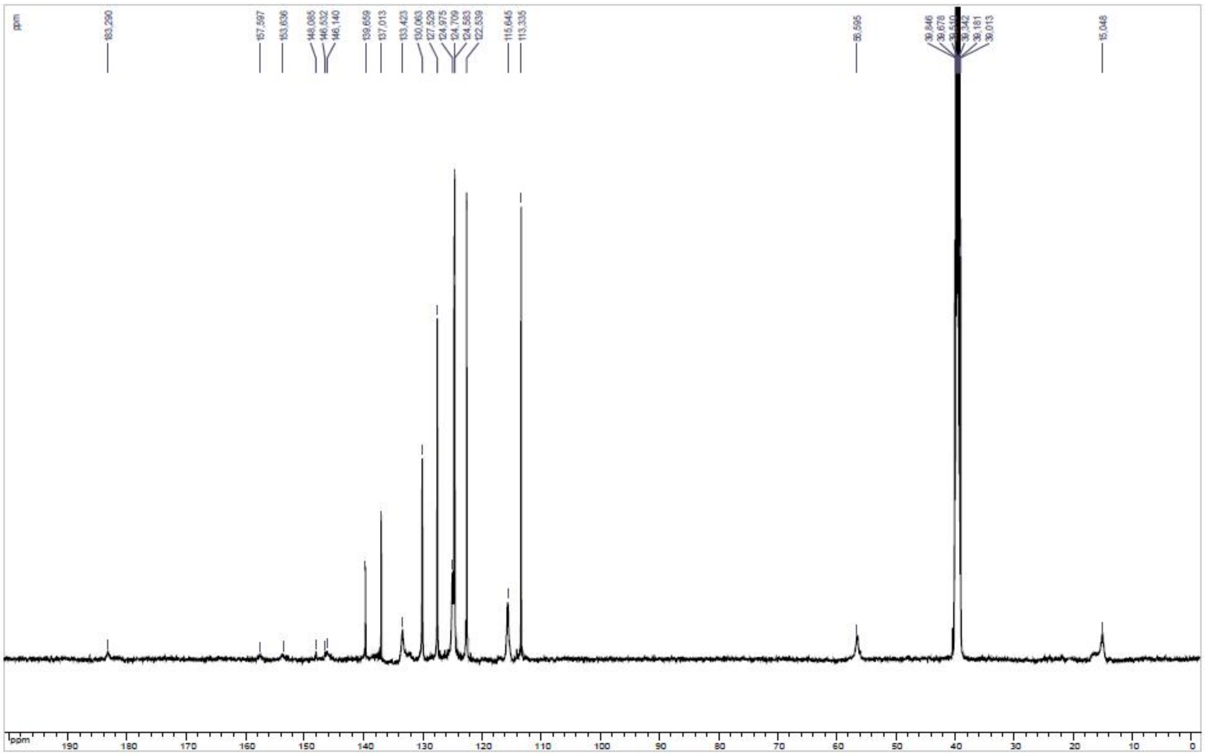

Compound **9** ($CDCl_3$)

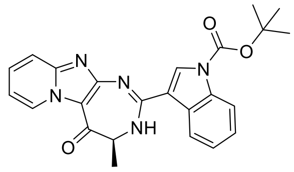

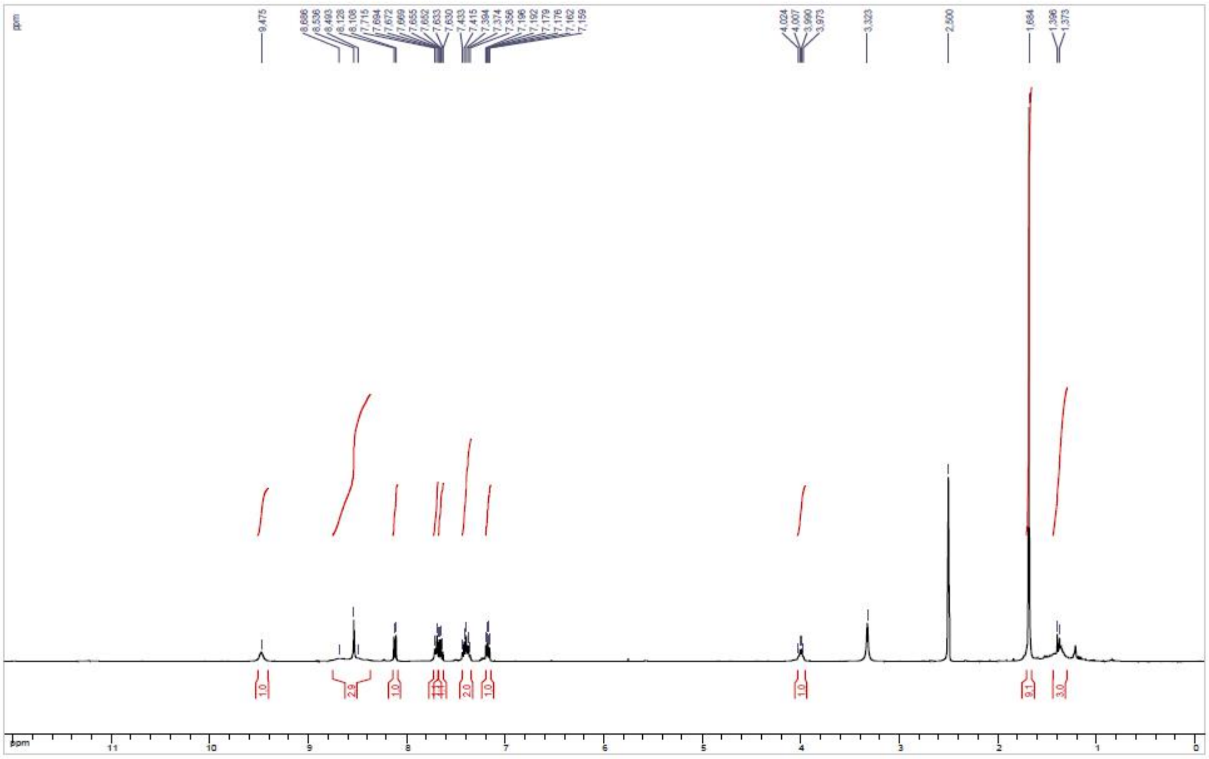

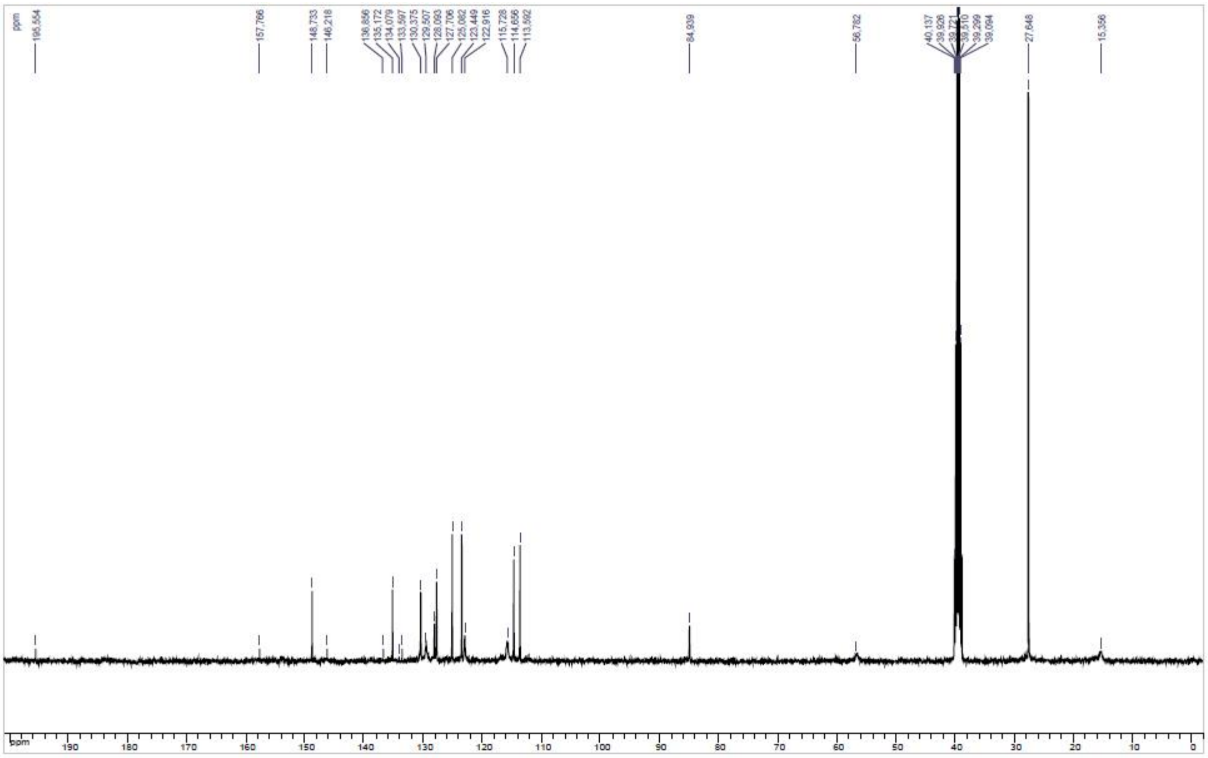

Compound **9'** ($CDCl_3$)

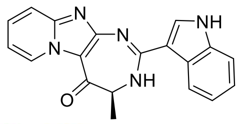

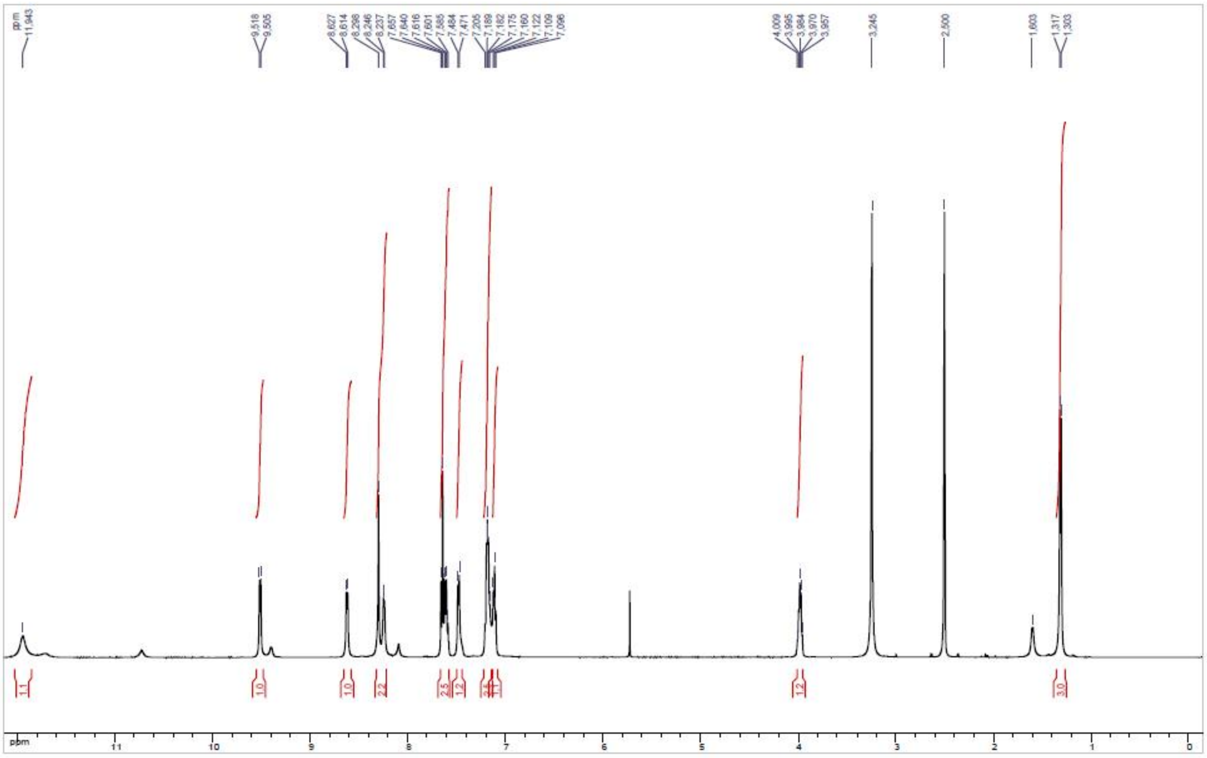

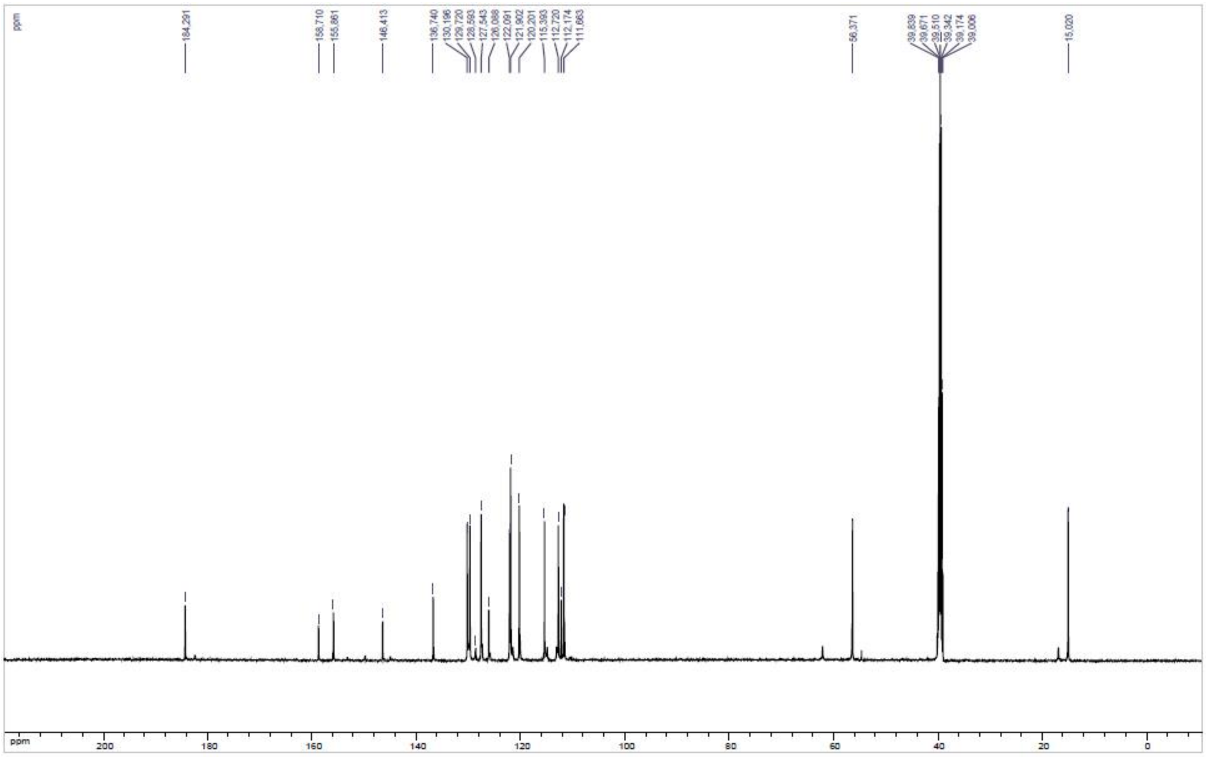

Compound **10** ($CDCl_3$)

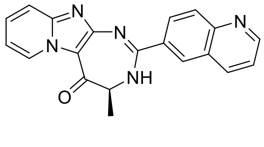

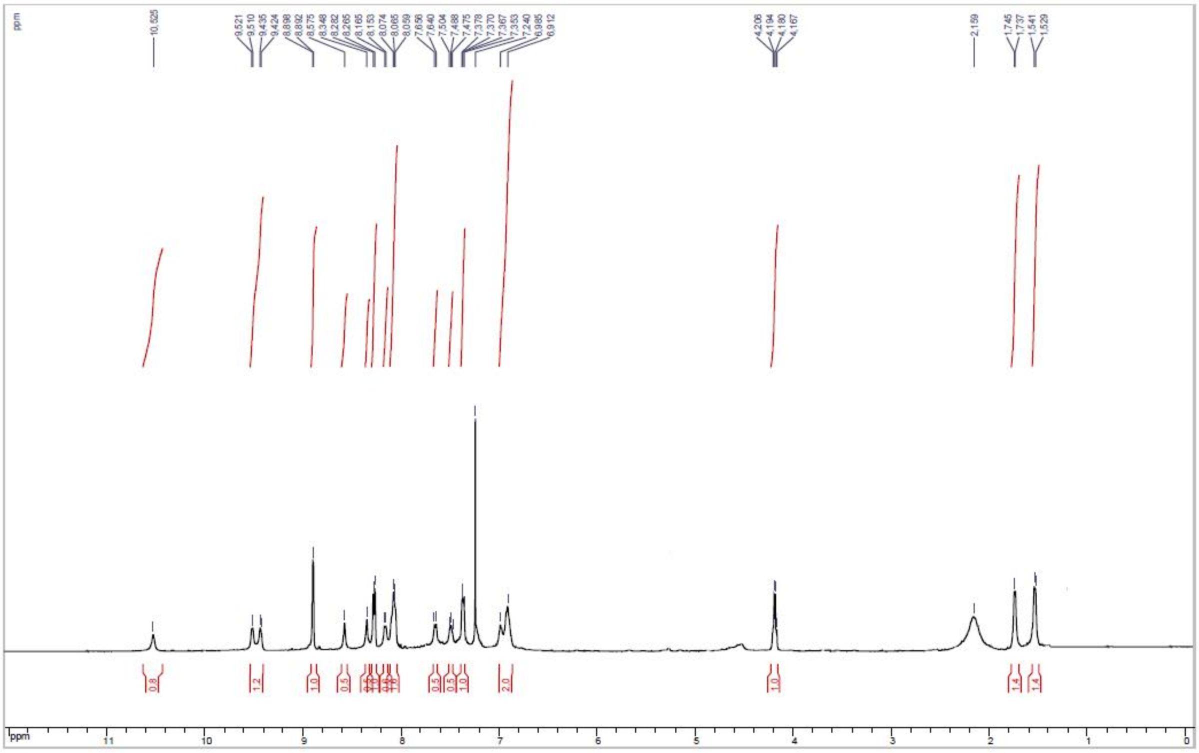

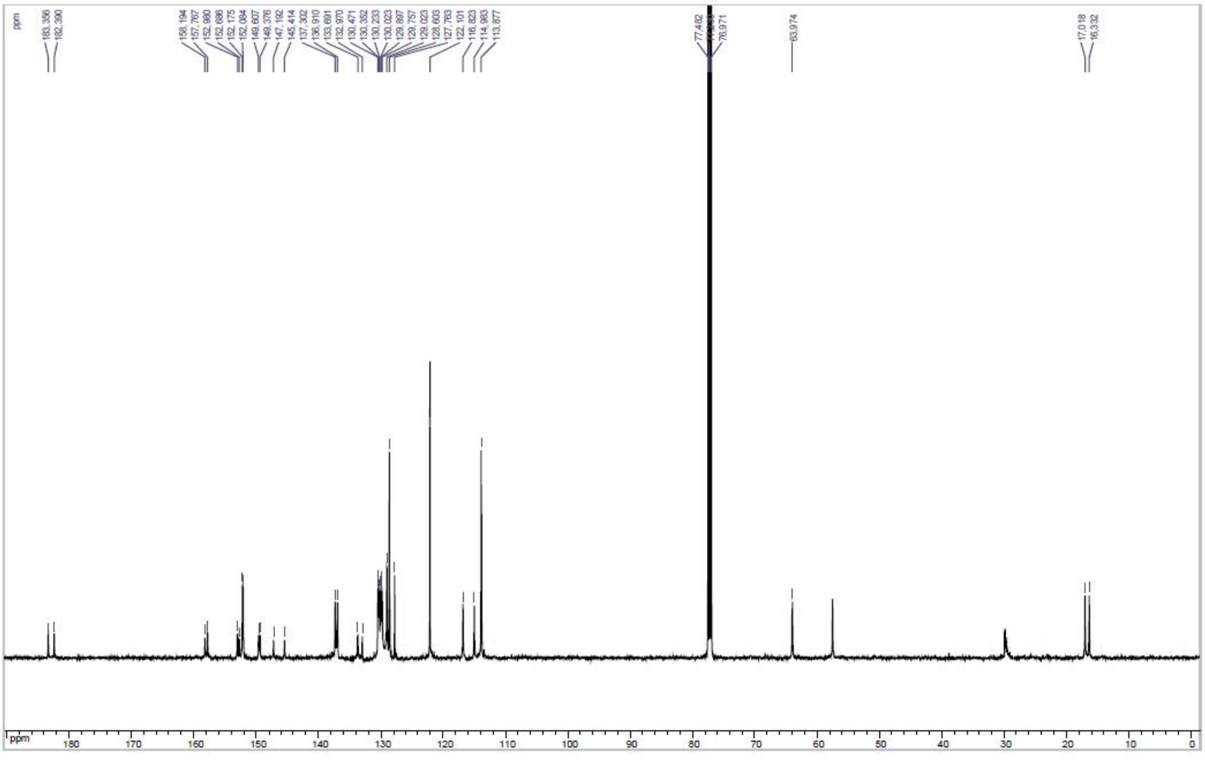

Compound **11** ($CDCl_3$)

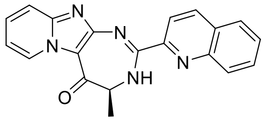

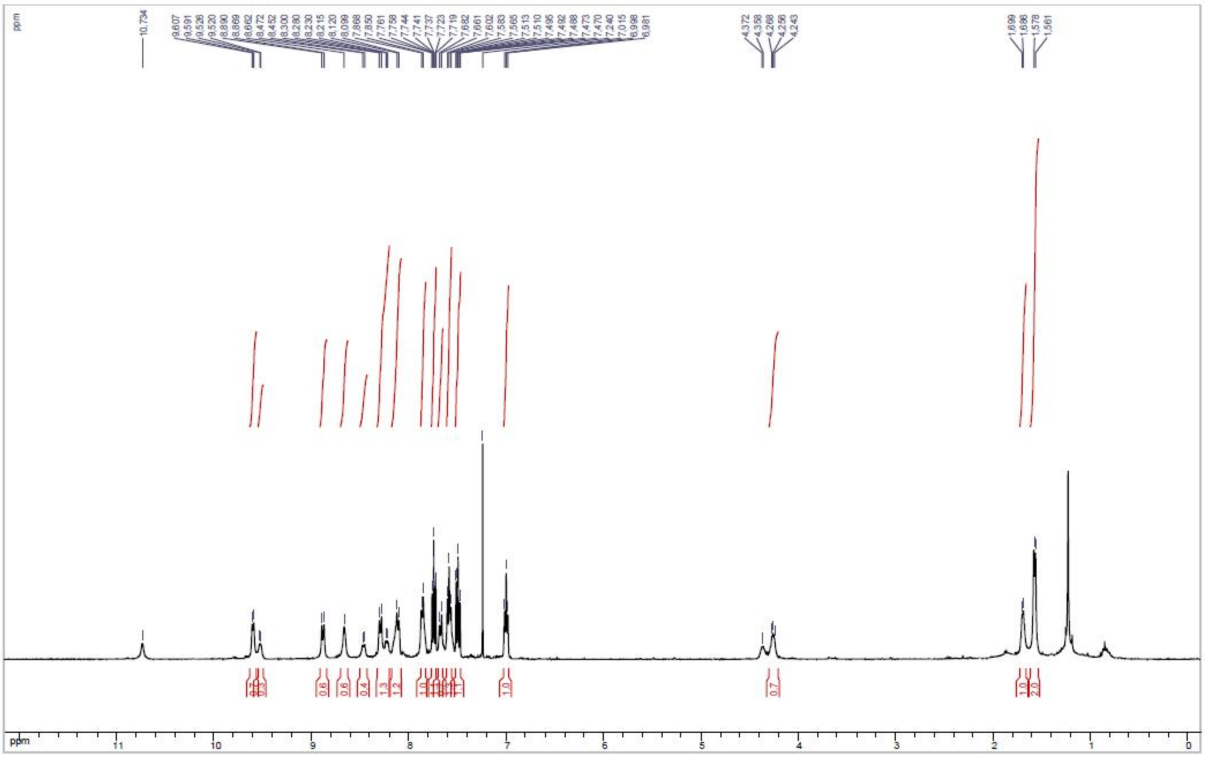

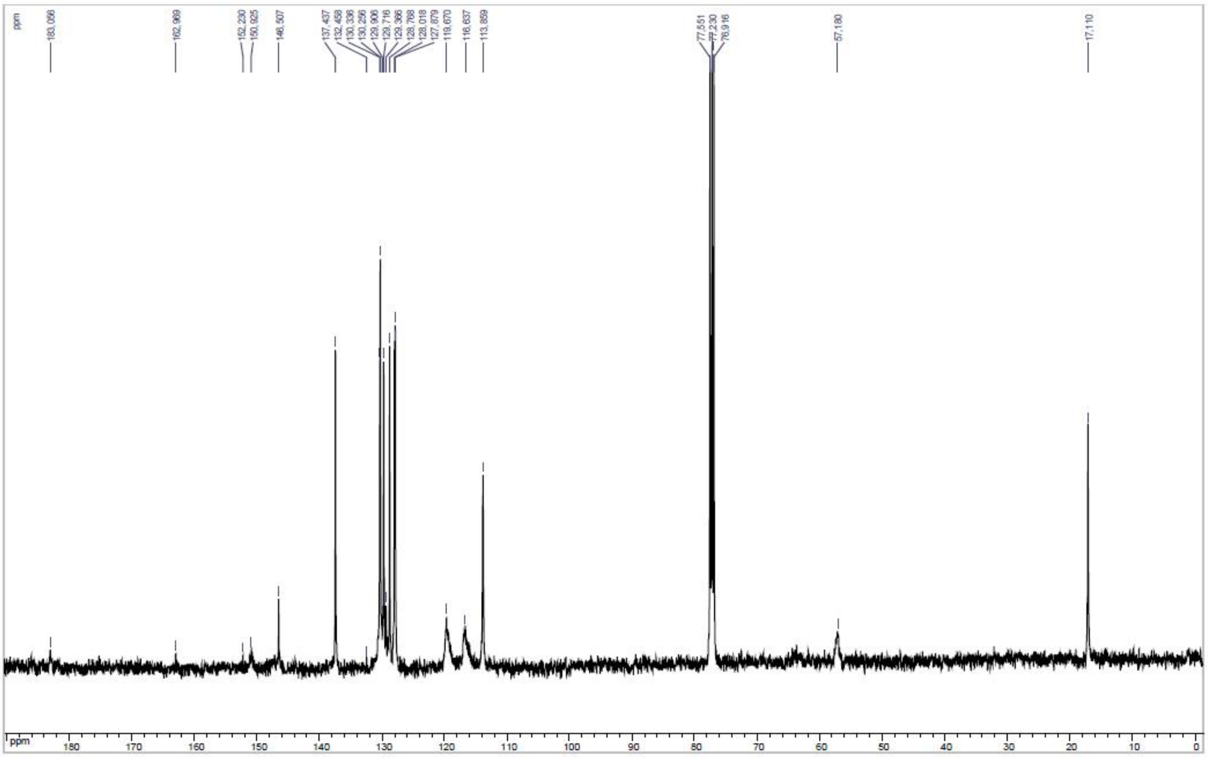

Compound **12** (DMSO d_6)

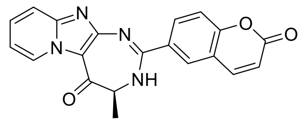

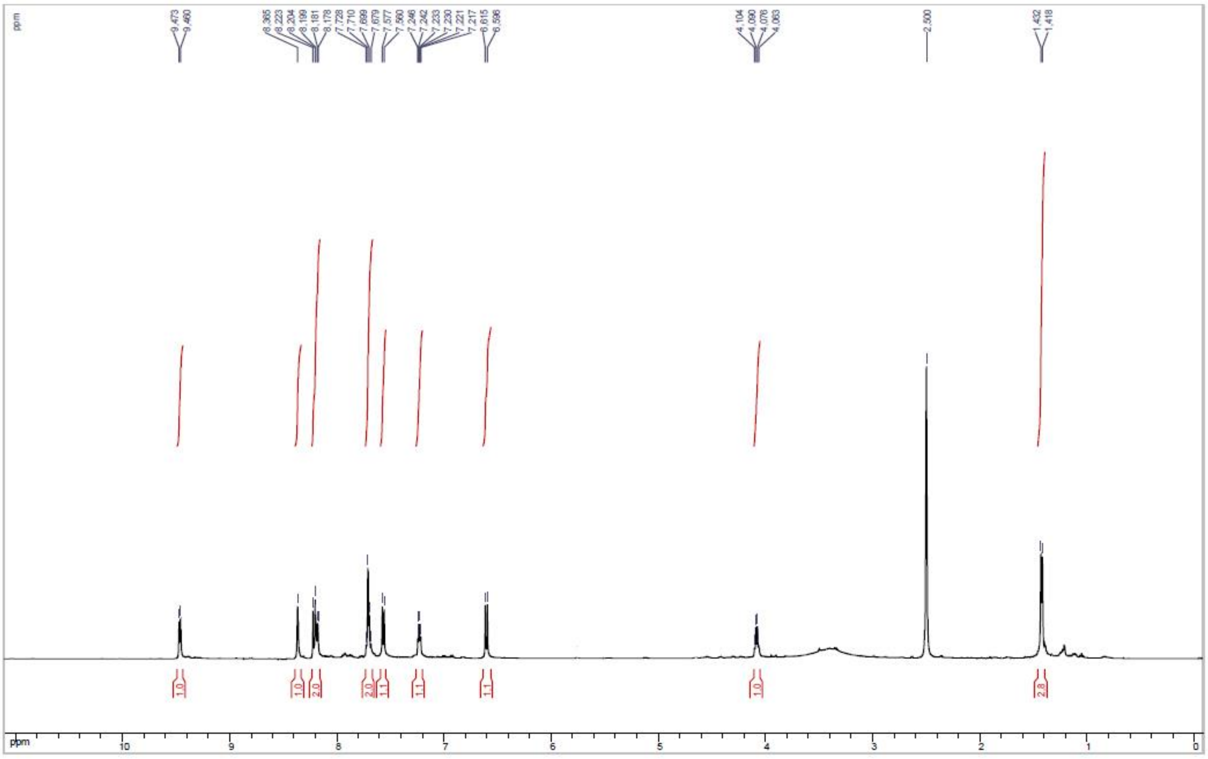

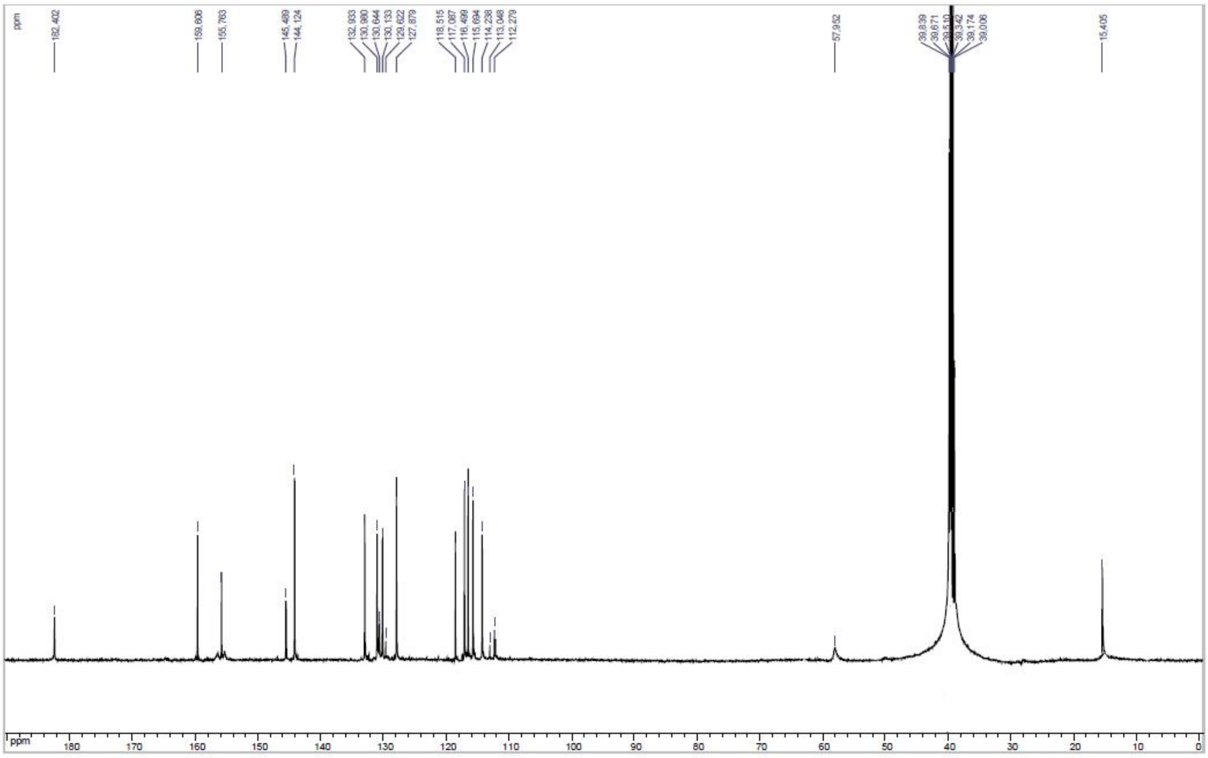

Compound **13** ($CDCl_3$)

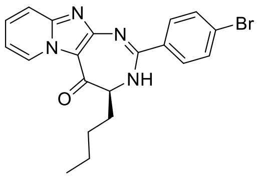

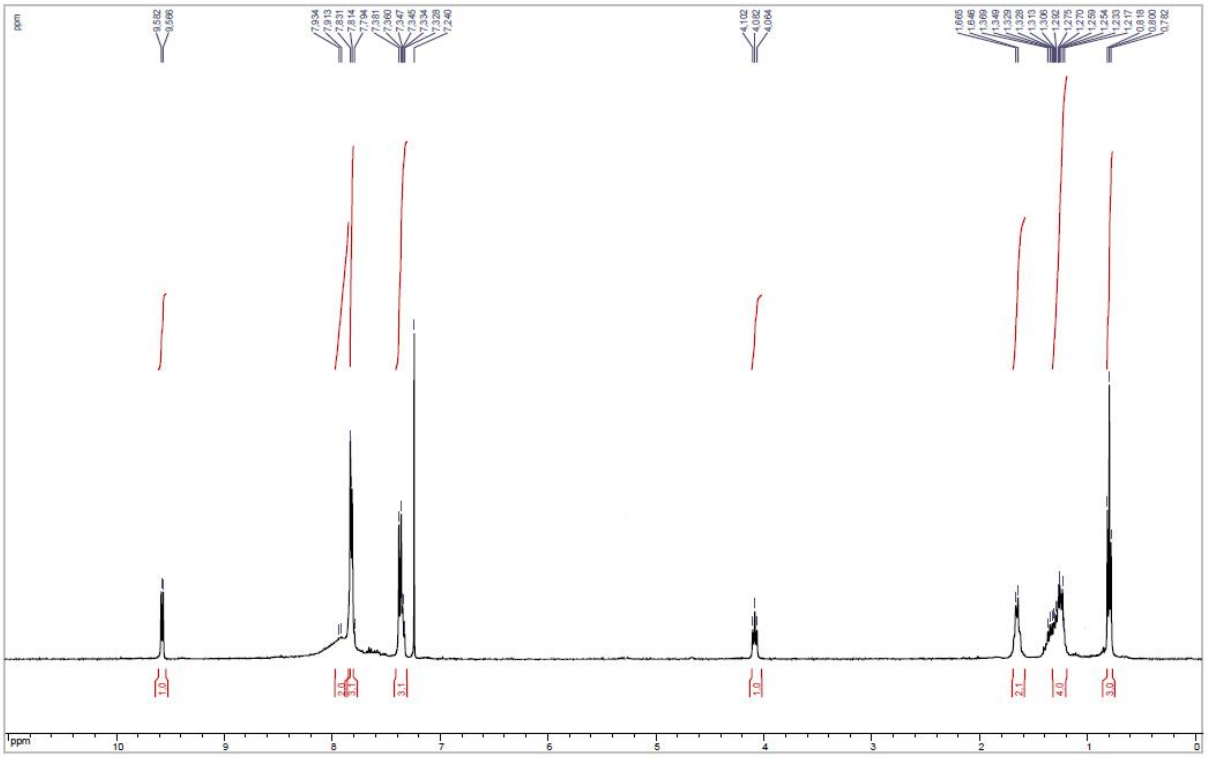

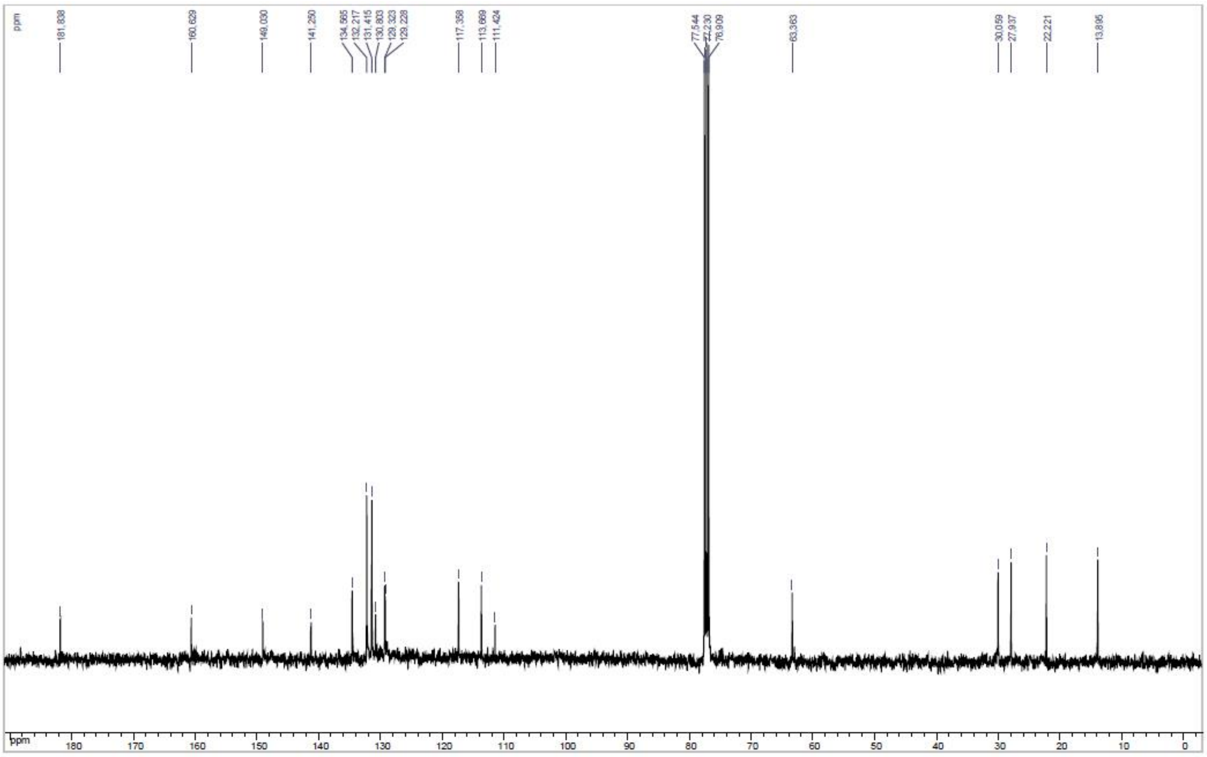

Compound **15** ($CDCl_3$)

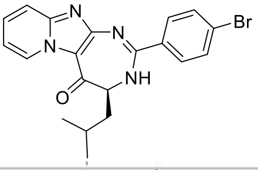

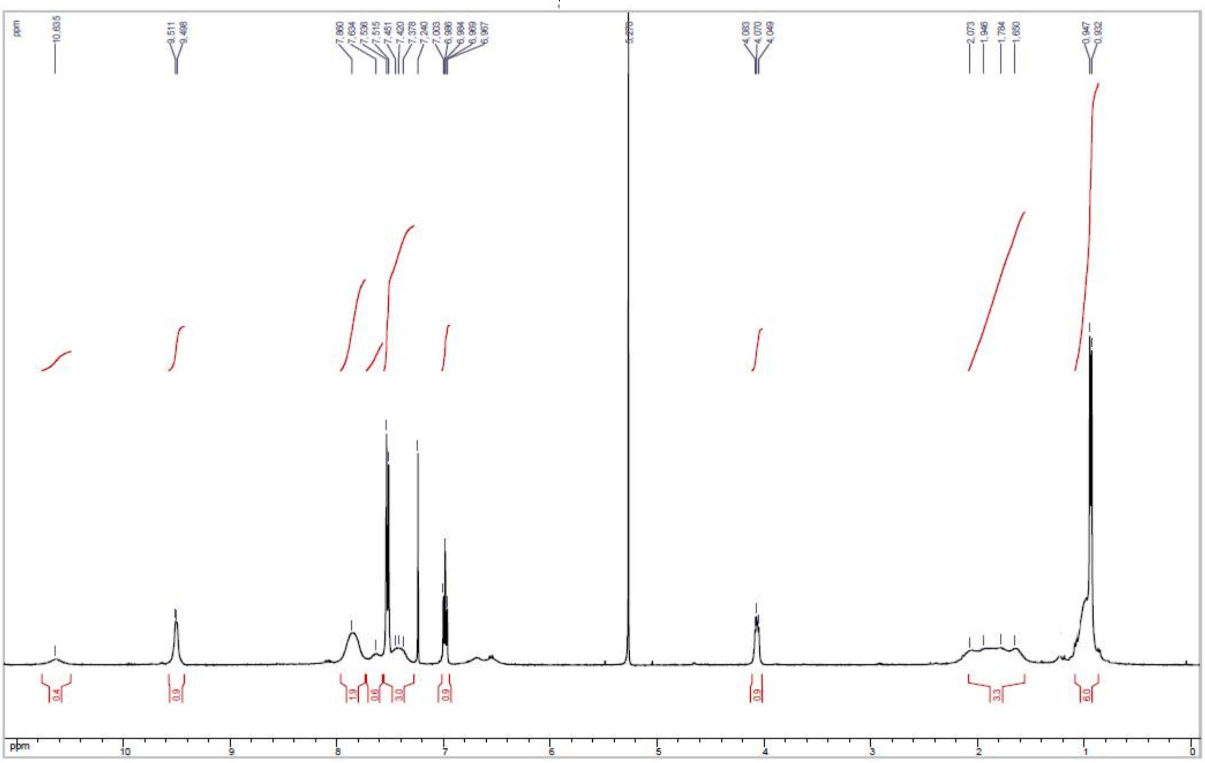

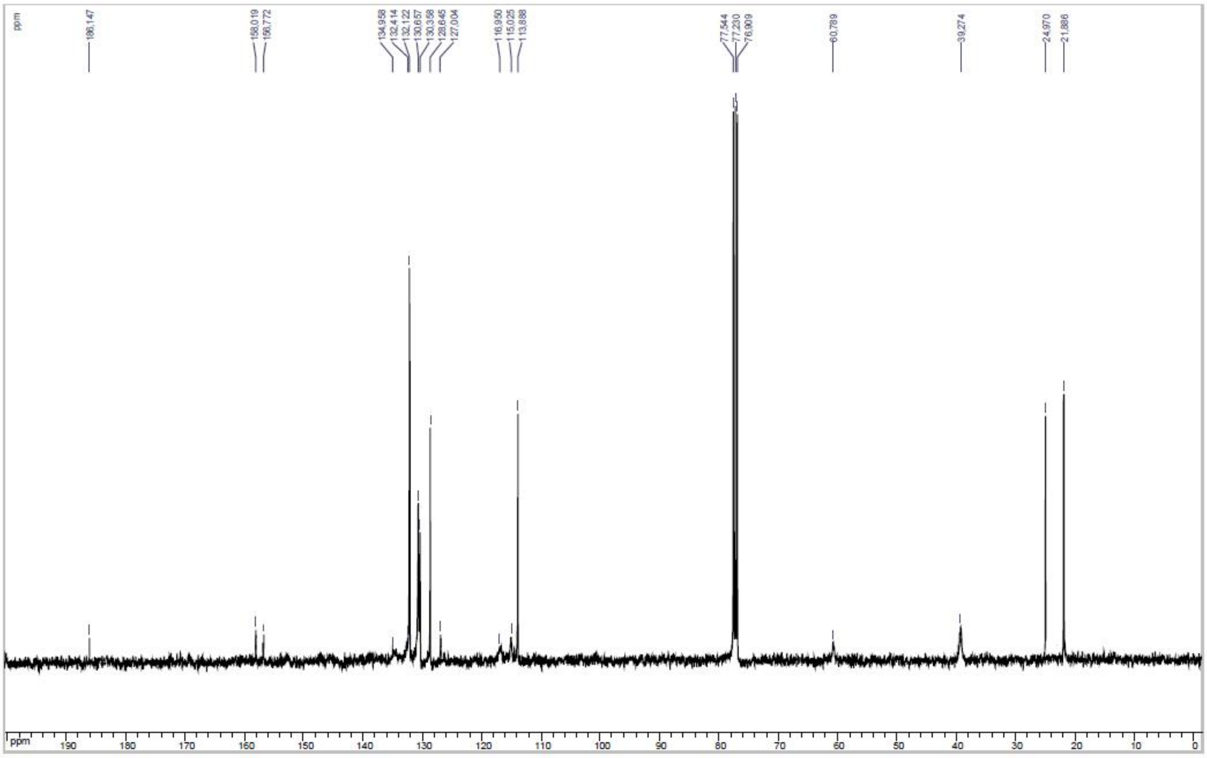

Compound **16** ($CDCl_3$)

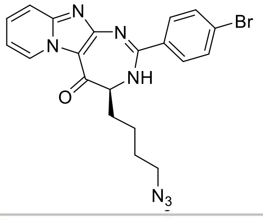

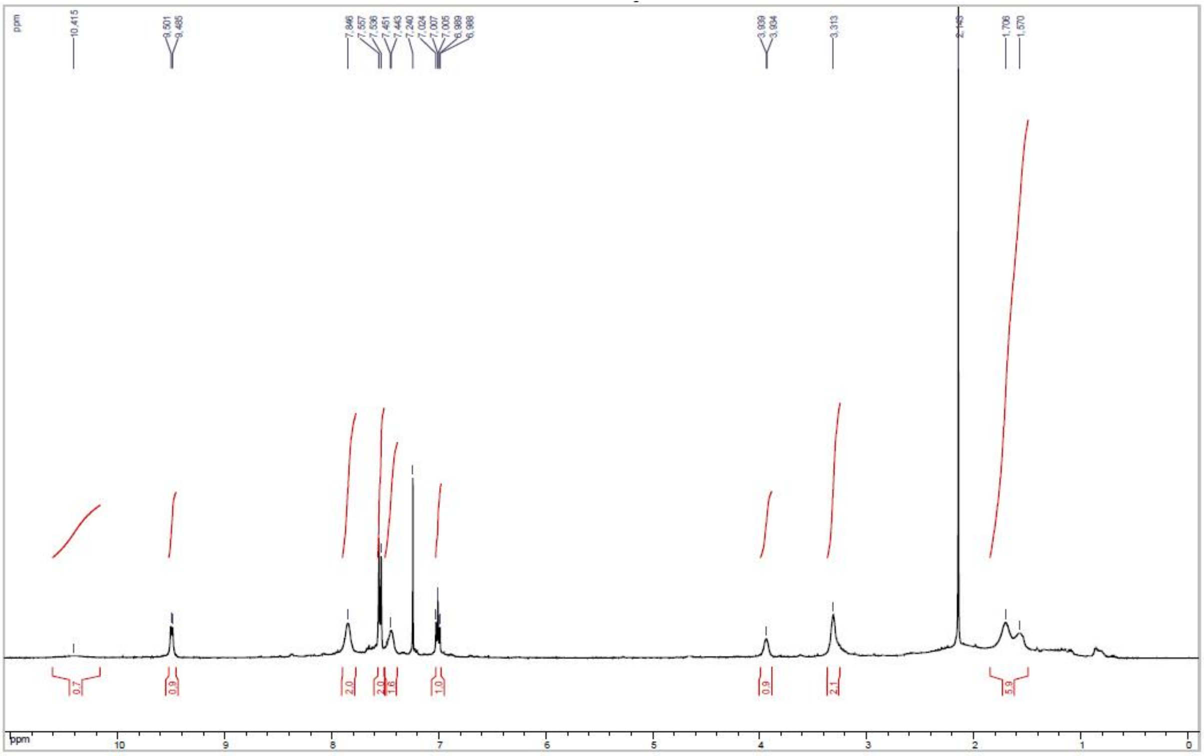

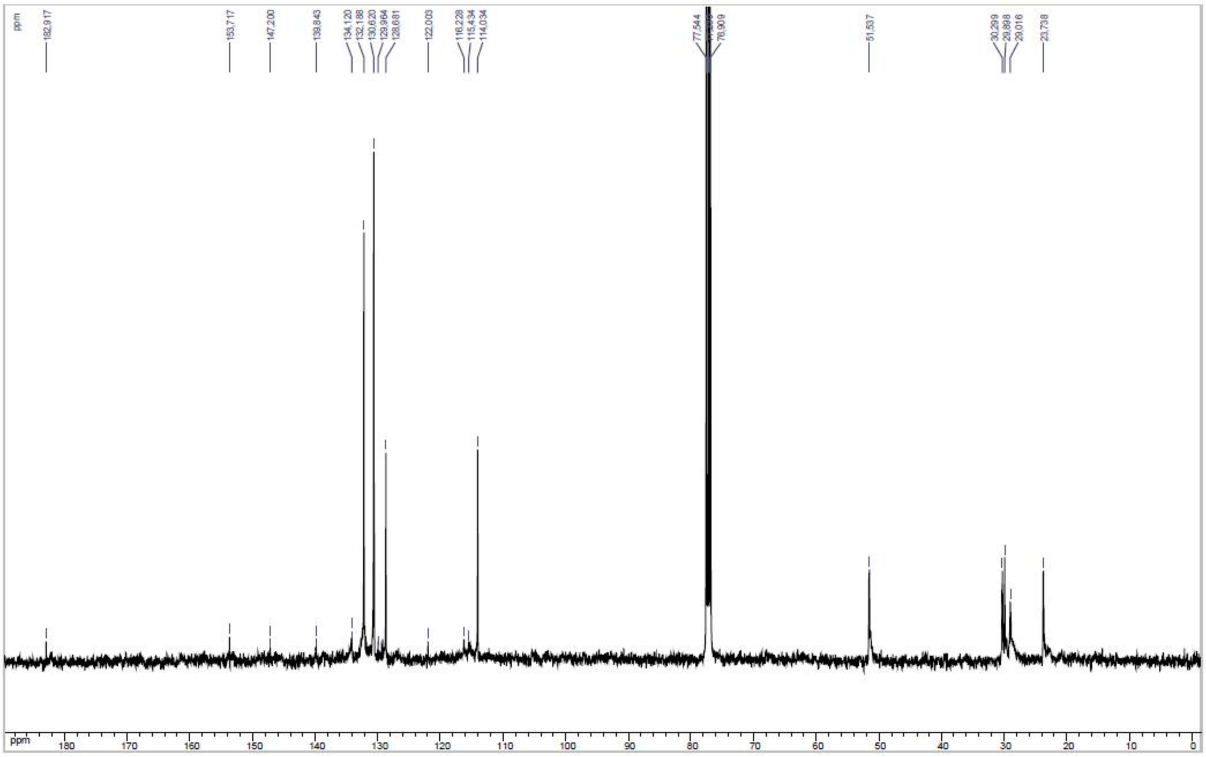

Compound **18** ($CDCl_3$)

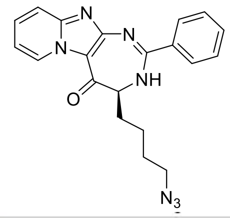

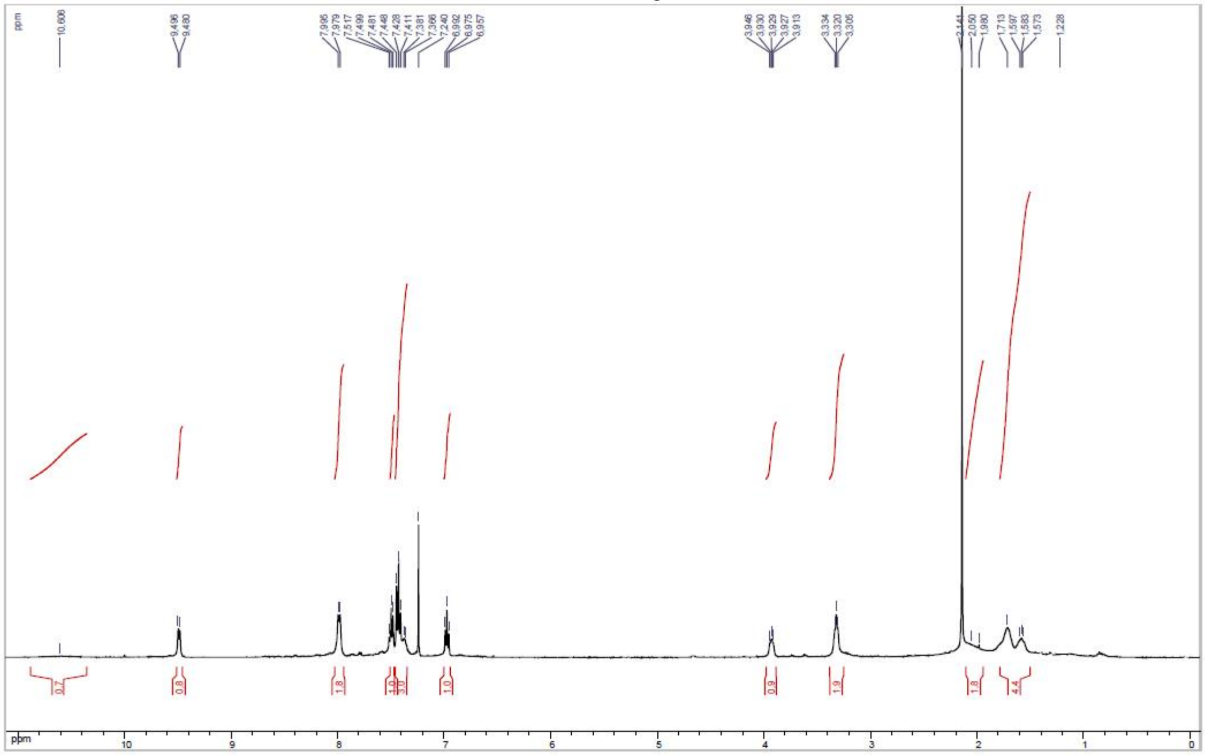

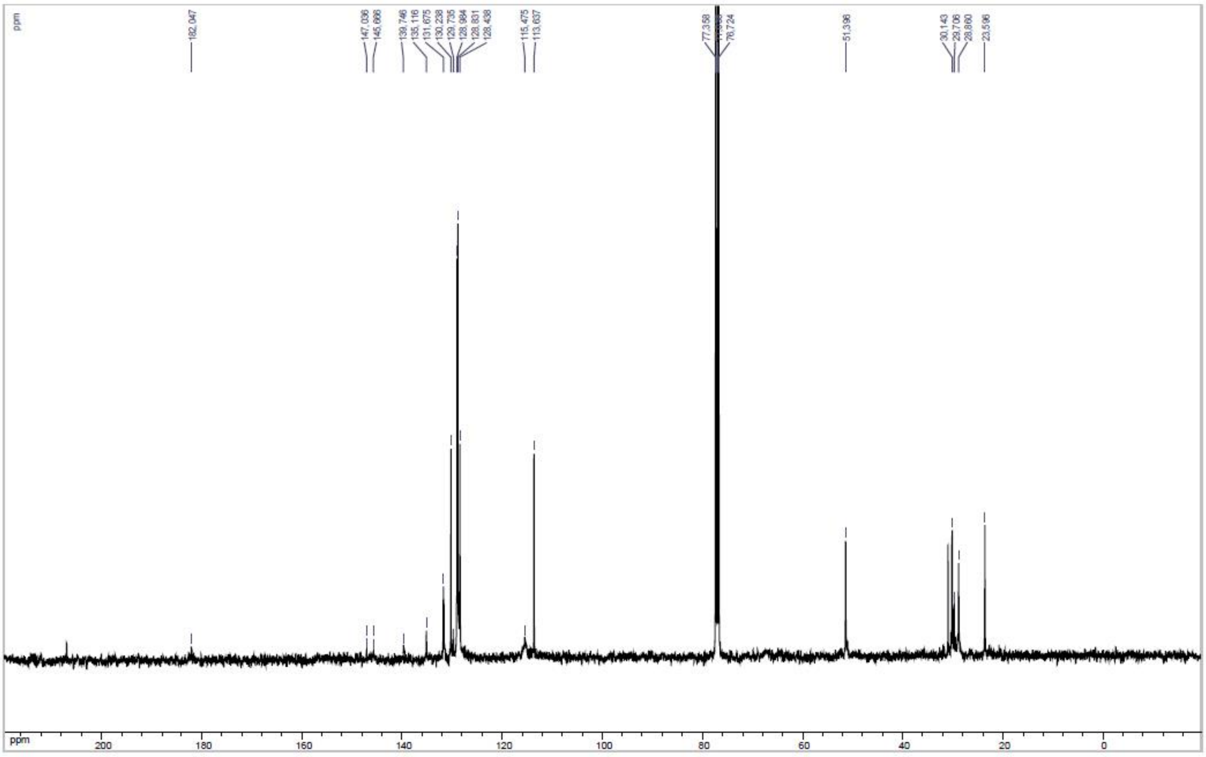

Compound **19** ($CDCl_3$)

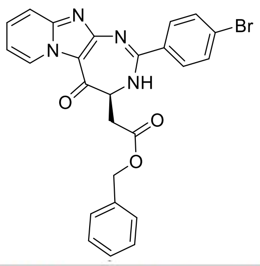

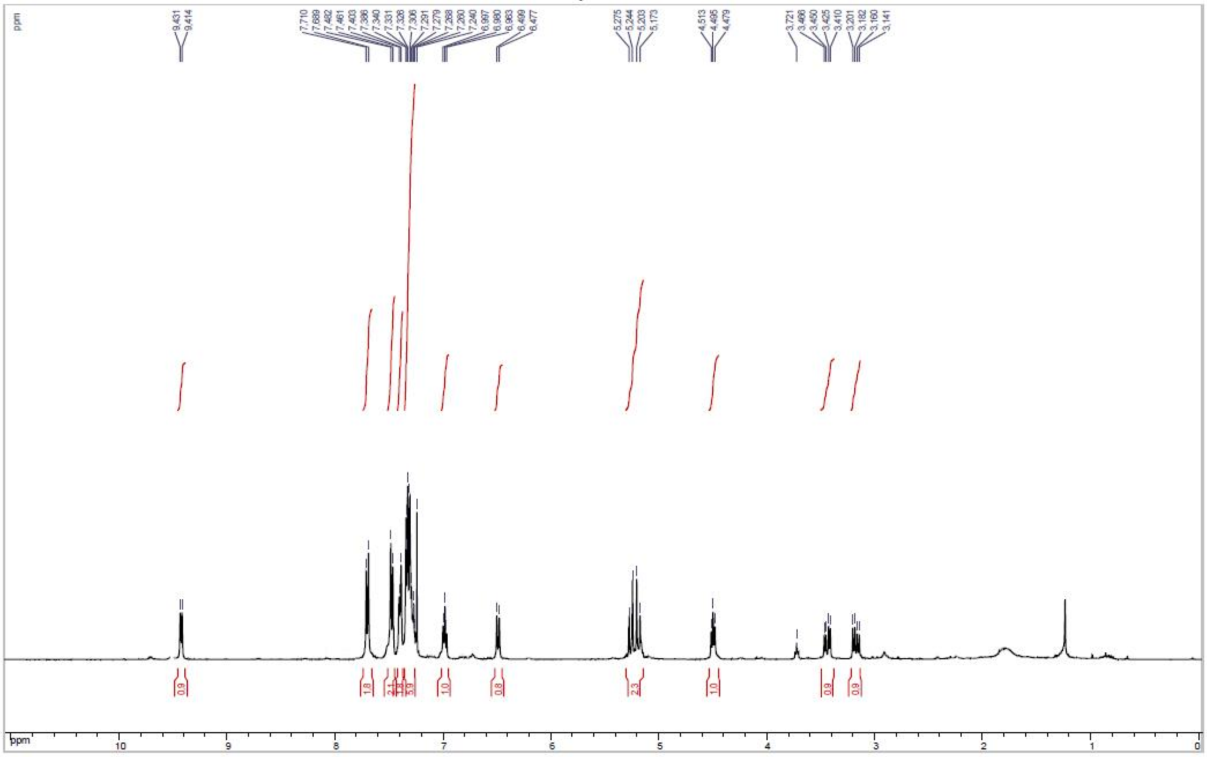

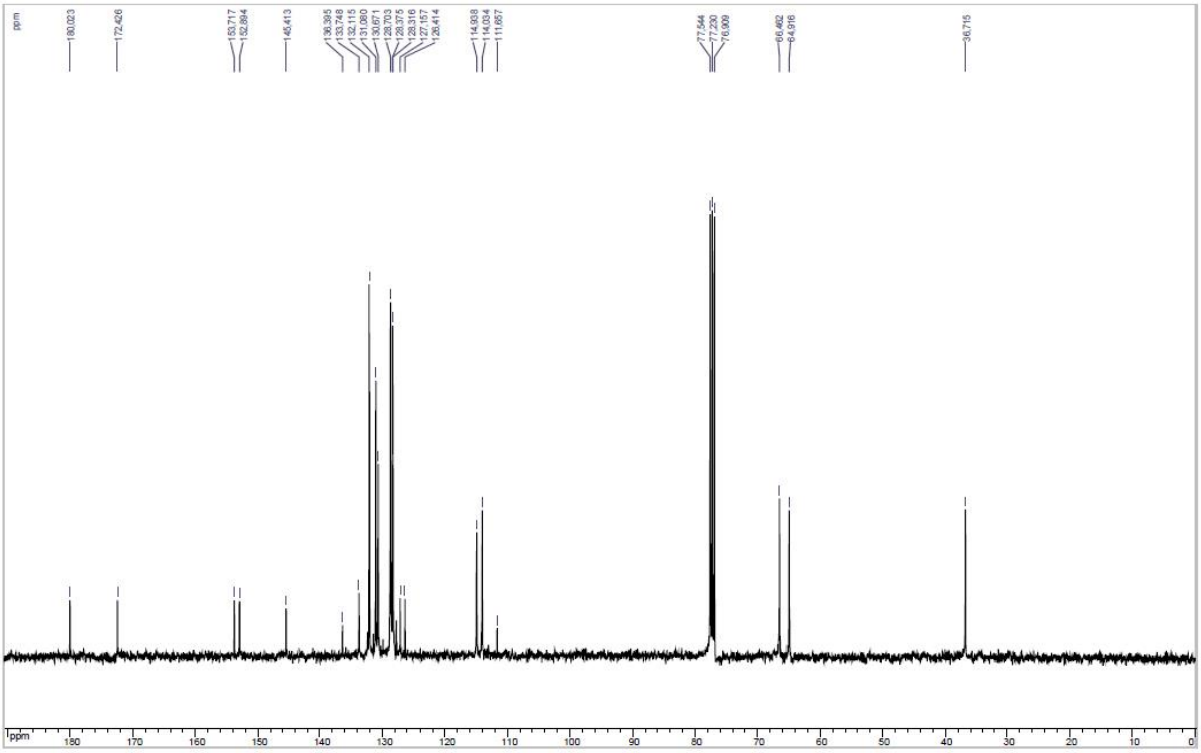

Compound **20** ($CDCl_3$)

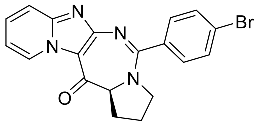

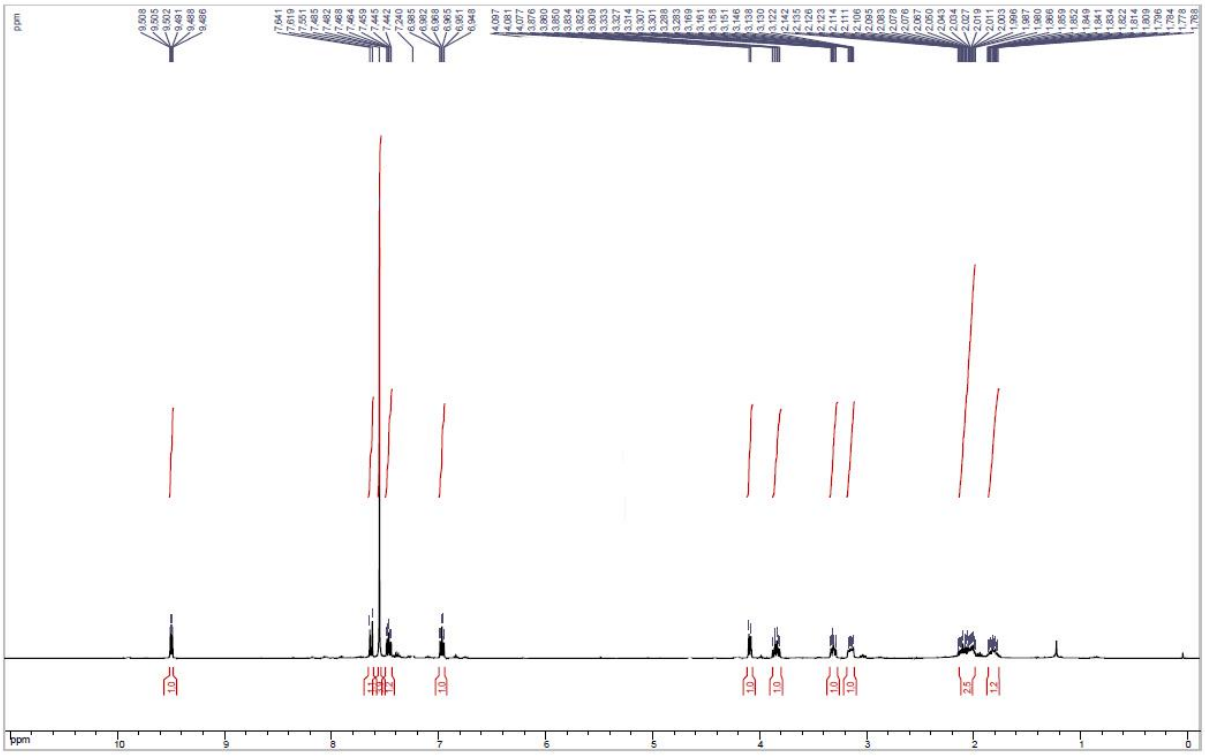

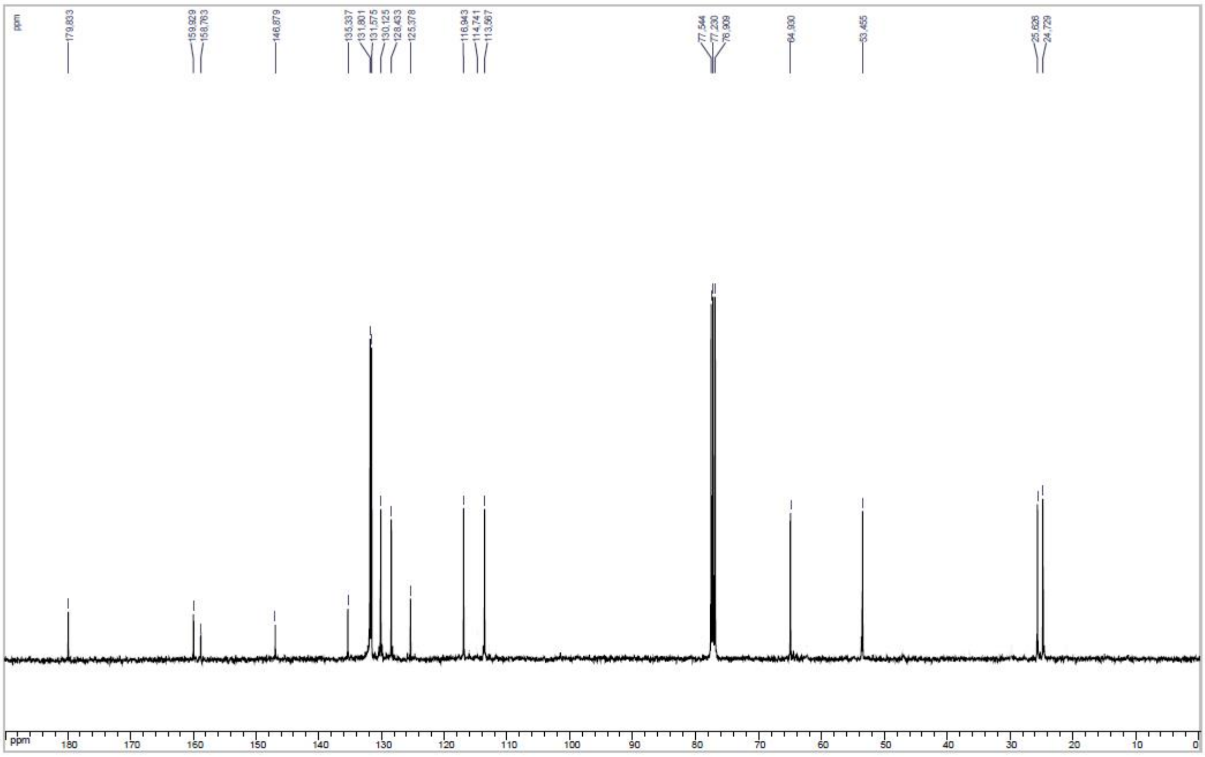

Compound **21** (DMSO d_6)

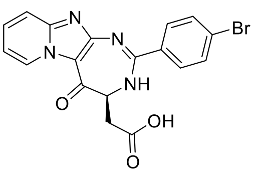

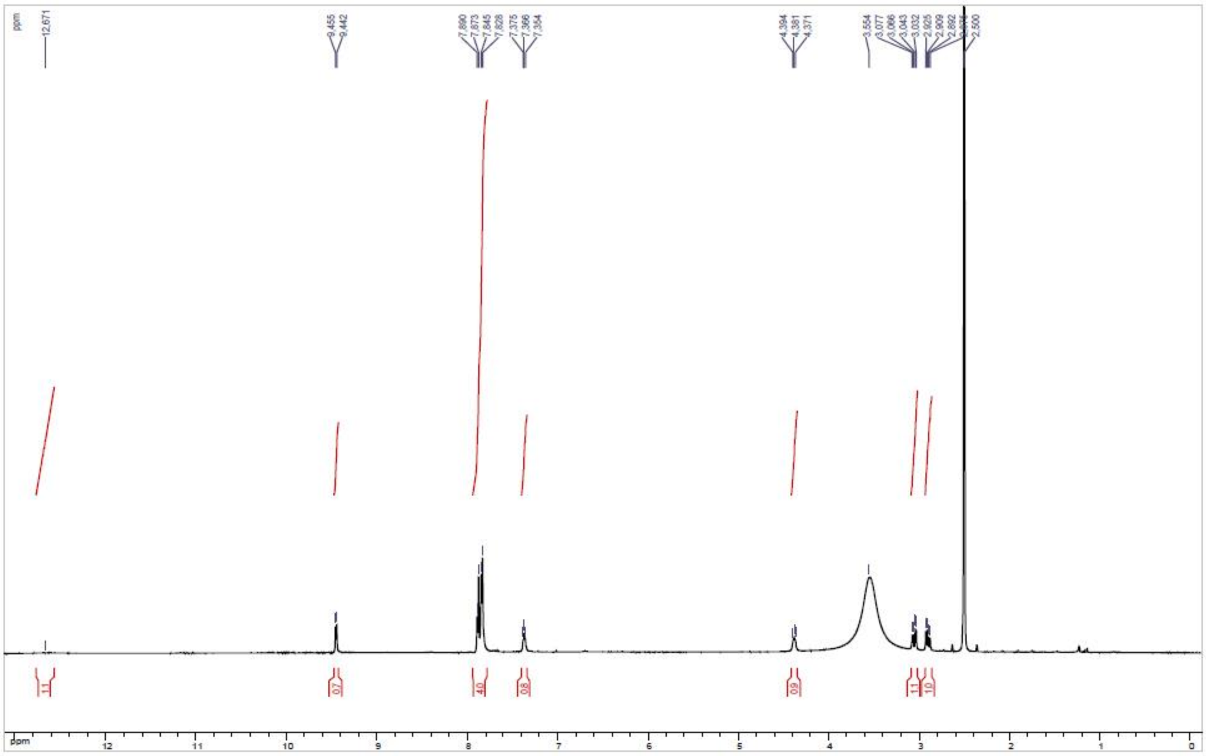

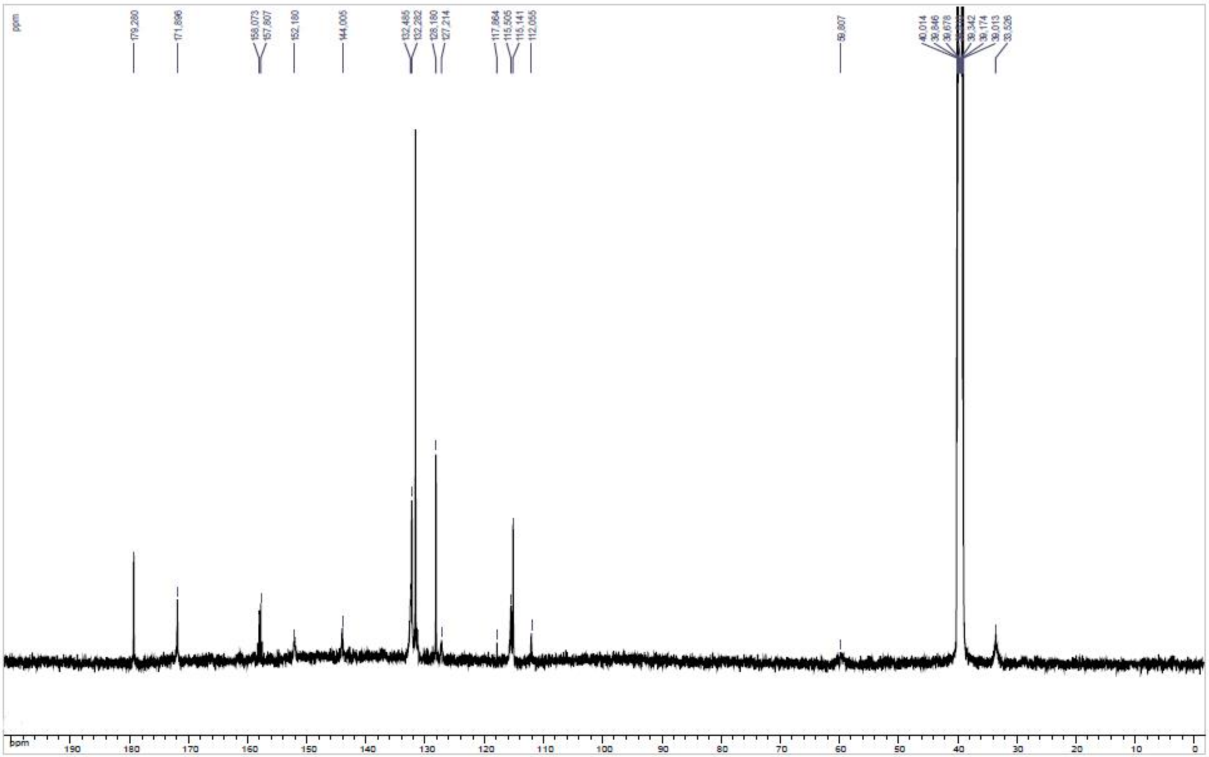

Compound **22** ($CDCl_3$)

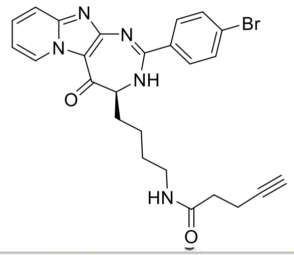

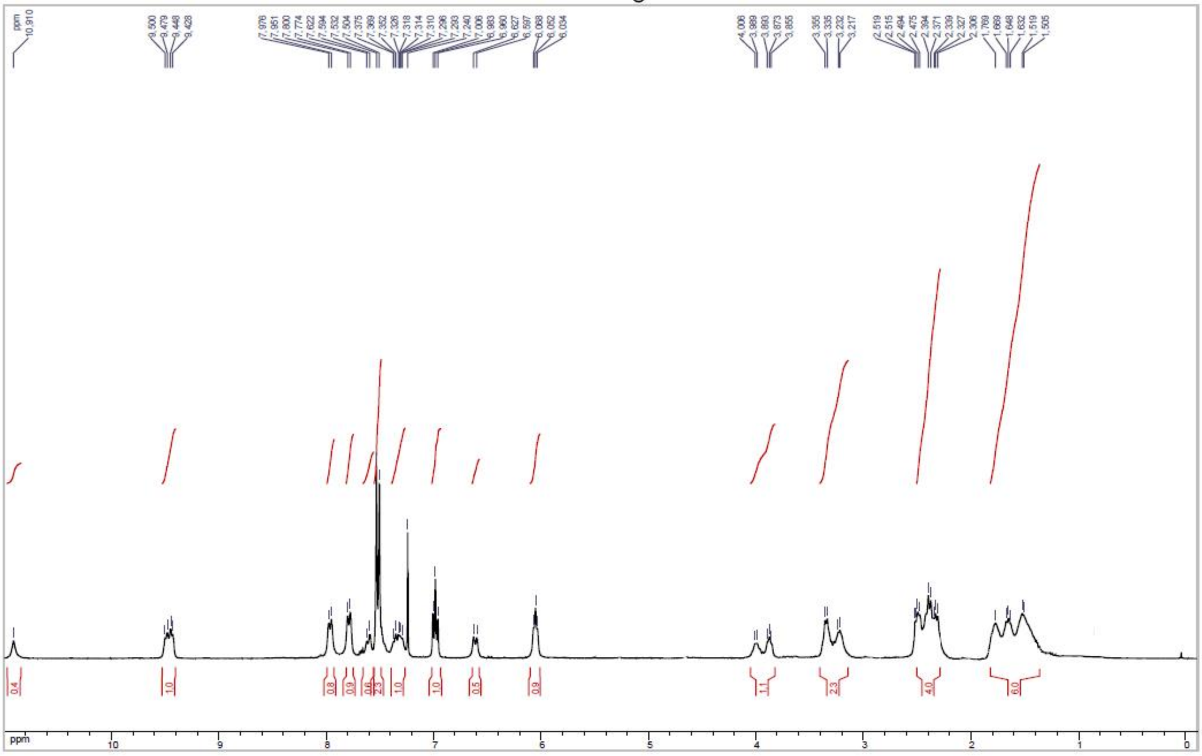

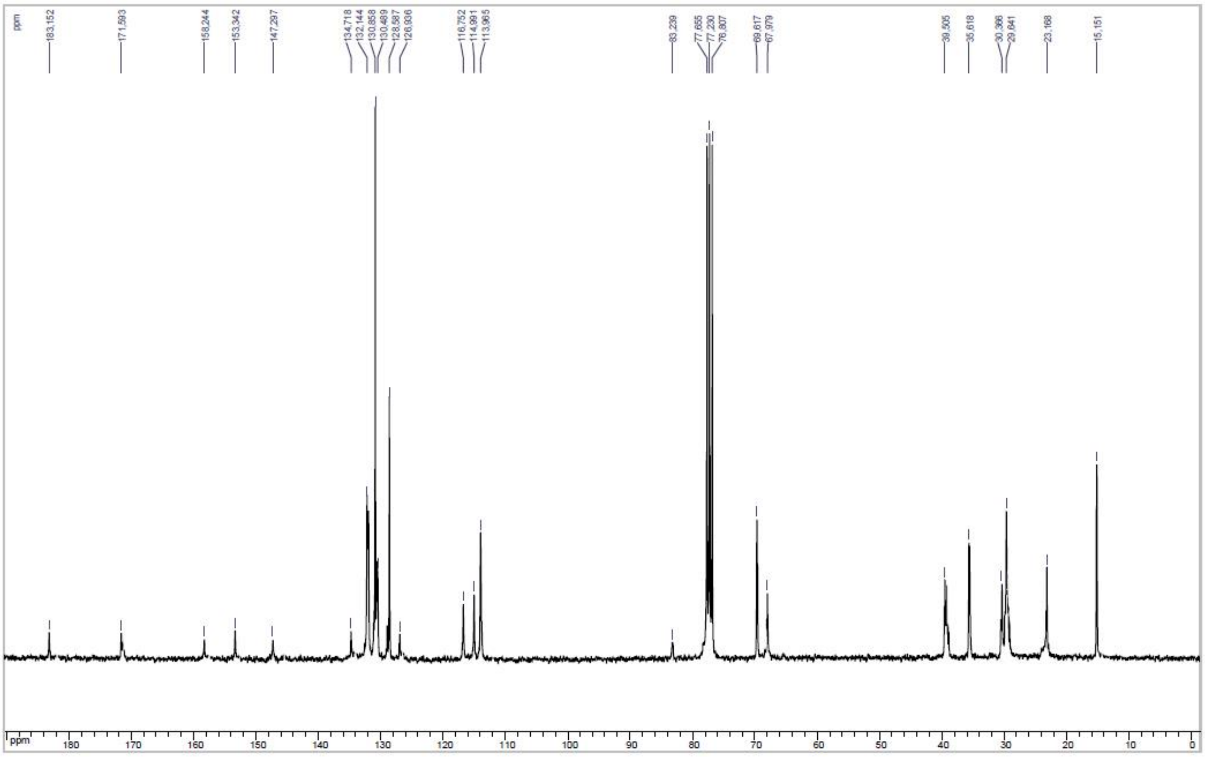

II. NCI screening data at 10 µM (SRB assay) - Table S1

Table S1. Evaluation of *in vitro* anti-cancer activity of JMV5038, compounds **5, 8** and **16** on the NCI 60-cell lines panel, after 48 h of exposure at the single dose of 10 µM of each tested compound. [1] NSCLC : Non-Small Cell Lung Cancer; [2] NT: not tested.

		**JMV5038**		**Compound 5**		**Compound 8**		**Compound 16**	
Panel	Cell line	G%	Mean G% - G%	G%	Mean G% - G%	G%	Mean G% - G%	G%	Mean G% - G%
Leukemia	CCRF-CEM	79,44	-25,20	97,39	-12,13	NT	-	97,94	-6,17
Leukemia	HL-60(TB)	NT[2]	-	NT	-	68,87	6,00	73,25	18,52
Leukemia	K-562	24,32	29,92	NT	-	81,21	-6,34		91,77
Leukemia	MOLT-4	57,51	-3,27	NT	-	83,46	-8,59	87,20	4,57
Leukemia	RPMI-8226	93,97	-39,73	NT	-	50,36	24,51		91,77
NSCLC[1]	A549/ATCC	54,66	-0,42	89,07	-3,81	66,77	8,10	75,16	16,61
NSCLC	EKVX	NT	-	81,81	3,45	74,24	0,63	79,58	12,19
NSCLC	HOP-62	55,84	-1,60	78,95	6,31	51,13	23,74	77,35	14,42
NSCLC	HOP-92	59,48	-5,24	102,26	-17,00	40,26	34,61	102,82	-11,05
NSCLC	NCI-H226	68,28	-14,04	89,14	-3,88	72,24	2,63	88,19	3,58
NSCLC	NCI-H23	53,67	0,57	87,07	-1,81	70,95	3,92	99,11	-7,34
NSCLC	NCI-H322M	73,95	-19,71	95,89	-10,63	64,81	10,06	91,36	0,41
NSCLC	NCI-H460	32,50	21,74	93,67	-8,41	85,19	-10,32	90,79	0,98
NSCLC	NCI-H522	34,81	19,43	71,94	13,32	95,32	-20,45	84,76	7,01
Colon Cancer	COLO 205	26,33	27,91	79,31	5,95	100,30	-25,43	107,19	-15,42
Colon Cancer	HCC-2998	69,04	-14,80	81,44	3,82	98,06	-23,19	112,38	-20,61
Colon Cancer	HCT-116	29,22	25,02	87,71	-2,45	72,13	2,74	92,81	-1,04
Colon Cancer	HCT-15	30,75	23,49	68,39	16,87	76,04	-1,17	98,33	-6,56
Colon Cancer	HT29	17,52	36,72	74,80	10,46	85,45	-10,58	105,90	-14,13
Colon Cancer	KM12	29,55	24,69	57,98	27,28	69,97	4,90	81,66	10,11
Colon Cancer	SW-620	37,96	16,28	71,90	13,36	75,57	-0,70	91,96	-0,19
CNS Cancer	SF-268	83,44	-29,20	97,61	-12,35	66,05	8,82	90,73	1,04

CNS Cancer	SF-295	40,88	13,36	93,44	-8,18	83,53	-8,66	95,79	-4,02
CNS Cancer	SF-539	74,64	-20,40	87,83	-2,57	76,74	-1,87	98,01	-6,24
CNS Cancer	SNB-19	82,62	-28,38	89,70	-4,44	79,82	-4,95	95,12	-3,35
CNS Cancer	SNB-75	50,16	4,08	70,35	14,91	14,51	60,36	87,65	4,12
CNS Cancer	U251	53,95	0,29	99,11	-13,85	87,34	-12,47	91,03	0,74
Melanoma	LOX IMVI	52,44	1,80	80,45	4,81	93,54	-18,67	86,21	5,56
Melanoma	MALME-3M	51,75	2,49	81,98	3,28	75,08	-0,21	114,50	-22,73
Melanoma	M14	31,76	22,48	81,98	3,28	91,62	-16,75	105,88	-14,11
Melanoma	MDA-MB-435	-13,02	67,26	12,62	72,64	75,04	-0,17	105,54	-13,77
Melanoma	SK-MEL-2	47,37	6,87	94,38	-9,12	94,95	-20,08	108,38	-16,61
Melanoma	SK-MEL-28	66,15	-11,91	94,39	-9,13	98,20	-23,33	109,90	-18,13
Melanoma	SK-MEL-5	31,90	22,34	70,86	14,40	61,95	12,92	87,94	3,83
Melanoma	UACC-257	69,95	-15,71	100,85	-15,59	98,55	-23,68	86,29	5,48
Melanoma	UACC-62	49,23	5,01	67,29	17,97	86,94	-12,07	79,83	11,94
Ovarian Cancer	IGROV1	43,08	11,16	74,50	10,76	59,98	14,89	21,61	70,16
Ovarian Cancer	OVCAR-3	17,29	36,95	97,54	-12,28	80,13	-5,26	103,28	-11,51
Ovarian Cancer	OVCAR-4	77,46	-23,22	88,58	-3,32	74,08	0,79	94,68	-2,91
Ovarian Cancer	OVCAR-5	82,38	-28,14	108,10	-22,84	107,69	-32,82	103,47	-11,70
Ovarian Cancer	OVCAR-8	72,52	-18,28	101,34	-16,08	84,54	-9,67	84,99	6,78
Ovarian Cancer	NCI/ADR-RES	39,37	14,87	73,34	11,92	77,71	-2,84	101,26	-9,49
Ovarian Cancer	SK-OV-3	72,02	-17,78	86,45	-1,19	67,29	7,58	85,99	5,78
Renal Cancer	786-0	83,10	-28,86	95,14	-9,88	89,59	-14,72	90,79	0,98
Renal Cancer	A498	69,88	-15,64	99,42	-14,16	61,33	13,54	82,87	8,90
Renal Cancer	ACHN	69,21	-14,97	84,77	0,49	79,59	-4,72	88,20	3,57
Renal Cancer	CAKI-1	44,15	10,09	74,77	10,49	35,11	39,76	84,51	7,26
Renal Cancer	RXF 393	68,18	-13,94	86,43	-1,17	NT	-	80,94	10,83
Renal Cancer	SN12C	91,77	-37,53	103,54	-18,28	84,11	-9,24	96,41	-4,64
Renal Cancer	TK-10	109,66	-55,42	133,27	-48,01	111,73	-36,86	129,91	-38,14
Renal Cancer	UO-31	62,01	-7,77	69,59	15,67	52,00	22,87	63,78	27,99

Prostate Cancer	PC-3	63,20	-8,96	96,23	-10,97	77,56	-2,69	102,28	-10,51
Prostate Cancer	DU-145	87,74	-33,50	103,64	-18,38	72,63	2,24	101,95	-10,18
Breast Cancer	MCF7	20,95	33,29	58,62	26,64	50,58	24,29	79,97	11,80
Breast Cancer	MDA-MB-231	52,71	1,53	80,35	4,91	67,93	6,94	75,04	16,73
Breast Cancer	HS 578T	71,34	-17,10	96,21	-10,95	59,47	15,40	93,28	-1,51
Breast Cancer	BT-549	49,32	4,92	95,37	-10,11	86,72	-11,85	105,59	-13,82
Breast Cancer	T-47D	67,23	-12,99	76,97	8,29	45,91	28,96	81,60	10,17
Breast Cancer	MDA-MB-468	-0,50	54,74	73,38	11,88	70,69	4,18	98,23	-6,46
**Mean**		54,24	-	85,26	-	74.87	-	91.77	-

III. DNA cell cycle analysis - Figure S1

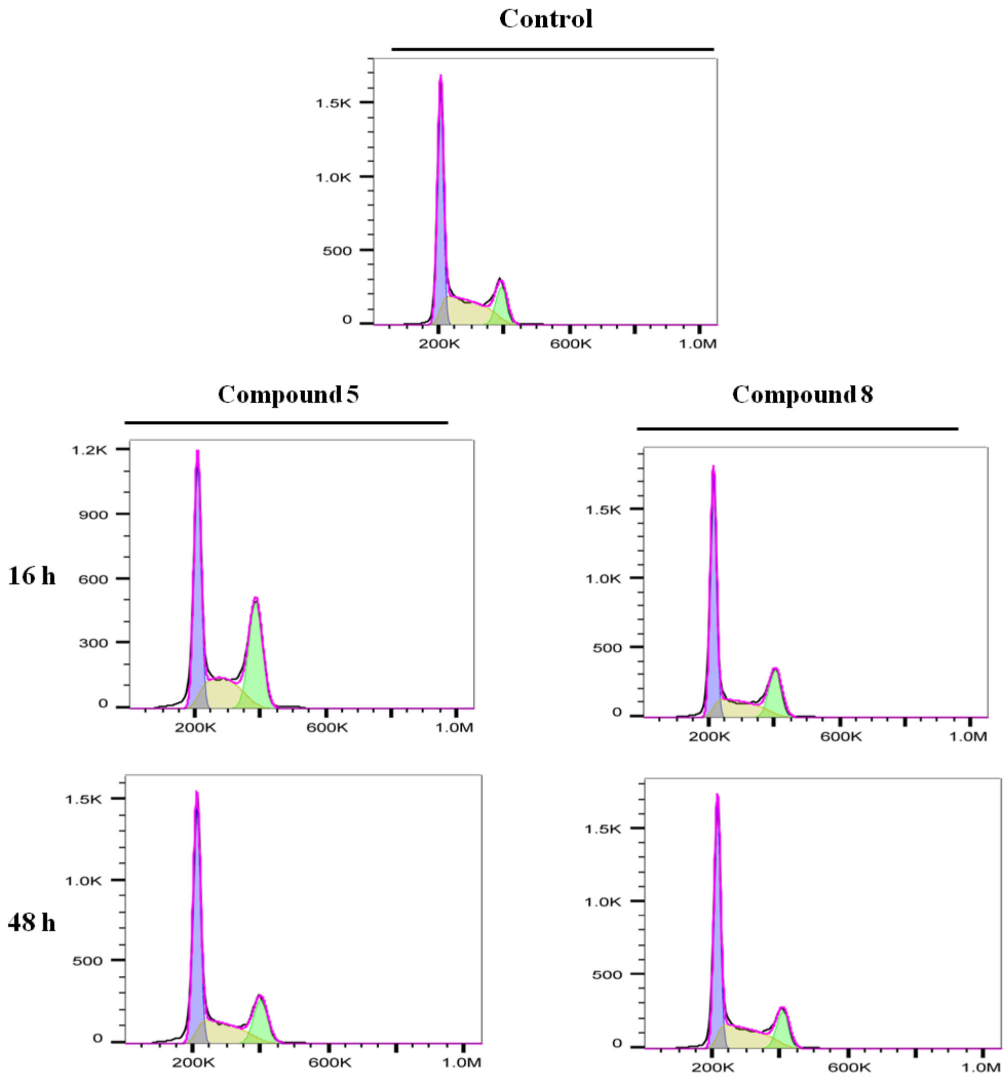

Figure S1: Cell cycle distribution by flow cytometry in MDA-MB-435 cells treated with control (DMSO) or compounds **5** or **8** at 5 µM for 16 and 48h.
